# Cloning, characterization, and inhibition of the novel β-carbonic anhydrase from parasitic blood fluke, *Schistosoma mansoni*

**DOI:** 10.1080/14756366.2023.2184299

**Published:** 2023-03-01

**Authors:** Susanna Haapanen, Andrea Angeli, Martti Tolvanen, Reza Zolfaghari Emameh, Claudiu T. Supuran, Seppo Parkkila

**Affiliations:** a Faculty of Medicine and Health Technology, Tampere University, Tampere, Finland; b Neurofarba Department, Sezione di Chimica Farmaceutica e Nutraceutica, Università degli Studi di Firenze, Sesto Fiorentino, Italy; c Department of Computing, University of Turku, Turku, Finland; d Department of Energy and Environmental Biotechnology, National Institute of Genetic Engineering and Biotechnology (NIGEB), Tehran, Iran; e Fimlab Ltd, Tampere University Hospital, Tampere, Finland

**Keywords:** Carbonic anhydrase, anti-parasitic agents, inhibitor, sulphonamide, *Schistosoma mansoni*

## Abstract

*Schistosoma mansoni* is an intestinal parasite with one β-class carbonic anhydrase, SmaBCA. We report the sequence enhancing, production, catalytic activity, and inhibition results of the recombinant SmaBCA. It showed significant catalytic activity on CO_2_ hydration *in vitro* with *k*_cat_ 1.38 × 10^5^ s^−1^ and *k*_cat_/*K_m_* 2.33 × 10^7^ M^−1^ s^−1^. Several sulphonamide inhibitors, from which many are clinically used, showed submicromolar or nanomolar inhibitory effects on SmaBCA. The most efficient inhibitor with a *K_I_* of 43.8 nM was 4-(2-amino-pyrimidine-4-yl)-benzenesulfonamide. Other effective inhibitors with *K_I_*s in the range of 79.4–95.9 nM were benzolamide, brinzolamide, topiramate, dorzolamide, saccharin, epacadostat, celecoxib, and famotidine. The other tested compounds showed at least micromolar range inhibition against SmaBCA. Our results introduce SmaBCA as a novel target for drug development against schistosomiasis, a highly prevalent parasitic disease.

## Introduction

*Schistosoma mansoni* is a parasitic blood fluke affecting ∼200 million people worldwide.^1^^,^^2^ It causes schistosomiasis and has been rated as the second most harmful parasite in the world; only malaria has been stated to cause more mortality.^3^^,^^4^ *Schistosoma mansoni* is endemic in Africa, South America, the Caribbean, and the Middle East.^5^ Schistosomiasis is an intestinal infection that produces acute symptoms of diarrhoea,^6^ abdominal pain,^6^ and fever.^7^

Patients are afflicted with *S. mansoni* as the larvae living in freshwater penetrate through healthy skin and travel via the bloodstream to the recipient’s liver.^8^ The larvae grow up and reach mesenteric veins to lay eggs.^9^ Some of the larvae reside in the liver arteries^10^ and can live for decades, causing granulomatous infection to the wall of the intestines as the eggs invade the gut wall.^2^^,^^5^ Chronic granulomatous infection occurs in the liver, serving as an incubator for developing larvae and trapping some of the eggs,^1^ and can lead to irreversible hepatic cirrhosis, especially if the affected person has chronic viral hepatitis, relatively common in the endemic areas of *S. mansoni*.^11^ The chronic infection can be cured only via medical intervention as the host’s immune system is unable to overcome the parasite.^10^

**Figure 1.**
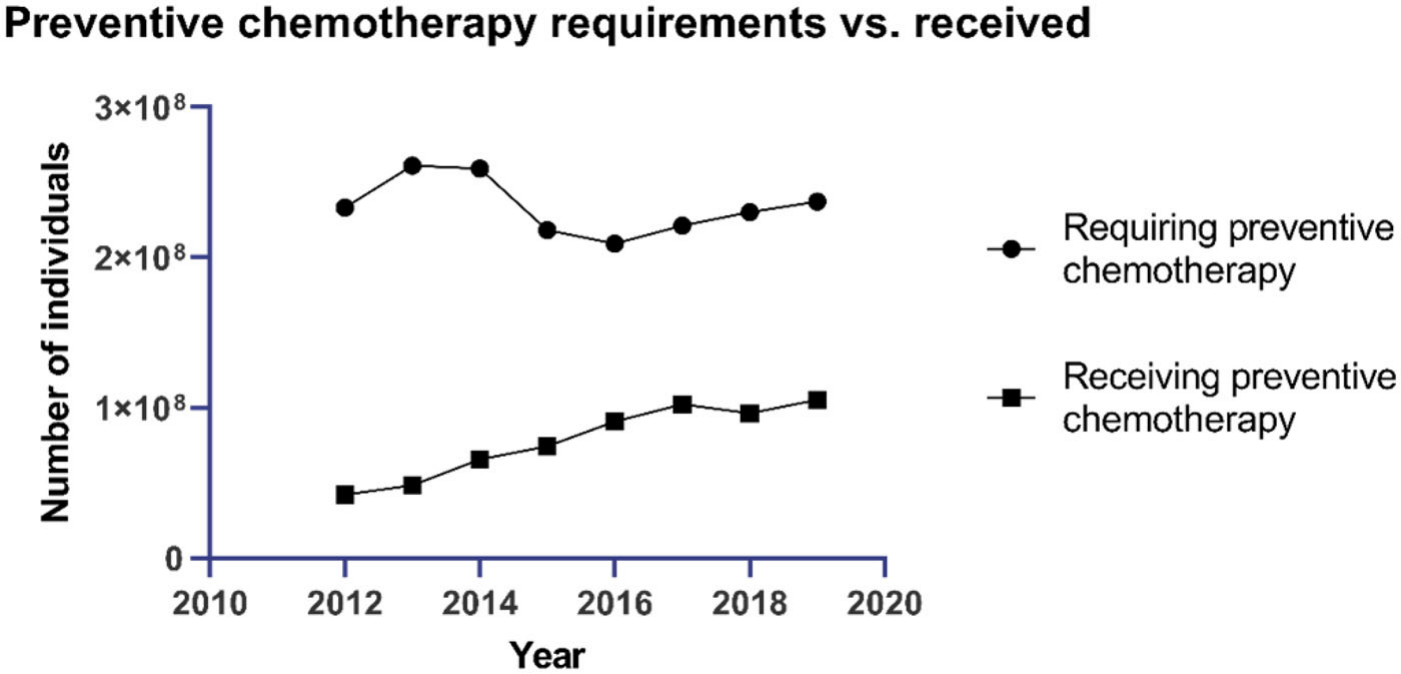
The worldwide requirement of preventive medication compared to the realised medication according to WHO [https://apps.who.int/neglected_diseases/ntddata/sch/sch.html, figure visualisation made by Susanna Haapanen using GraphPad Prism (1992–2020 GraphPad Software, LLC, version 9.0.0)].

There is no vaccine against schistosomiasis^12^^,^^13^ and the only currently known effective treatment against species of *Schistosoma*-genus parasites^14^ is executed with praziquantel.^10^ It is used as a preventive medicine and for treating patients,^15^ but millions of people still get infected yearly.^1^ Currently, the need for preventive chemotherapy is over two times higher than the number of individuals receiving the required medication (Figure 1). The pervasive use of praziquantel for schistosomiasis has led to rising resistance against the drug.^16^^,^^17^ Therefore, potential medicines with novel modes of action are urgently needed to fight this devastating disease.

In developing new drugs against parasitic diseases, carbonic anhydrases (CAs, EC 4.2.1.1) have recently emerged as potential targets.^18–20^ CAs catalyse the reversible hydration of CO_2_, maintaining homeostasis in tissues and fluids of the body. Eight distinct families of CAs have been presented in classifications based on protein structures and the active site. Some of the families have evolved as a result of long divergent evolution, but there are at least four independent ancestors.^21–23^ The α- and η-CAs share a common protein fold but they contain different catalytic residues in the active sites.^22^ The group of β-like-CAs includes ε-, θ-, and ζ-CAs (M. Tolvanen, unpublished observations). For γ-CAs and ι-CAs, no structural or evolutionary relatedness to other classes has been reported. On the contrary, δ-CAs show structural resemblance to α-CAs, although they are considered a family of their own.^24^^,^^25^ The earliest discovered enzyme forms are α-CAs found in humans, other animals, and many other pro- and eukaryotic cells.^26^ β-CAs are not found in vertebrates but are present in most prokaryotes, archaea, plants, fungi, and non-chordate metazoa.^26^^,^^27^ γ-CAs have been identified in some prokaryotes, fungi, plants, and single-cell eukaryotic organisms, such as amoebas.^27^^,^^28^ δ-CAs are widely distributed in marine phytoplankton.^29^ ζ-CAs are mainly present in diatoms,^30^ and η-CAs have been discovered in *Plasmodium*-parasites and in *Toxoplasma gondii*, thus far.^22^^,^^31^ θ-CAs have been found in marine diatoms, green algae, and bacteria,^32^ and the most recently found ι-CAs are widely found in bacteria, diatoms, green algae, and cyanobacteria.^23^^,^^33^ *S. mansoni* has only one β-CA but six α-CAs,^34^ one of which has been previously produced as a recombinant protein and tested for inhibition properties using various sulphonamide and anion class inhibitors.^35^ For instance, clorsulon, an antiparasitic sulphonamide, has a good inhibitory activity *in vitro*.^36^ This study is focussed on the *S. mansoni* β-CA, for which we use the abbreviation SmaBCA.

CA inhibitors of the sulphonamide class are already in use as drugs to treat clinical conditions, such as glaucoma,^37^^,^^38^ brain oedema,^39^ and mountain sickness.^40^^,^^41^ Ongoing research of new drug candidates for targeting different CA families aims at novel treatment of various diseases, such as cancer,^38^^,^^42^ neuropathic pain,^43^ migraine^43^ as well as infectious diseases.^42^^,^^44^ The clinically used inhibitors affect more than one CA isoform^45^ and can contribute to significant adverse side effects.^46^ The human genome has only α-CAs, and the absence of β-CAs provides an excellent opportunity to design and generate more specific anti-parasitic drugs, explicitly targeting the β-CAs. This study aimed to produce and characterise recombinant SmaBCA and to test the efficacy of selected sulphonamide- and anion-type CA inhibitors against this enzyme. The results could help to determine the best inhibitor candidates for further development as lead compounds of antiparasitic drugs.

## Materials and methods

### Sequence retrieval

At the start of this study, we identified *S. mansoni* β-CA sequences by Blast searches at NCBI (https://blast.ncbi.nlm.nih.gov/Blast.cgi) and UniProt (https://www.uniprot.org/blast), using β-CA from *Drosophila melanogaster*^47^ as a query sequence, finding XP_018647616.1 at NCBI and G4V6B2 at UniProt, identical sequences of 241 aa. When a DNA sequence coding for this sequence could not be produced, we looked at the original sequence more carefully.

Further BlastP searches were carried out against NCBI RefSeq proteins with the *S. mansoni* β-CA XP_018647616.1 as the query sequence, limiting the search to Platyhelminthes on May 14th, 2018. Selected hits were aligned using Clustal Omega (https://www.ebi.ac.uk/Tools/msa/clustalo/).^48^^,^^49^ An N-terminally incomplete *S. haematobium* protein XP_012795040.1 matched well with *S. mansoni* XP_018647616.1 but had a longer C-terminus, which resembles the C-termini of β-CA sequences of other parasites. This prompted us to revisit the gene model of XP_018647616.1 to see if a sequence coding for a similar C-terminal extension could also be found in the *S. mansoni* genome. Using the *S. haematobium* β-CA protein as a query sequence in a Blast search at metazoa.ensembl.org, we identified a sequence coding for a full-length *SmBCA*, which was used in the successful production of an active enzyme.

### Multiple sequence alignments (MSA)

In order to visualise sequence conservation in metazoan β-CAs for this article, we collected a new sequence set on 30 March 2022. *S. japonicum* KAH8855123.1 was used as a query sequence in BLAST at NCBI, with substitution matrix BLOSUM45, result list size of 5000, and taxonomy filter set to Metazoa. This resulted in 643 hits, filtered (with NCBI Blast result tools) for 85 % query coverage, leaving 520 sequences (https://github.com/MarttiT/S.-mansoni-BCA/blob/main/SmaBCA520%20blast%20hits%20Descriptions.xlsx). Incidentally, at this point, the RefSeq version of *S. mansoni* β-CA (XP_018647616.1) was too short to pass the coverage filter. The longest 35 sequences (of 998–2153 aa, all from rotifers) were removed. The remaining 485 sequences were aligned preliminarily using the Clustal Omega^48^^,^^49^ within SeaView 5.0.4.,^50^ and sequences with unique insertions or clearly mismatching sequences within conserved regions were manually deleted (using SeaView). The remaining 390 sequences were realigned with SeaView, and the resulting MSA (https://github.com/MarttiT/S.-mansoni-BCA/blob/main/SeaView%20MSA%20390.aln) was filtered for sequences with more than 90 % identity against any other sequence in the set. The Decrease redundancy tool at https://web.expasy.org/decrease_redundancy/ was used for this purpose (Cédric Notredame, unpublished), and a final realignment was performed on the obtained set of 162 protein sequences with Clustal Omega at EBI (https://www.ebi.ac.uk/Tools/msa/clustalo/)^49^ (Supplementary File 1).

A sequence logo was constructed to visualise this MSA using Berkeley WebLogo version 3.7.12 (https://weblogo.threeplusone.com/)^51^ with the parameters of 1st pos −112, logo from 1 to 310, no adjustment for composition, no error bars, colour scheme chemistry (Figure 6).

**Figure 2.**
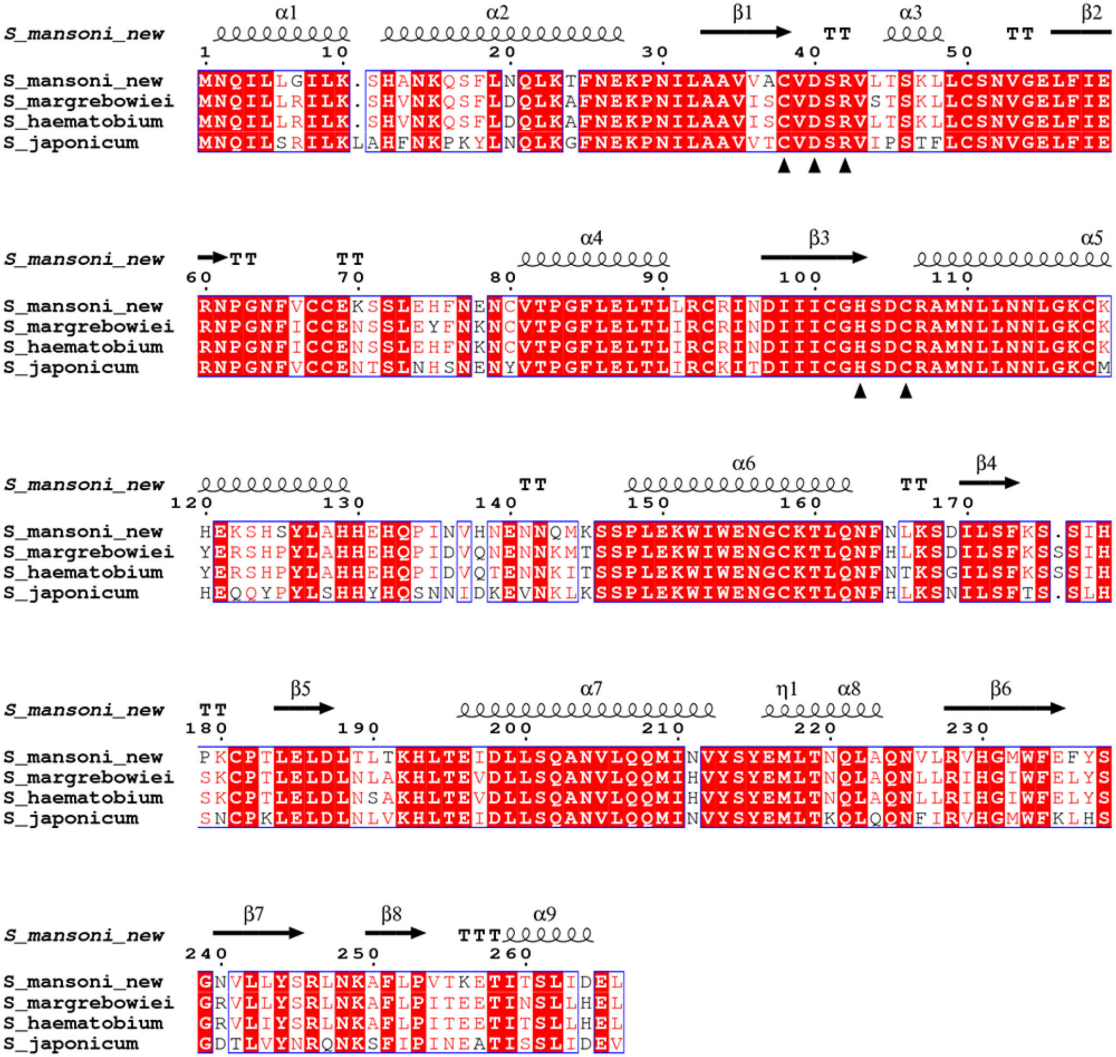
Comparison of the β-CA protein sequence of this study (S_mansoni_new) by sequence alignment to homologs from three other *Schistosoma* species. The conserved residues of the catalytic-site motifs, CXDXR and HXXC, are indicated with black triangles (C: Cys, D: Asp, H: His, R: Arg, X: any residue). Columns with fully conserved residues are shown as red with white letters. Boxed columns denote positions in which at least 75 % of residues are of a similar type, consisting of a total of 244 aa (91.7 %), of which 175 aa are totally conserved (65.8 %). The top line indicates the secondary structures of the AlphaFold model for *S. mansoni* β-CA. α: α-helices; β: β-strands; η: 3_10_-helices; T: turns.

To compare schistosomal β-CA sequences (Figure 2), we prepared a Clustal Omega alignment of the SmaBCA sequence of this study with *S. margrebowiei* VDO63334.1, *S. haematobium* KAH9593836.1, and *S. japonicum* TNN15731.1. This alignment was visualised using ESPript 3.0 at https://espript.ibcp.fr/ESPript/cgi-bin/ESPript.cgi.^52^ A global score cut-off of 0.75 used for colouring the blocks and secondary structure assignments for the top line were taken from the *S. mansoni* β-CA model retrieved from the AlphaFold protein structure database, https://alphafold.ebi.ac.uk/entry/A0A3Q0KBP5.^53^^,^^54^

### Dimer modelling

The monomer model retrieved from the AlphaFold protein structure database has a visual resemblance to the pea β-CA model (PDB 1ekj) in that the N-terminal alpha helices are detached from the catalytic domain of the monomer structure. In the full octamer model of 1ekj, we can see that these alpha helices wrap around another monomer to create the basic dimer unit, which is repeated as four copies. This inspired us to create a 3D dimer model for *SmaBCA* using ChimeraX (daily build 1.4.dev202202030703), developed by the UCSF Resource for Biocomputing, Visualisation, and Informatics (San Francisco, California, USA), supported in part by the National Institutes of Health. The AlphaFold multimer modelling interface^55^ was used to submit the prediction to run at Google Colab, giving two copies of our *SmaBCA* sequence as input, on 6 April 2022. The dimer model is available at https://github.com/MarttiT/S.-mansoni-BCA/blob/main/SmaBCA%20dimer.pdb.

### Vector construction, protein production, and purification

The same production procedure was followed both for the unsuccessful production of the protein corresponding to UniProt G4V6B2 and our amended sequence with a full-length last exon. The amended sequence contains the modifications to C-terminus as described above as well as the silent mutations to prevent Rho-independent termination site forming, on the contrary, to the unsuccessful production of the protein to which the sequence was unmodified.

Since some coding genes may contain termination codes in the middle of mRNA coding sequences and lead to early transcription termination and the consequent production of immature mRNA, a prediction approach was performed using ARNold (http://rssf.i2bc.paris-saclay.fr/toolbox/arnold/) web tool^56^ to find Rho-independent transcription termination sites on the *SmaBCA* gene (NCBI gene ID: 8342150). To prevent the formation of immature mRNA for SmaBCA, five silent mutations were introduced to change the nucleotides of Rho-independent termination sites to other nucleotides.

The finalised sequence of the insert was sent to GeneArt (Invitrogen, Regensburg, Germany), where a modified plasmid vector, pBVboost, was constructed.^57^

The plasmid vector was prepared according to the manufacturer’s instructions and then transformed with heat shock into BL21 StarTM (DE3) cells (Invitrogen, Carlsbad, USA), as described previously.^58^ The production of recombinant protein was executed manually in LB broth (Sigma-Aldrich, St. Louis, MO, USA) with 1:1000 gentamicin (Sigma-Aldrich) and 1:100 glycerol (VWR International, Radnor, PA, USA)/glucose (Sigma-Aldrich) as proposed in Kopp et al.^59^ at +37 degrees and shaking with 200 rpm. Both glycerol and glucose proved to be equally effective additives in the growth medium to reduce the number of impurities. The OD (Fisher Scientific Colorimeter Model 45 (WA12173), Fisherbrand, Thermo Fisher Scientific, Waltham, MA, USA) was measured and at the OD 0.4–0.5 1 M isopropyl-β-D-thiogalactopyranoside (IPGT, Thermo Fisher Scientific) was added in relation of 1:1000 to LB medium. Growth continued overnight and was terminated the next day by pelleting the cells by centrifugation at 5000 × g for 20 min resulting in a total incubation and production time of 24 h.

A few alterations were made to the purification protocol compared to Haapanen et al.^58^ We used 50 mM Na_2_HPO_4_ (Sigma-Aldrich), 0.5 M NaCl (VWR International), and 50 mM imidazole (Sigma-Aldrich) (pH 8.0) as a binding buffer and washed the nickel resin (HisPurTM Ni-NTA Resin, Thermo Fisher Scientific) with 15 × 25 ml of 50 mM Na_2_HPO_4_, 0.5 M NaCl, 50 mM imidazole, and 20 % glycerol (pH 8.0) with EMD Millipore™ vacuum filtering flask (Merck, Kenilworth, NJ, USA) and filter paper (pore size 2 µm, Whatman™, Maidstone, UK). The glycerol was washed off with 10 × 25 ml of binding buffer with the same vacuum filtering flask. Subsequently, the resin was collected into an empty column (Maxi Columns, G-Biosciences, St. Louis, MO, USA). The protein was eluted from the resin with elution buffer (50 mM Na_2_HPO_4_, 0.5 M NaCl, and 350 mM imidazole, pH 8.5). Re-purification was performed twice, similarly to the initial purification, except using 50 mM Na_2_HPO_4_, 0.5 M NaCl (pH 8.0) as a binding buffer to make the imidazole concentration under 20 mM. The purity of the collected fractions was verified with 12 % SDS-PAGE (sodium dodecyl sulfate-polyacrylamide gel electrophoresis) under reducing conditions using Precision Plus ProteinTM Standards Dual Colour (Bio-Rad Laboratories, Inc., Hercules, CA, USA) as a standard molecular weight (MW) marker. The MW marker and bands were visualised with PageBlue™ Protein Staining Solution (Thermo Fisher Scientific) on the gel by letting the gel stain for 30 min and then washing with distilled water. The bands were confirmed to be SmaBCA with mass spectrometry (data not shown).

### CA enzyme activity and inhibition

Before activity measurements, the buffer was changed into 50 mM TRIS (VWR International) (pH 8.5) with 10 kDa Vivaspin^®^ Turbo 15 centrifugal concentrators (Sartorius™, Göttingen, Germany) at 4000 × g at +4°C. The CA-catalysed CO_2_ hydration activity was measured using an Applied Photophysics stopped-flow instrument.^60^ The measurement protocol was identical to the previously described in Berrino et al.^61^

## Results

To produce the recombinant protein of *SmaBCA*, an insert sequence was retrieved from the National Centre of Biotechnology Information (NCBI Reference Sequence: XM_018793067.1). It was included in our construct because it was the only β-CA of *S. mansoni* available in sequence databases in 2018 (also represented in UniProt protein entry G4V6B2). We did not obtain any protein from recombinant production using this original sequence. Upon closer inspection, the sequence seemed to have an incorrectly predicted splice site in the actual last exon, joined to a superfluous exon, coding only for three residues instead of extending the last exon up to a stop codon. The full-length sequence for the last exon was discovered in the *S. mansoni* genome by searching with other schistosomal β-CA sequences. The amended last exon coded for 28 additional amino acids (aa), highly similar to the search query sequences. This sequence also led to the successful production of the active enzyme described in this study. A full-length sequence of *SmaBCA* was added in UniProt after we had already produced our protein (A0A3Q0KBP5, 13 February 2019). This sequence is probably derived from a later genome version than ours (which came from *S. mansoni* genome assembly ASM23792v2 as of 14 May 2018 at metazoa.ensembl.org), and it differs by one amino acid substitution (N264D) from our sequence. This is a variable site even within *Schistosoma* species (Figure 2).

**Figure 3.**
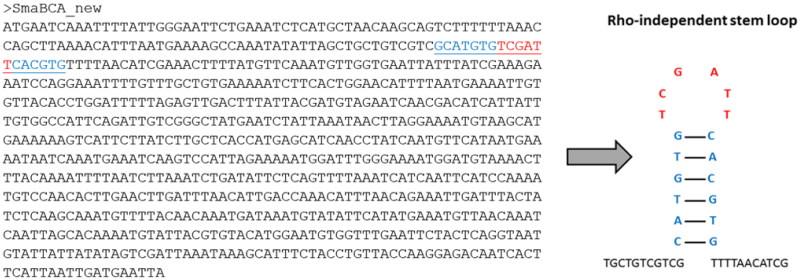
The coding sequence of the β-CA gene from *S. mansoni*. The predicted Rho-independent transcription termination site is marked in underlined blue and red text in the sequence, and the corresponding Rho-independent stem-loop is shown on the right.

Our analysis also revealed that the *SmaBCA* gene sequence contains a Rho-independent transcription termination site, a GC-rich area in the sequence causing loop formation and detachment of RNA polymerase, which introduces an early termination of transcription in bacteria and consequently a non-functional messenger RNA (mRNA) (Figure 3). To enable the recombinant production in *Escherichia coli*, we chose to create single nucleotide mutations in the coding sequence to disrupt base pairs in the stem (blue in Figure 3) without any effect on the translated amino acid sequence.

**Figure 4.**
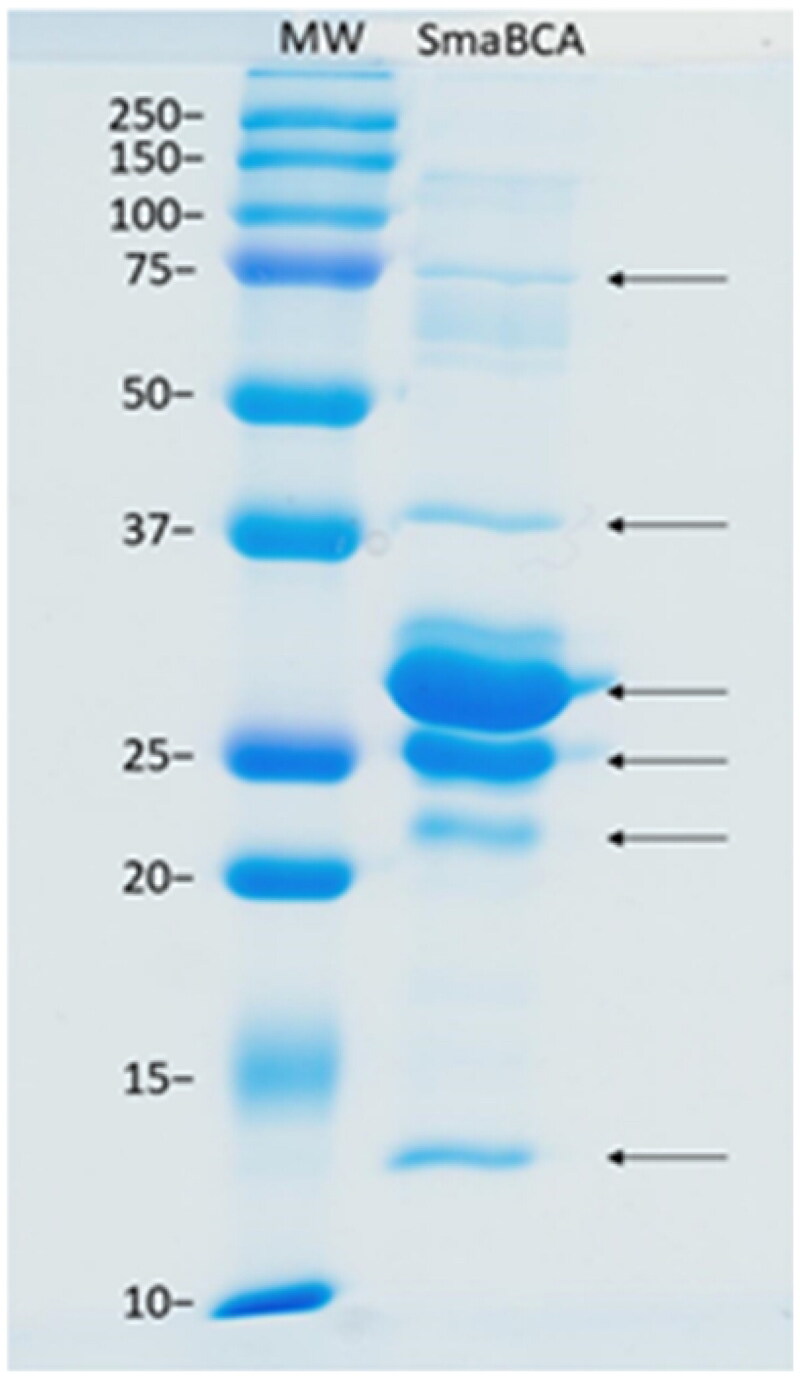
SDS-PAGE of β-CA of *S. mansoni* (SmaBCA) showing six polypeptide bands confirmed to represent SmaBCA by mass-spectrometry (data not shown). Right lane: The most intense bands of 25 and 29 kDa are the main forms of the expressed protein, and the additional polypeptides either are degraded forms (13 and 22 kDa) or oligomers (38 and 75 kDa). Left lane: Standard molecular weight (MW) markers in kDa.

As a result of recombinant protein production, we obtained six protein bands of ∼13, 22, 25, 29, 38, and 75 kDa in size (Figure 4). All these bands represented different forms of SmaBCA as confirmed by mass spectrometry (data not shown). The calculated molecular weight of the translated coding sequence is 30.4 kDa, not accounting for any post-translational modifications.

Kinetic analysis of SmaBCA showed that the enzyme (with His-tag) is moderately active with *k*_cat_ 1.38 × 10^5^ s^−1^, *K_m_* 5.92 mM, and *k*_cat_/*K_m_* 2.33 × 10^7^ M^−1^ s^−1^. Several sulphonamide and anion inhibitors^46^^,^^62^^,^^63^ were tested to evaluate their inhibitory properties against SmaBCA. The most efficient inhibitors showed submicromolar or nanomolar inhibitory effects on SmaBCA (Table 1 and Figure 5). The most efficient inhibitor with a *K_I_* of 43.8 nM was 4-(2-amino-pyrimidine-4-yl)-benzenesulfonamide (compound 19). Other effective inhibitors included several clinically used drugs. Benzolamide (BZA), brinzolamide (BRZ), topiramate (TPM), dorzolamide (DZA), saccharin (SAC), epacadostat (EPT), celecoxib (CLX), and famotidine (FAM) showed *K_I_*s in the range of 79.4–95.9 nM. The other tested compounds inhibited SmaBCA at micromolar or millimolar concentrations.

Figure 5.Chemical structures of sulphonamide (1–24) and sulphonamide/sulfamate derivatives (AAZ-EPT) tested as inhibitors against β-CA of *Schistosoma mansoni* (SmaBCA) in this study.

**Figure:**
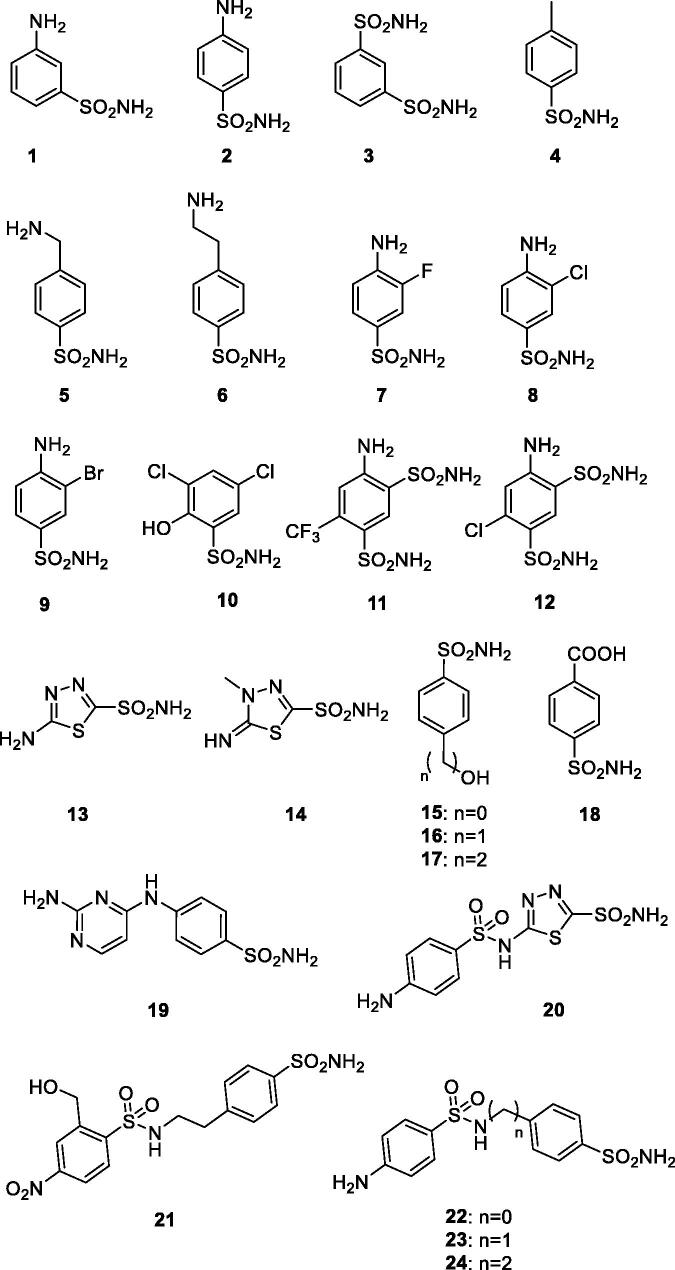

**Figure:**
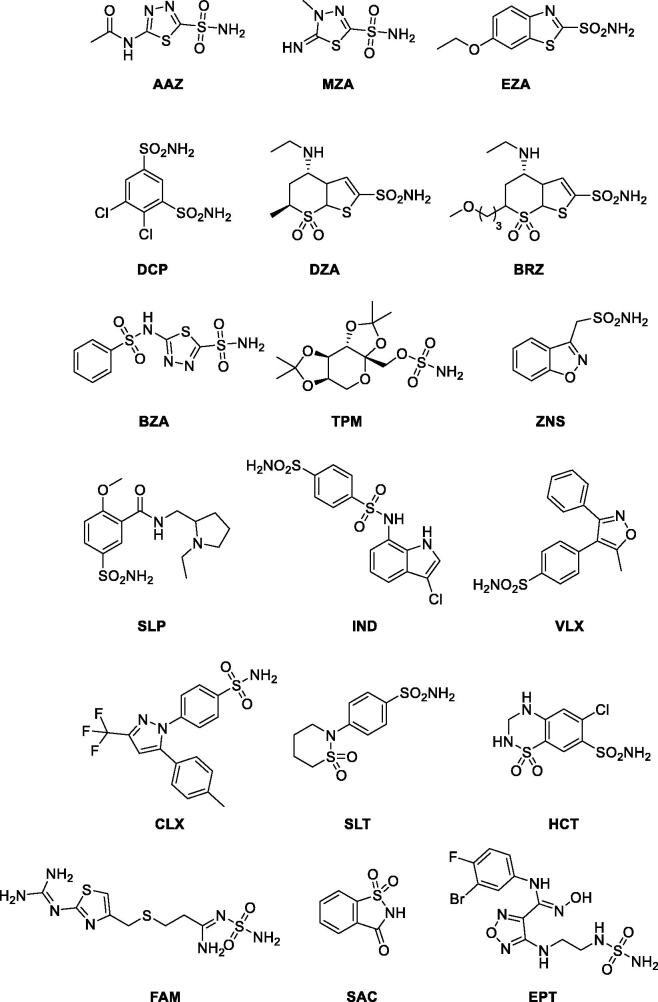

**Table 1.** Inhibition data for SmaBCA with sulphonamide analogs 1–24, clinically used compounds, and anions.

Compound	SmaBCA	Compound	SmaBCA
	*K_I_* (µM)*		*K_I_* (µM)*
1	1.830	BRZ	0.083
2	2.516	BZA	0.079
3	1.556	TPM	0.083
4	0.776	NO_2_^−^	>10 000
5	0.788	NO_3_^−^	2270
6	0.327	HCO_3_^−^	7840
7	0.872	CO_3_^2−^	740
8	0.372	HSO_3_^−^	4260
9	0.960	SO_4_^2−^	3720
10	0.935	F^−^	6280
11	2.040	Cl^−^	2850
12	0.417	Br^−^	2840
13	0.314	I^−^	840
14	0.375	CNO^−^	890
15	0.982	SCN^−^	930
16	0.600	HS^−^	820
17	0.346	CN^−^	930
18	1.043	N_3_^−^	800
19	0.044	Sulfamide	8
20	0.316	Sulfamic acid	40
21	0.255	Phenylarsonic acid	20
22	0.378	Phenylboronic acid	520
23	0.241	SnO_3_^2−^	960
24	0.750	SeO_4_^2−^	3490
SLP	0.254	TeO_4_^2−^	4900
IND	0.812	OsO_5_^2−^	580
ZNS	0.521	P_2_O_7_^2−^	>10 000
CLX	0.092	V_2_O_7_^2−^	>10 000
VLX	0.474	B_4_O_7_^2−^	4300
SLT	0.758	ReO_4_^−^	9090
SAC	0.091	RuO_4_^−^	3650
HCT	0.918	S_2_O_8_^2−^	>10 000
FAM	0.096	SeCN^−^	220
DCP	0.545	NH(SO_3_)^2−^	>10 000
EPT	0.092	FSO_3_^−^	>10 000
AAZ	0.286	CS_3_^2−^	3330
MZA	0.210	Et_2_NCS_2_^−^	420
EZA	0.246	PF_6_^−^	>10 000
DZA	0.090	Triflate	>10 000

*Mean from three different assays, by a stopped-flow technique (errors were in the range of ±5–10% of the reported values).

Our protein sequence was aligned with β-CA sequences of other *Schistosoma* species, as shown in Figure 2. All these sequences are highly similar, with identities to the protein of this study ranging from 75.6 to 86.8 %. The identity percentages are even higher in the protein core and in the active site, which suggests that any drugs developed to inhibit SmaBCA would also be inhibitory for β-CAs of other *Schistosoma* species.

**Figure 6.**
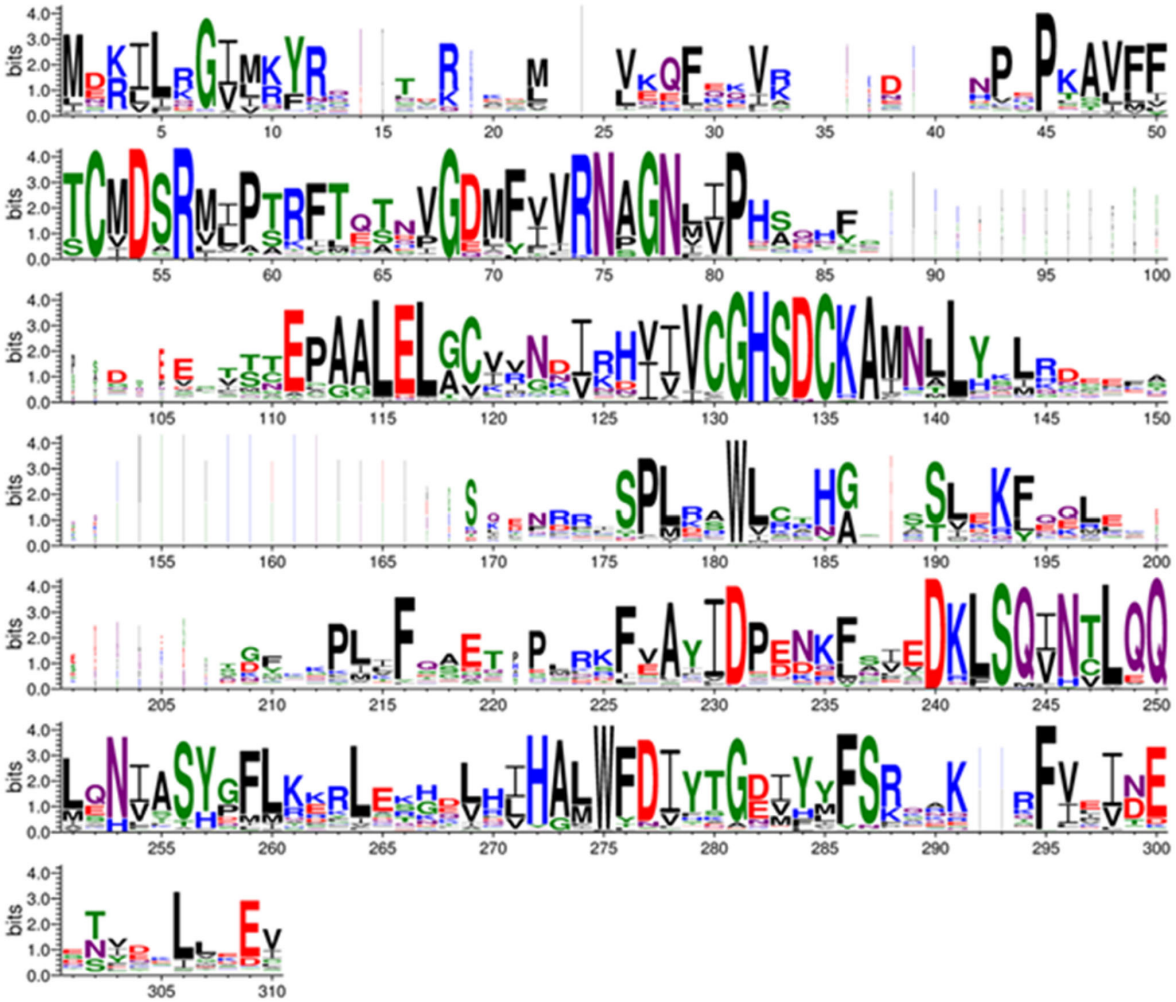
Comparison of metazoan β-CA proteins. This sequence logo represents an MSA of 162 sequences, cropped at both ends to show only the extent of the SmaBCA sequence. The height of each stack of letters represents the conservation (information content) of each column in the alignment. The width of the letters represents the number of non-gap characters in each column (i.e. very narrow letters indicate positions in which only few of the aligned proteins have any sequence).

The sequence logo of Figure 6 extracts information from a multiple sequence alignment (MSA) of 162 metazoan β-CA sequences showing 19 perfectly conserved aa sites and 26 almost conserved sites. Of course, we find highly conserved areas at the motifs, which are part of the active site (CXDXR and HXXC), and additionally, at columns 68–81 and 240–250. The gap region at columns 151–168 in the MSA (Figure 6) is due to an insertion seen only in Schistosoma sequences of our sequence set. The entire MSA is provided as Supplementary File S1. More data is available as noted in the Experimental procedures.

**Figure 7.**
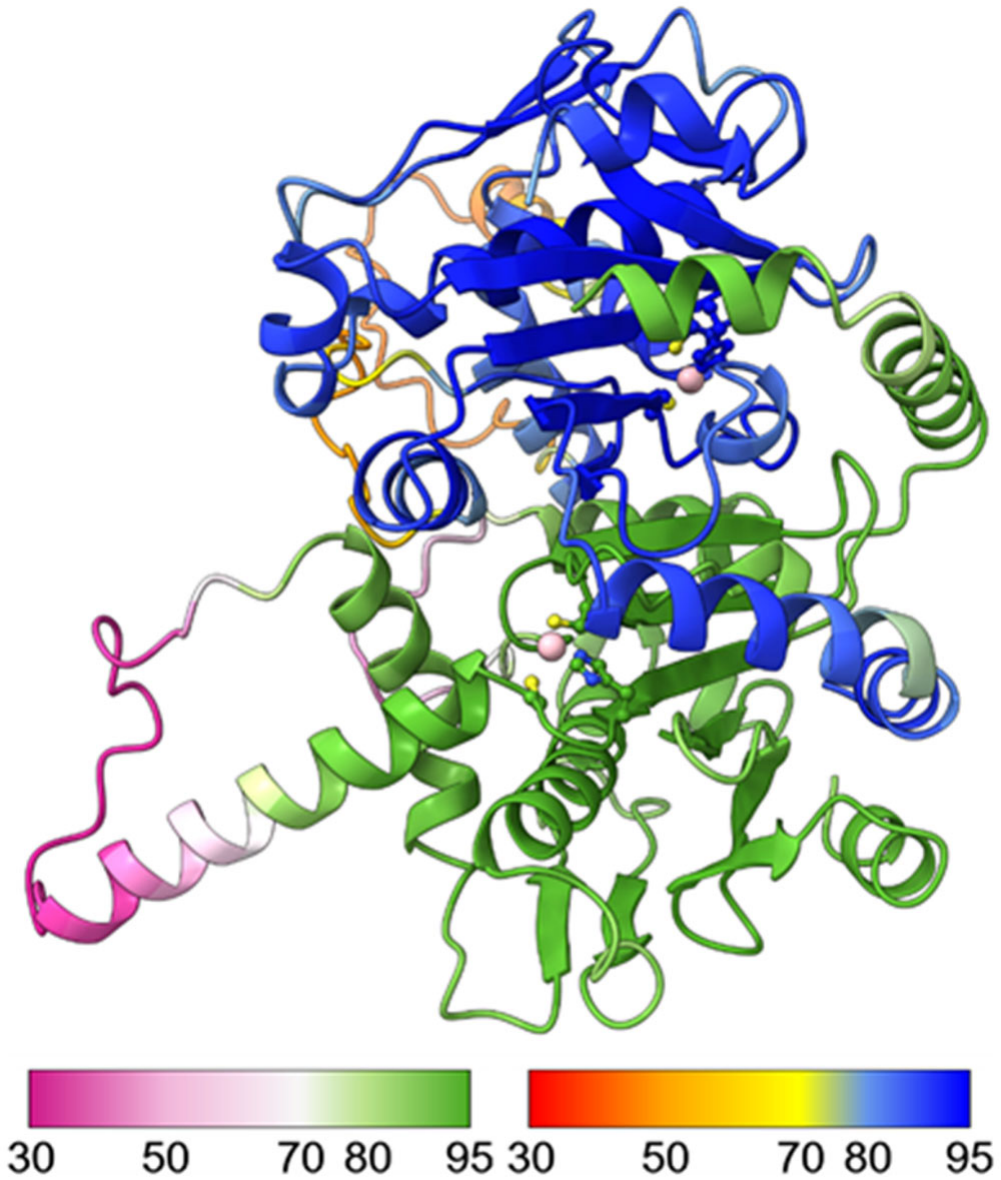
Molecular model of a hypothetical SmBCA dimer, constructed using AlphaFold multimer. Colouring is shown based on the per-residue confidence score (pLDDT), with two different palettes, as shown in the colour keys at the bottom left and right, for the bottom and top monomer, respectively. Metal-binding residues at the catalytic site (Cys 38, His 103, and Cys 106) are shown in balls-and-sticks style. The zinc ion at the catalytic site is displayed in pink.

We built a computational 3D model of the SmaBCA dimer using AlphaFold multimer. In this model, the alpha-helical segments in the N-terminus are positioned along the side of the other monomer, similar to the dimers of *Pisum sativum* (pea) β-CA (PDB 1ekj).^64^ These segments are also present in the AlphaFold monomer model (https://alphafold.ebi.ac.uk/entry/A0A3Q0KBP5), in which their position away from the core of the protein looks odd, but in the dimer model (Figure 7) it makes perfect sense. The orientation between the monomers in our model is nearly identical to pea β-CA dimers (1ekj, e.g. chains C and D, data not shown).

AlphaFold gives a confidence score (predicted local distance difference test, pLDDT) to the position of each residue in the model, pLDDT >90 indicating “very high confidence” and pLDDT >70 “confident”. In our model, two regions (69–79 and 119–145) have pLDDT values <70. They correspond to regions of low conservation and insertions of variable lengths in our MSA of metazoan β-CA sequences (columns 83–108 and 148–174 in Figure 6). These two regions are also slightly different between the AlphaFold database monomer model and our dimer model (data not shown).

## Discussion

Schistosomiasis causes high morbidity in tropical and subtropical countries, and despite the treatment with praziquantel,^65^ the infection remains a significant health problem.^66^ Unfortunately, there are already initial signs of developing resistance against praziquantel^67^ which emphasises the need for novel medication. In this study, we cloned the SmaBCA and found many already clinically used CA inhibitors with significant inhibitory effects against SmaBCA.

SmaBCA has a similar distribution of bands in SDS-PAGE as β-CAs from *Entamoeba histolytica* (EhiCA)^58^ and *Trichomonas vaginalis*^57^ before His-tag removal: they all contain the major dual-band at a size which is predicted according to the aa chain composition. However, the dual-band formation disappears from β-CAs of *T. vaginalis* as the His-tag is removed, contrary to EhiCA, which retains the double band appearance after the cleavage of His-tag.

SmaBCA shows enzyme activity with *k*_cat_ 1.38 × 10^5^ s^−1^ and *k*_cat_/*K_m_* 2.33 × 10^7^ M^−1^ s^−1^, within a similar range compared with the β-CAs from *T. vaginalis* (*k*_cat_ 4.9 × 10^5^ and *k*_cat_/*K_m_* 8.0 × 10^7^),^57^ *Leishmania donovani chagasi* (*k*_cat_ 9.35 × 10^5^ s^−1^ and *k*_cat_/*K_m_* 5.9 × 10^7^ s^−1^M^−1^),^68^ *Ascaris lumbricoides* (*k*_cat_ 6.0 × 10^5^ s^−1^, *k*_cat_/*K_m_* 4.3 × 10^7^ M^−1^ s^−1^),^69^ and EhiCA (*k*_cat_ of 6.7 × 10^5^ s^−1^ and a *k*_cat_/*K_m_* of 8.9 × 10^7^ M^−1^ × s^−1^),^58^ demonstrating the possibly crucial role in the metabolism of the organisms, as the magnitude of activity is considered from moderate (SmaBCA) to high (β-CAs from *T. vaginalis*, *Leishmania donovani chagasi*, *Ascaris lumbricoides*, and EhiCA) compared to human CA I (moderate, *k*_cat_ 2.0 × 10^5^ and *k*_cat_/*K_m_* 5.0 × 10^7^), for instance.

Schistosomiasis and amoebiasis (intestinal infection caused by *E. histolytica*) are endemic in the same areas of the world^70^ and coinfections are not unusual^71^^,^,^72^ leading to the conclusion of achievable benefits from treating the infections with only one drug: less adverse side effects for the patient, better treatment compliance, and lower costs for the society. SmaBCA and EhiCA are both well-inhibited with many anion and sulphonamide derivatives. The most effective ones for EhiCA are 4-hydroxymethyl/ethyl-benzenesulfonamides (compounds 16 and 17) with *K_I_* values of 89 and 36 nM, respectively,^63^ with good inhibition activity against SmaBCA with K_I_s of 600 and 346 nM, respectively. They are weak in inhibiting human CA II (K_I_ of 125 nM) and almost inefficient inhibitors of human CA I (*K_I_* of 21 µM),^63^ indicating a slight parasite selectivity. This kind of inhibitors could potentially have minimal side effects on humans. Other nanomolar range inhibitors are 4-(2-aminoethyl)benzenesulfonamide (compound 6), 4-((2-amino-4-pyrimidinyl)amino)benzenesulfonamide (compound 19), and acetazolamide (AAZ) with *K_I_*s of 509–798 nM for EhiCA and *K_I_*s of 44–286 nM for SmaBCA, from which acetazolamide is already in clinical use.^73–76^ With low micromolar range are sulfamide and phenylarsonic acid with *K_I_*s of 28–38 and 8–20 µM, for EhiCA and SmaBCA, respectively. The many agents with good inhibitory activity demonstrate the possibilities for developing CA inhibitors as anti-parasitic drugs against both enzymes of these parasites.

Aa sequences of SmaBCA and β-CAs of other *Schistosoma* species are highly similar, as shown by MSA (Figure 2). In particular, the essential parts (the protein core and the active site) of the β-CA sequences are highly conserved, hence, opening an exciting opportunity to find functioning CA inhibitor-based drugs effective against all the species as praziquantel nowadays is. To our knowledge, only one other *Schistosoma* species, *S. japonicum*, has had its only β-CA produced as a recombinant protein previously.^77^ Cong-Hui *et al*. produced 38 kDa recombinant protein with CA activity, but they did not make any comparison to β-CAs of other *Schistosoma* species. *S. mansoni* and *S. japonicum* are genetically distinct as they were separated as their own phylogenetic branches ∼14 million years ago,^78^ and they are endemic in different parts of the world: *S. japonicum* in South-East Asia and *S. mansoni* mainly in Africa, South America, the Caribbean, and Middle East, both causing similar intestinal infections.^1^^,^^5^ A new universal anti-schistosomal agent could have clinical value in very large areas covering most of the globe.

The β-CA of *S. mansoni* is a promising target for the development of new anti-schistosomal drugs. In this study, we produced a novel SmaBCA recombinant protein, tested it against different CA inhibitors, leading to the discovery of several well-inhibiting compounds, from which some are already used in treating other conditions. Based on structural and sequence analyses, we also propose that it is feasible to develop one universally functional anti-parasitic drug against several *Schistosoma* species which could also be effective against other parasites, such as *Entamoeba histolytica*.

## Supplementary Material

Supplemental Material

## Data Availability

The data that support the findings of this study are openly available in MarttiT/S.-mansoni-BCA at https://github.com/MarttiT/S.-mansoni-BCA.
