# Supplementary material for: Cloning, characterization, and inhibition of the novel β-carbonic anhydrase from parasitic blood fluke, Schistosoma mansoni
Source: J Enzyme Inhib Med Chem. 2023 Mar 1;38(1):2184299. doi: 10.1080/14756366.2023.2184299 (PMC9980027; doi:10.1080/14756366.2023.2184299)
Supplement: Supplemental Material [file IENZ_A_2184299_SM6325.pdf]

```
Alignment: C:\Users\meetol\Dropbox\CA_projects_Martti\Beta CAs\Parasites, for Susanna\decreased3.aln
Seaview [blocks=10 fontsize=8 A4] on Fri Nov 18 12:41:18 2022

                1
KAH8855123.1    ---------- ---------- ---------- ---------- ---------- ---------- --------- ---------- ----------
KAH9593836.1    ---------- ---------- ---------- ---------- ---------- ---------- --------- ---------- ----------
VDP71164.1      ---------- -------MEN KKLSNQLPGG RVG--NMLAF CSRDLGFKPH ---------- --------- ---------- --------LG
TPP65121.1      ---------- ---------- ---------- ---------- ---------- ---------- --------- ---------- ----------
KAA0189413.1    ---------- ---------- ---------- ---------- ---------- ---------- --------- ---------- ----------
KAF7258038.1    ---------- ---------- ---------- ---------- ---------- ---------- --------- ---------- ----------
KAG5442206.1    ---------- ---------- ---------- ---------- ---------- ---------- --------- ---------- ----------
XP_030837801.1  ---------- ---------- ---------- ---------- ---------- ---------- --------- ---------- ----------
XP_041482585.1  ---------- ---------- ---------- ---------- ---------- ---------- --------- ---------- ----------
KRZ73557.1      ---------- ---------- ---------- ---------- ---------- ---------- --------- ---------- ----------
OUC42378.1      ---------- ---------- ---------- ---------- ---------- ---------- --------- ---------- ----------
CDW52968.1      ---------- ---------- ---------- ---------- ---------- ---------- --------- ---------- ----------
KHJ46144.1      ---------- ---------- ---------- ---------- ---------- ---------- --------- --MVTTLNIN ----FKNLKN
VZI00627.1      ---------- ---------- ---------- ---------- ---------- ---------- --------- ---------- ----------
XP_014681778.1  ---------- ---------- ---------- ---------- ---------- ---------- --------- ---------- ----------
VDM45293.1      ---------- ---------- ---------- ---------- ---------- ---------- --------- ---------- ----------
KAF8385568.1    ---------- ---------- ---------- ---------- ---------- ---------- --------- ---------- ----------
PIO76965.1      ---------- ---------- ---------- ---------- ---------- ---------M VAFI----- GKRATNA-L- ----VETGSP
VDL66086.1      ---------- ---------- ---------- ---------- ---------- ---------- --------- ---------- ----------
CAD6194288.1    ---------- -MCGSVLGLR AQL------- --------GH CDRYLRKTPH KQFQEFQPQS LLYF----- KKRDFKYGK- ----EQVQSM
XP_045095542.1  ---------- ---------- ---------- ---------- ---------- ---------- --------- ---------- ----------
EGT43056.1      ---------- ---------- ---------- ---------- ---------- ---------- --------- ---------- ----------
CAB3400100.1    ---------- ---------- ---------- ---------- ---------- ---------- --------- ---------- ----------
KAF7639475.1    ---------- ---------- ---------- ---------- ---------- ---------- --------- ---------- ----------
CAD5226593.1    ---------- ---------- ---------- ---------- ---------- ---------- --------- ---------- ----------
XP_024510735.1  ---------- ---------- ---------- ---------- ---------- ---------- --------- ---------- ----------
VDN24694.1      ---------- ---------- ---------- ---------- ---------- ---------- --------- ---------- ----------
KAH7730640.1    ---------- ---------- ---------- ---------- ---------- ---------- --------- ---------- ----------
TMS38138.1      ---------- ---------- ---------- ---------- ---------- ---------- --------- ---------- ----------
XP_013290959.1  ---------- ---------- ---------- ---------- ---------- ---------- --------- ---------- ----------
RCN50005.1      ---------- ---------- ---------- ---------- ---------- ---------- --------- ---------- ----------
KJH42542.1      ---------- ---------- ---------- ---------- ---------- ---------- --------- ---------- ----------
VDM64099.1      ---------- ---------- ---------- ---------- ---------- ---------- --------- ---------- ----------
VDO63723.1      ---------- ---------- ---------- ---------- ---------- ---------- --------- ---------- ----------
VDO44040.1      ---------- ---------- ---------- ---------- ---------- ---------- --------- ---------- ----------
CAD6198090.1    ---------- ---------- ---------- ---------- ---------- ---------- --------- ---------- ----------
KAF8373880.1    ---------- ---------- ---------- ---------- ---------- ---------- --------- ---------- ----------
XP_002632400.2  ---------- ---------- ---------- ---------- ---------- ---------- --------- ---------- ----------
RDD39080.1      ---------- ---------- ---------- ---------- ---------- ---------- --------- ---------- ----------
XP_001632619.1  ---------- ---------- ---------- ---------- ---------- ---------- --------- ---------- ----------
XP_020895242.1  ---------- ---------- ---------- ---------- ---------- ---------- --------- ---------- ----------
XP_027059688.1  ---------- ---------- ---------- ---------- ---------- ---------- --------- ---------- ----------
XP_029213515.2  ---------- ---------- ---------- ---------- ---------- ---------- --------- ---------- ----------
GFO16554.1      ---------- ---------- ---------- ---------- ---------- ---------- --------- ---------- ----------
RUS89945.1      ---------- ---------- ---------- ---------- ---------- ---------- --------- ---------- ----------
GFR80859.1      ---------- ---------- ---------- ---------- ---------- ---------- --------- ---------- ----------
XP_005106142.1  ---------- ---------- ---------- ---------- ---------- ---------- --------- ---------- ----------
CAG5118574.1    ---------- ---------- ---------- ---------- ---------- ---------- --------- ---------- ----------
XP_013069769.1  ---------- ---------- ---------- ---------- ---------- ---------- --------- ---------- ----------
XP_013069770.1  ---------- ---------- ---------- ---------- ---------- ---------- --------- ---------- ----------
KAH9514813.1    ---------- ---------- ---------- ---------- ---------- ---------- --------- ---------- ----------
XP_033638319.1  ---------- ---------- ---------- ---------- ---------- ---------- --------- ---------- ----------
XP_022095223.1  ---------- ---------- ---------- ---------- ---------- ---------- --------- ---------- ----------
XP_038051194.1  ---------- ---------- ---------- ---------- ---------- ---------- --------- ---------- ----------
ACO15530.1      ---------- ---------- ---------- ---------- ---------- ---------- --------- ---------- ----------
XP_040566914.1  ---------- ---------- ---------- ---------- ---------- ---------- --------- ---------- ----------
VDP33652.1      ---------- ---------- ---------- ---------- ---------- ---------- --------- ---------- ----------
PAA53847.1      ---------- ---------- ---------- ---------- ---------- ---------- --------- ---------- ----------
RNA13068.1      ---------- ---------- ---------- ---------- ---------- ---------- --------- ---------- ----------
CAF0715750.1    ---------- ---------- ---------- ---------- ---------- ---------- --------- ---------- ----------
CAF0950609.1    ---------- ---------- ---------- ---------- ---------- ---------- --------- ---------- ----------
CAF1119542.1    ---------- ---------- ---------- ---------- ---------- ---------- --------- ---------- ----------
CAF1301217.1    ---------- ---------- ---------- ---------- ---------- ---------- --------- ---------- ----------
CAF3941029.1    ---------- ---------- ---------- ---------- ---------- ---------- --------- ---------- ----------
CAF0867361.1    ---------- ---------- ---------- ---------- ---------- ---------- --------- ---------- ----------
UJR16523.1      ---------- ---------- ---------- ---------- ---------- ---------- --------- ---------- ----------
CAF1340180.1    ---------- ---------- ---------- ---------- ---------- ---------- --------- ---------- ----------
CAF1025683.1    ---------- ---------- ---------- ---------- ---------- ---------- --------- ---------- ----------
ALS04255.1      ---------- ---------- ---------- ---------- ---------- ---------- --------- ---------- ----------
XP_023329171.1  ---------- ---------- ---------- ---------- ---------- ---------- --------- ---------- ----------
KAH3870454.1    ---------- ---------- ---------- ---------- ---------- ---------- --------- ---------- ----------
XP_045201158.1  ---------- ---------- ---------- ---------- ---------- ---------- --------- ---------- ----------
XP_009053308.1  ---------- ---------- ---------- ---------- ---------- ---------- --------- ---------- ----------
XP_033096127.1  ---------- ---------- ---------- ---------- ---------- ---------- --------- ---------- ----------
XP_013785798.1  ---------- ---------- ---------- ---------- ---------- ---------- --------- ---------- ----------
GAU97340.1      MPCSCRSADL LSN------- ---------- --GSSRAPST DEDSLDSSPD SGEK-----R KTLE----- RKESYNYCEC VLE-REESVA
OQV24824.1      MLCSAHELAC VDCLATLKLA SAAAQQEPHQ SVTSPRDPAG AESTPPNTPN DGES------ --------- ------ACRS HEA-LVDGTA
CAD5123161.1    ---------- ---------- ---------- ---------- ---------- ---------- --------- ---------- ----------
ELT97609.1      ---------- ---------- ---------- ---------- ---------- ---------- --------- ---------- ----------
XP_009013880.1  ---------- ---------- ---------- ---------- ---------- ---------- --------- ---------- ----------
KAI0233246.1    ---------- ---------- ---------- ---------- ---------- ---------- --------- ---------- ----------
XP_029642194.1  ---------- ---------- ---------- ---------- ---------- ---------- --------- ---------- ----------
NP_001171747.1  ---------- ---------- ---------- ---------- ---------- ---------- --------- ---------- ----------
CAH1781438.1    ---------- ---------- ---------- ---------- ---------- ---------- --------- ---------- ----------
XP_013382323.1  ---------- ---------- ---------- ---------- ---------- ---------- --------- ---------- ----------
XP_033759917.1  ---------- ---------- ---------- ---------- ---------- ---------- --------- ---------- ----------
XP_041373453.1  ---------- ---------- ---------- ---------- ---------- ---------- --------- ---------- ----------
XP_046548515.1  ---------- ---------- ---------- ---------- ---------- ---------- --------- ---------- ----------
XP_047034441.1  ---------- ---------- ---------- ---------- ---------- ---------- --------- ---------- ----------
CAB3245311.1    ---------- ---------- ---------- ---------- ---------- ---------- --------- ---------- ----------
XP_011549456.2  ---------- ---------- ---------- ---------- ---------- ---------- --------- ---------- ----------
XP_045761472.1  ---------- ---------- ---------- ---------- ---------- ---------- --------- ---------- ----------
XP_046968321.1  ---------- ---------- ---------- ---------- ---------- ---------- --------- ---------- ----------
XP_026323335.1  ---------- ---------- ---------- ---------- MYLLRISPPR NGNVHIDLQF FFVYQLSYA ISFANEGQRC QDK-AKHSHA
XP_045529909.1  ---------- ---------- ---------- ---------- ---------- ---------- --------- ---------- ----------
```

```
RVE42816.1      ---------- ---------- ---------- ---------- ---------- ---------- --------- ---------- ----------
KAF9415294.1    ---------- ---------- ---------- ---------- ---------- ---------- --------- ---------- ----------
XP_018897307.1  ---------- ---------- ---------- ---------- ---------- ---------- --------- ---------- ----------
XP_019763455.1  ---------- ---------- ---------- ---------- ---------- ---------- --------- ---------- ----------
XP_030749737.1  ---------- ---------- ---------- ---------- ---------- ---------- --------- ---------- ----------
KAF7270051.1    ---------- ---------- ---------- ---------- ---------- ---------- --------- ---------- ----------
XP_031343879.1  ---------- ---------- ---------- ---------- ---------- ---------- --------- ---------- ----------
XP_044743527.1  ---------- ---------- ---------- ---------- ---------- ---------- --------- ---------- ----------
XP_018319432.1  ---------- ---------- ---------- ---------- ---------- ---------- --------- ---------- ----------
XP_026466152.1  ---------- ---------- ---------- ---------- ---------- ---------- --------- ---------- ----------
XP_022913687.1  ---------- ---------- ---------- ---------- ---------- ---------- --------- ---------- ----------
KAF5298473.1    ---------- ---------- ---------- ---------- ---------- ---------- --------- ---------- ----------
VEN57246.1      ---------- ---------- ---------- ---------- ---------- ---------- --------- ---------- ----------
XP_045472083.1  ---------- ---------- ---------- ---------- ---------- ---------- --------- ---------- ----------
XP_017768645.1  ---------- ---------- ---------- ---------- ---------- ---------- --------- ---------- ----------
CAH1369701.1    ---------- ---------- ---------- ---------- ---------- ---------- --------- ---------- ----------
XP_028136465.1  ---------- ---------- ---------- ---------- ---------- ---------- --------- ---------- ----------
XP_023014099.1  ---------- ---------- ---------- ---------- ---------- ---------- --------- ---------- ----------
XP_019875064.1  ---------- ---------- ---------- ---------- ---------- ---------- --------- ---------- ----------
CAH0557060.1    ---------- ---------- ---------- ---------- ---------- ---------- --------- ---------- ----------
XP_037924690.1  ---------- ---------- ---------- ---------- ---------- ---------- --------- ---------- ----------
XP_043863257.1  ---------- ---------- ---------- ---------- ---------- ---------- --------- ---------- ----------
XP_039965629.1  ---------- ---------- ---------- ---------- ---------- ---------- --------- ---------- ----------
XP_001989827.1  ---------- ---------- ---------- ---------- ---------- ---------- --------- ---------- ----------
XP_037896985.1  ---------- ---------- ---------- ---------- ---------- ---------- --------- ---------- ----------
XP_046805044.1  ---------- ---------- ---------- ---------- ---------- ---------- --------- ---------- ----------
KAG5681791.1    ---------- ---------- ---------- ---------- ---------- ---------- --------- ---------- ----------
XP_031629647.1  ---------- ---------- ---------- ---------- ---------- ---------- --------- ---------- ----------
KAG4077403.1    ---------- ---------- ---------- ---------- ---------- ---------- --------- ---------- ----------
XP_039430644.1  ---------- ---------- ---------- ---------- ---------- ---------- --------- ---------- ----------
XP_040221405.1  ---------- ---------- ---------- ---------- ---------- ---------- --------- ---------- ----------
CAA9998239.1    ---------- ---------- ---------- ---------- ---------- ---------- --------- ---------- ----------
KAF6203636.1    ---------- ---------- ---------- ---------- ---------- ---------- --------- ---------- ----------
KAG6800307.1    ---------- ---------- ---------- ---------- ---------- ---------- --------- ---------- ----------
XP_014230599.1  ---------- ---------- ---------- ---------- ---------- ---------- --------- ---------- ----------
KAH0947068.1    ---------- ---------- ---------- ---------- ---------- ---------- --------- ---------- ----------
XP_043590797.1  ---------- ---------- ---------- ---------- ---------- ---------- --------- ---------- ----------
XP_034949379.1  ---------- ---------- ---------- ---------- ---------- ---------- --------- ---------- ----------
XP_012275169.1  ---------- ---------- ---------- ---------- ---------- ---------- --------- ---------- ----------
XP_043464528.1  ---------- ---------- ---------- ---------- ---------- ---------- --------- ---------- ----------
XP_014220045.1  ---------- ---------- ---------- ---------- ---------- ---------- --------- ---------- ----------
XP_011160605.3  ---------- ---------- ---------- ---------- ---------- ---------- --------- ---------- ----------
XP_046741789.1  ---------- ---------- ---------- ---------- ---------- ---------- --------- ---------- ----------
VVC24333.1      ---------- ---------- ---------- ---------- ---------- ---------- --------- ---------- ----------
KAF0770685.1    ---------- ---------- ---------- ---------- ---------- ---------- --------- ---------- ----------
XP_015365968.1  ---------- ---------- ---------- ---------- ---------- ---------- --------- ---------- ----------
XP_029341681.1  ---------- ---------- ---------- ---------- ---------- ---------- --------- ---------- ----------
XP_026292519.1  ---------- ---------- ---------- ---------- ---------- ---------- --------- ---------- ----------
XP_034238427.1  ---------- ---------- ---------- ---------- ---------- ---------- --------- ---------- ----------
XP_024081347.1  ---------- ---------- ---------- ---------- ---------- ---------- --------- ---------- ----------
XP_014283188.1  ---------- ---------- ---------- ---------- ---------- ---------- --------- ---------- ----------
RZF32743.1      ---------- ---------- ---------- ---------- ---------- ---------- --------- ---------- ----------
CAB3380083.1    ---------- ---------- ---------- ---------- ---------- ---------- --------- ---------- ----------
XP_046989067.1  ---------- ---------- ---------- ---------- ---------- ---------- --------- ---------- ----------
GFG38476.1      ---------- ---------- ---------- ---------- ---------- ---------- --------- ---------- ----------
CAD7446618.1    ---------- ---------- ---------- ---------- ---------- ---------- --------- ---------- ----------
KAG8226740.1    ---------- -MTPGPLGIE DGVPKIA--- ----PQTGVL EDDRTFHSPQ DGELAADPLL SSSLKLTLL R--SDSPLER PGN-YPLLTF
XP_046392794.1  ---------- ---------- ---------- ---------- ---------- ---------- --------- ---------- ----------
XP_046459477.1  ---------- ---------- ---------- ---------- ---------- ---------- --------- ---------- ----------
XP_043227363.1  ---------- ---------- ---------- ---------- ---------- ---------- --------- ---------- ----------
XP_018006429.1  ---------- ---------- ---------- ---------- ---------- ---------- --------- ---------- ----------
AWH57222.1      ---------- ---------- ---------- ---------- ---------- ---------- --------- ---------- ----------
XP_042865604.1  ---------- ---------- ---------- ---------- ---------- ---------- --------- ---------- ----------
XP_045119740.1  ---------- ---------- ---------- ---------- ---------- ---------- --------- ---------- ----------
XP_045613573.1  ---------- ---------- ---------- ---------- ---------- ---------- --------- ---------- ----------
XP_021963432.1  ---------- ---------- ---------- ---------- ---------- ---------- --------- ---------- ----------
CAG7730563.1    ---------- ---------- ---------- ---------- ---------- ---------- --------- ---------C EVILFVIIGP
```

```
                91
KAH8855123.1    ---------- ---------- --MNQILSRI L KLAH--FN -KPLY---LN QLKGFN---- E---KPNILA AVVTCVDSRV IPSTFLCSNV
KAH9593836.1    ---------- ---------- --MNQILLRI L KSHV---N -KQSF---LD QLKAFN---- E---KPNILA AVISCVDSRV LTSKLLCSNV
VDP71164.1      RWI----RTA DKTQPDSHLT AYMERLIKGA L AFTR--GG -QREF---IK HV---N---- A---GYTPAG VLLSCVDARV FPSKILGAQP
TPP65121.1      ---------- ---------- --MERLIKGA L AFTR--GG -RQDF---LK HV---K---- T---SYKPLG VLVSCVDARI YPSKTLGTNP
KAA0189413.1    ---------- ---------- --MERIIKGA L AFTR--GG -RQDF---LK HI---K---- T---NYKPLG ILLSCVDARV YPSKILGANP
KAF7258038.1    ---------- ---------- MAMEVLMKGA L AYSR--TG -RQAF---LN QL---K---- S---LQSPVA VMISCVDSRV FPSQFLSAEP
KAG5442206.1    ---------- ---------- --MELLLQRA L VFTQ--NG QRTQF---IN QL---K---- S---LPNVCA VVISCVDPRV SPTNFLCSQP
XP_030837801.1  ---------- ----MQNGGS RRVGRALVQV C RRFS--SK P--GV---TS QTRPFQ---V G---LKPPLA VLVTCMDGRL LPSRIFKAER
XP_041482585.1  ---------- ----MHIGRF RLMGRVLVQV S KRFS--SQ Q--SV---PS QSRPFQ---V G---QKPPLA VLVTCMDGRL LPSRIFKAEC
KRZ73557.1      ---------- ----LSNYFF TKMRKLLNGV V KYRE--IA R-SQV---LK RLKQVK---- E---DFHPLT ILFSCVDARL ITSRVMQLDI
OUC42378.1      ---------- ---------- --MRKLLNGV V KYRE--TA R-SQV---LK RLRQVK---- E---DFHPVT ILFSCVDARL ITSRVMQLDI
CDW52968.1      ---------- ---------- --MQRILRSI A QQSR--KT R-LES---IQ RLN--Q---- Q---SLHPQA ILFCCVDSRF VTSRFLGLDV
KHJ46144.1      QIL----API TLIRFVAQSA KRMQRILRSI A QQSS--KT R-FES---IQ RLN--E---- Q---SLHPQA ILFCCVDSRF VTSRFLGFDI
VZI00627.1      ---------- ---------- --MEKIINGI L KFRT--DK R-ASV---LE KLAFFK---- R---TKHPNA IVISCMDARV YPTRFTASES
XP_014681778.1  ---------- -------MTS KGISRIMQGV L RYQS--TR R-ADV---AR DVRALR---- ENPGHIHPEA LYITCMDSRL QPSMTTQTHV
VDM45293.1      ---------- ---------M SGFAKILKGV V KFRH--GP R-GPA---LK KLQDIK---- KH--GHHATA VLFACMDARM TPLSFTQTEA
KAF8385568.1    ---------- ---------- --MQRILRGV I QYRQ--TV R-KEL---VE QFKEIK---- D---NPQPKA LMFTCMDSRM LPTRFTQAKV
PIO76965.1      RTV----GIR RRCFFLHTLP VSMNRVLRGV V KFRQ--TL R-KEL---VK QFEEIS---- N---HPKPTA VMFTCMDSRM LPTHFTQSYV
VDL66086.1      ---------- ---------- --MNRILRGV V KFRQ--TI R-NDL---AK QFDEIS---- D---HPRPTA VMFTCMDSRM LPTRFTQSNV
CAD6194288.1    SHP----QLE ERSANGSKQG KNMDKILRGV V KYRQ--TV K-KDL---VA QFEEVR---- N---NPKPTA VMFTCMDSRM LPTRFTQSKV
XP_045095542.1  ---------- ---------- --MNRIIRGV I QYNQ--KI K-AGL---VK QFEHVS---- D---HPNPTA VMFTCMDSRM LPTRFTQSAV
EGT43056.1      ---------- ---------- --MNKILRGV I QFRN--TI R-KDL---VK QFEEVK---- N---NPSPTA VMFTCMDSRM LPTRFTQSQV
CAB3400100.1    ---------- ---------- --MNKILRGV I KFRN--TI R-VDL---IE QFNKIS---- D---HPDPTA VMFTCMDSRM LPTRFTQSQV
KAF7639475.1    ---------- --------MH ESIKRIFDGI L LFRK--FK R-PAM---VK EFERVR---- D---HPDPRA VLFTCMDSRL QPSEFMQARI
CAD5226593.1    ---------- ---------M TGLRNLFQGV L KYRA--TV R-EDL---VK QFERVR---- D---DPHPAS VLFTCMDSRI LPARFTQAQV
XP_024510735.1  ---------- ---------M KGLDKVLQGV L KFRK--TI R-KDL---VK QFEEVR---- D---NPQPTA LFFTCMDSRI LPSTVTQSNV
VDN24694.1      ---------- ---------- MAFACLFDIV I CLYQ---- ---------VK QFVNIR---- DT--HAHPGA VFFSCMDARM FPARITSTTV
KAH7730640.1    ---------- ---------- --MQKVLDGI L KFRK--TV R-ADL---VK QFERVR---- D---NPNPTA VFFTCMDSRI LPARFTQAQV
TMS38138.1      ---------- ---------M PGLHKVLNGI I RFRQ--TV R-ADL---VK QFERVR---- D---NPNPTA VFFTCMDSRM LPARFTQSQV
XP_013290959.1  ---------- ---------M PGLHKVLQGI I KFRQ--TA R-NEL---VK QFEHVR---- S---NPNPTA VFFSCMDSRM LPARFTSSQV
RCN50005.1      ---------- MESAPHSEKM PGLHKVLQGI V KFRQ--TA R-KEL---VK QFEQIR---- N---NPHPTA VFFSCMDSRM LPARFTSSQV
KJH42542.1      ---------- ---------M PGLHKVLEGI I RFRK--TV R-KDM---VK QFEQIR---- N---NPNPTA VFFTCMDSRM LPARFTSSEV
VDM64099.1      ---------- ---------M PGLNKVLAGI I RFRK--TF R-KDM---VK QFEQIR---- D---NPNPTA VFFTCMDSRM LPARFTSSQV
VDO63723.1      ---------- ---------M PGMQKILAGI I RFRQ--TV R-KDL---VK QFEKIR---- N---NPAPTA VFFTCMDSRM LPARFTTSQV
VDO44040.1      ---------- ---------M PGMQKILSGV I RFRE--TV R-ADL---VR QFEKIR---- D---NPNPTA AFFTCMDSRM LPARFTSCNV
CAD6198090.1    ---------- ---------M PGIHKIINGV I RFRQ--TV R-QQL---VT QFERIR---- D---NPNPTA VFFTCMDSRM LPARITTSQV
KAF8373880.1    ---------- --------SM PGLNKILNGV I RFRQ--TV R-KDL---VK QFEKIR---- D---NPHPTA VFFTCMDSRM LPARFTQSQV
XP_002632400.2  ---------- ---------M PGLQKILNGV I RFRQ--TV R-KDL---VK QFEQVR---- D---NPHPTA VFFTCMDSRM LPARITSSQV
RDD39080.1      ---------- ---------- -MIEKVIRGV L RYSA--SL S-SAN---KT LYAQVA---- E---KVQPSC IFITCMDSRV FPSNIASIAP
XP_001632619.1  ---------- ---------- --MEKILQGV V RFRH--VL R-PSL---LP SLREVA---- E---KVAPKT VLVACVDCRI MPETYMSSEP
XP_020895242.1  ---------- ---------- --MDKVLRGI V NFTN--RV R-SNL---LP TLKDVA---- I---KAQPDM LMITCVDSRL LPGTYMSAVP
XP_027059688.1  ---------- ---------- --MEKLLQGV L KFKS--LV R-PSL---LP QLEKLA---- H---KAQPRM LLITCMDSRL CPTSFTQADP
XP_029213515.2  ---------- ---------- --MDKFLNGV V KFRK--LV K-PSI---LP LLQKLA---- N---SAQPRV LLITCVDSRL CPTSFTQADP
GFO16554.1      ---------- ---------- --MDKIIRGV L KFNS--RM R-KDY---TA DIDRFS---Q K---VVKPLT VFITCMDSRL NPITFVQANP
RUS89945.1      ---------- ---------- --MNKLLRGI V TFNS--KN R-KNY---LA GIQKVE---E Q---IVKPMA VFITCMDCRL DPPQFLQAVP
GFR80859.1      ---------- ---------- --MNKLLRGV L KFNS--AI R-KNY---LA EIDKVS---Q E---IVKPMT VFITCMDCRL DPLRFLQTQA
XP_005106142.1  ---------- ---------- --MEKMLRNI L KYNG--AG K-AKF---LA DMDNLA---S N---IIKPTT VFFSCIDSRV LAANFTRPDS
CAG5118574.1    ---------- ---------- --MDKILRGV V KYNL--HD K-VRF---VN DIDKFT---K K---IVQPQT VFISCMDSRV LPSKFVGSAI
XP_013069769.1  ---------- ---------- --MNVILKGI L KHNKELVY K-RRL---ID DIDKIT---K H---QVKAQT VCISCMDCRV LPSKFVNTEV
XP_013069770.1  ---------- ---------- --MNKILKGV I RYNKNITE K-AKL---VA DIDKVT---R Q---MVQPKS VFVSCMDCRV LPSKFLNSKI
KAH9514813.1    ---------- ---------- --MNKILKGV I RYNKNIAK K-SKL---VA DIDKVT---R Q---MVQPKS VFVSCMDCRV LPSKFLNSRI
XP_033638319.1  ---------- ---------- --MEKILRGI L KYKA--NA Q-GNF---LV RWANKA--EK L---PVKPPI LFVSCIDSRI LPTHFCQTSP
XP_022095223.1  ---------- ---------- --MEKILRGI I KYKA--NT Q-GNF---LV QWATLA--QK Q---HVKPPI LFISCIDSRV LPTHFCQTSP
XP_038051194.1  ---------- ---------- --MEKILRGI I KYKA--S- ---NF---LV RWFTHA--QK Q---HVKPPI LFVSCVDSRV LPTHFCQTSP
ACO15530.1      ---------- ---------- --MDKVLRGI L QYNR--SA KKKDV---LK QLSKIV---D S---QSTPSS VLFTCMDSRI HPNVFMNSDI
XP_040566914.1  ---------- ---------- --MEKVFRGI I RYKN--A- YKDDV---FT KLSKIK---E S---GSSPST VLFTCMDARI HPNVIMNSNV
VDP33652.1      ---------- -------MST KAIQKLLRGV L TYRS--KE K-AAL---LQ KLKEIQ---- K---NPNPLA VVVSCMDGRV LNRKLMQSDV
PAA53847.1      ---------- ---------- MGVERILRGV L NYRH--SV Q-KLF---QY ELRDIK---- S---HPNPSA VFISCMDARL FPNDFTLTKA
RNA13068.1      ---------- -----MIGKC SAMQTVLNGF L RYRA--NL R-GEL---LE KFKVAS---- V---DLNPQA VMVTCVDSRI LASRITQANP
CAF0715750.1    ---------- -----MITKC SGMQHILNGF L RYRA--KL R-GEL---IE KFKIAT---- V---NLNPQA ILITCVDSRI VASRITQANP
CAF0950609.1    --N----FSL KKIKEMPALH KGMGKILEGI L KYQK--TI Q-SEL---LP LFREIL---- D---KPAPKM AIVTGTDSRI VASRLLQAQP
CAF1119542.1    ---------- --MVASNIAR KGMGRILDGI I KYRQ--TL R-PAL---LE EFQKVA---- T---GPSPEG LLLTCVDSRV VASRITQAVP
CAF1301217.1    ---------- --MASSFSLR KGMSRILDGI M KYRQ--TL R-PLL---LE EFQKVA---- T---GPSPEG LLLTCVDSRV VASRITQAVP
CAF3941029.1    ---------- --MASSFATR KGMSRILDGI V KYRQ--TL R-PSL---LE EFKKVA---- T---GPSPEG LLLTCVDSRV VASRMIQAVP
CAF0867361.1    ---------- --MSTVHAVR KGMNRILDGI M QYRQ--AL R-PTL---LE EFKKVA---- M---GPSPEG LLLTCVDSRV VASRLTQAVP
UJR16523.1      ---------- --MSMSYAVR KGMNRILDGI M QYRQ--TL R-PTL---LE EFKKVA---- L---GPSPEG LLLTCVDSRV VASRLTQAVP
CAF1340180.1    ---------- --MAAGSSIR KGMTRILDGI M KYRH--AL R-PSL---LE EFQKVA---- L---GPSPEG LLLTCVDSRV VASRLTQAVP
CAF1025683.1    ---------- --MASAYSIR KGMARVLEGI M KYRQ--TL R-PSL---LE EFKKVA---- L---GPSPEG LLLTCVDSRV VASRLTQAVP
ALS04255.1      ---------- -----MAGVN HGMEKIFRGI M KYRL--NH Q-RNM---VE QFKQVR---- D---NPVPTA VFFTCVDSRM LPTRFTQTQV
XP_023329171.1  ---------- -----MAEAR HGMEKIFRGL M TYRL--TH Q-RNM---VD QFKKVR---- D---NPVPTA VFFTCVDSRM LPSRFTQTQV
KAH3870454.1    ---------- ---------M RGFSKLLEGI V KYNL--FD K-KHT---LF QLKLYG---- E---NPDPLS LMFSCMDSRV VSTKFTNSDL
XP_045201158.1  ---------- ---------M RGFSKLLEGI V KYSL--FD K-QHT---MF QLKIYG---- N---KPNPLS MMFACMDSRV IPSKYTNSDL
XP_009053308.1  ---------- ---------M PVIEKILQGI V RYNN--VT K-PAA---LK QLIEVN---- K---NVQPKA IVISCIDARV LPAKFLDNEI
XP_033096127.1  ---------- ---------- MGMQKILRGV M QYRA--TV K-PGI---VK QLKAVS---- D---HPKPKA LVFSCMDSRM LPTRFTQTDI
XP_013785798.1  ---------- ---------- MGLEKILRGI M KYRK--SS CHSKM---LE KYQHIKEMYN S---SPRPSA IFFTCMDSRI SPSQFTQTEV
GAU97340.1      GPP----GFQ TRKVRDEKMV FGIDKVLSGI V KYHQ--TV R-GGM---LE QFKRVR---- D---NPVPSV LMFTCMDSRM IPSRFTQSDV
OQV24824.1      VPL----PTS PNGKRDASMV FGIDKVLSGI V KYHQ--TV R-NGM---VE QFRRVR---- D---NPVPSV LMFTCMDSRM IPSRFTQSDV
CAD5123161.1    ---------- ---------M SVLGNLLKGV L KFSK--NV Q-PSL---VK RLAELQ---- K---QPQTKA VMFSCMDSRV LTSDIMQTEP
ELT97609.1      ---------- ---------M SGIGKLLRGC L EFNA--LK K-PKL---LE QFKAVK---- E---HPAPKA VTFSCMDNRI IITKLLQCDV
XP_009013880.1  ---------- ---------M PGLNKLLRGI L KYRT--NL Q-PHV---LK QFQRVK---- D---NPQPSS VMFSCMDSRL IITKMINQDV
KAI0233246.1    ---------- ---------M PGLDKLLKGV L KYRE--TI R-TQM---VK DFQRIR---- E---KPEPKA VMVSCIDSRV IMTRLLQSNV
XP_029642194.1  ---------- ---------M KGLEKILRGI I KYQL--TD K-VRM---KL SPQNII---- E---YPTPNF LFISCIDNRV LPSHFTKIDC
NP_001171747.1  ---------- ---------M PGMEKLLRGV L RYRN--GV R-QEL---IP QFERVR---- D---NPEPTA ILFTCMDSRM LPTRFCQTNV
CAH1781438.1    ---------- ---------M PGLQKVLKGV L QYRE--LF K-SKM---VE KLVQIS---- E---HPQPTA LLFTCMDTRL QPSVITMSNP
XP_013382323.1  ---------- ---------M PGIDKLLKGV L KYRS--TI K-DEM---TQ KLKEVT---- V---NPQPTA VVFSCMDARV LPTRFCQTDV
XP_033759917.1  ---------- ---------M PGIEKLLLGI L RYRV--NT R-WKL---LE QFQRVK---- D---NPEPTS VFFTCIDSRV LATKFMDSQV
XP_041373453.1  ---------- ---------M PGISHMLKGI L RYKK--ID Q-VHL---VE QFKQVR---- E---NPEPLS VFFTCIDSRV LASRFTNSQV
XP_046548515.1  ---------- ---------M PGIDKIIKGI L HFRR--FD R-PFM---VE EFLRVK---- A---DPQPSS VFFTCMDSRV LASRFTTSHV
XP_047034441.1  ---------- ---------- --MEHNNNGY - -------- L-CIF---QE EYVPGP---- L---VSLPKA VFYTCMDSRM IPTRFTETSV
CAB3245311.1    ---------- ---------- --MEKILRGI M RYRV--LD R-ASM---VK QFQEVK---- N---NPTPKA VFYTCMDSRM IPTRFTETSV
XP_011549456.2  ---------- ---------- --MERILRGI M RYRV--LD R-ATM---VK QFEQVQ---- D---HPVPKA VFYTCMDSRM IPTRFTETSV
XP_045761472.1  ---------- ---------- --MDRILRGI M RYRI--LD R-ATM---VK QFQEVK---- D---NPVPKA IFYTCMDSRM IPTRFTETCV
XP_046968321.1  ---------- ---------- --MDRILRGI M RYRV--LD R-ASM---VK QFEQVR---- D---NPVPKA IFYSCMDSRM IPTRFTETSV
XP_026323335.1  FFGALLHLII SKKHLCVTNK TNMDRILRGI M RYRV--LD R-ASM---VK QFQQVK---- D---NPVPKA IFYTCMDSRM IPTRFTETSV
XP_045529909.1  ---------- ---------- --MDRILRGI M RYRV--LD R-ASM---VK QFEQVK---- D---NPVPKA IFYTCMDSRM IPTRFTETSV
RVE42816.1      ---------- ---------- --MDRILRGI M RYRV--LD R-ASM---VK QFQAVK---- D---NPTPKA VFYTCMDSRM IPTRFTETSV
KAF9415294.1    ---------- ---------- --MEKILRGI M RYRV--LD R-ASM---VK QFQEVK---- N---NPTPKA VFYTCMDSRM IPTRFTETSV
XP_018897307.1  ---------- ---------- ---------- M KHQN--TS L-FQVHFRSI LLDHIR---- C---IKQPKA VFFTCMDSRM IPTRFTETNV
```

```
XP_019763455.1  ---------- ---------- --MDKLLKGI M KYRS--TV K-DTM---VQ QFVKVK---- D---NPTPKA VFFTCIDSRM LPTRFTQTNV
XP_030749737.1  ---------- ---------- --MDKLLKGI M KYRS--TV R-DTM---VK QFLKVK---- D---NPTPKA VFFTCIDSRM LPTRFTQTNV
KAF7270051.1    ---------- ---------- ---------- M QYRA--TV R-DTM---VQ QFLKVK---- D---NPTPKA VFFTCIDSRM IPTRFTQTNV
XP_031343879.1  ---------- ---------- --MDRILRGV M KYRH--ID K-AKM---LQ QFLQVR---- D---TPTPKA VFFTCMDSRM LPTRFTDMNI
XP_044743527.1  ---------- ---------- ---------- M KYRH--LD L-KTM---VQ QFEQVR---- D---NPIPKA VFFTCMDSRM IPTRFTETNV
XP_018319432.1  ---------- ---------- --MDRILRGI M RYRQ--LD R-KTM---VE QFKQVK---- N---NPTPKA VFFTCMDSRM IPTRFTEMNV
XP_026466152.1  ---------- ---------- --MDRILKGI M KYRE--VT K-KTM---VK QFECVK---- D---HPTPKA VFFTCMDSRM IPTRFTETNV
XP_022913687.1  ---------- ---------- --MDRILRGV M RYRN--VG K-EMM---LK QFQEVR---- D---NPTPKA VFFTCMDSRM IPTRFTQTNV
KAF5298473.1    ---------- ---------- --MDRILRGI M KYRN--TD K-VKM---VQ QFLQVR---- D---NPTPKA VFFTCIDSRM IPTRFTEMNV
VEN57246.1      ---------- ---------- --MDKILKGI M RYRA--NK R-EIM---VK QFLEVK---- D---NPKPKA VFFSCIDSRM IPTRFTQMNV
XP_045472083.1  ---------- ---------- --MNRILRGI M RYRE--IK K-ERM---VQ QFLKVK---- D---NPMPKA VFFTCIDSRM IPTRFTQTNV
XP_017768645.1  ---------- ---------- --MDRILKGI M KYRQ--LG K-KTM---VE QFKEVK---- D---NPIPKA VFFTCMDSRM IPTRFTETNV
CAH1369701.1    ---------- ---------- --MDRILRGI M RYRS--VK K-EQM---VK QFVQVK---- D---NPTPKA VFFTCIDSRM IPTRFTQTNV
XP_028136465.1  ---------- ---------- --MDKLLKGI I KYRG--TA R-DEM---VK QFLQVK---- D---HPTPKA VFFTCIDSRM IPTRFTQTEV
XP_023014099.1  ---------- ---------- --MDKLLKGI M KYRS--NI K-ENM---VK QFVQVK---- D---NPKPKA VFFTCIDSRM LPTRFTQTNV
XP_019875064.1  ---------- ---------- --MDKLLKGI M RYRE--VI K-DKM---VQ QFVQVK---- D---NPTPKA VFFTCIDSRM IPTRFTQTNV
CAH0557060.1    ---------- ---------- --MDKLLKGI M RYRS--TK K-NKM---VE MFKRVK---- D---NPVPKA VFFTCIDSRM IPTRFTQTNI
XP_037924690.1  ---------- ---------- --MERILRGI M RYRN--SS R-IQM---VK EFQYVR---- D---NPEPKA VFFTCMDSRM IPTRFTETHV
XP_043863257.1  ---------- ---------- ---------- M CSRA--HD A-KIR---RE GGDTFK---- C---KNKPKA VFFTCMDSRM IPTRYTDTHV
XP_039965629.1  ---------- ---------- --MERILRGI M RYRN--TT R-EQM---VK EFQKVR---- D---HPEPKA VFFTCMDSRM IPTRYTDTHV
XP_001989827.1  ---------- ---------- --MERILRGV M RYRN--TT R-EQM---VK EFQKVR---- D---NPEPKA VFFTCMDSRM IPTRYTDTHV
XP_037896985.1  ---------- ---------- --MERILRGI M RYRN--TT R-EQM---VK EFQRVR---- D---NPEPKA VFFTCMDSRM IPTRYTDTHV
XP_046805044.1  ---------- ---------- --MERILRGI M RYRN--TT R-EQM---VR EFQKVR---- D---HPEPKA VFFTCMDSRM IPTRYTDTHV
KAG5681791.1    ---------- ---------- --MERILRGI M RYRN--TT R-AAM---VK EFEQVR---- D---NPQPKA FFFTCMDSRM IPTRFTDTHV
XP_031629647.1  ---------- ---------- --MDRIIRGI M RYRH--TT R-EQM---VQ EFIKVK---- N---DPHPKA VFFTCMDSRM IPTRFTETHV
KAG4077403.1    ---------- ---------- --MERILRGI M RYRH--TT R-EQM---VK EFQKVR---- D---NPQPKA VFFTCMDSRM IPTRFTETHV
XP_039430644.1  ---------- ---------- --MDRILRGV M RYRN--TT R-EQM---VK EFQKVR---- D---NPQPKA VFFTCMDSRM IPTRYTDTHV
XP_040221405.1  ---------- ---------- --MERILRGV M RYRH--TT R-EQM---VQ EFRKVR---- D---NPQPKA VFFTCMDSRM IPTRFTETHV
CAA9998239.1    ---------- ---------- --MSMCLSLI M IH------ ---------- KLFNKV---- F---DKHPKA VFFTCMDSRM IPSRFTQTNV
KAF6203636.1    ---------- ---------- --MDRLFRGI M RYRN--TE R-RGM---VA QFEKVK---- N---NPVPKA VFFTCMDSRM IPSRFTQTNV
KAG6800307.1    ---------- ---------- --MDKIIKGI M KYRK--CH R-EEM---VK QFQKVK---- D---CPEPKA AFFTCMDSRM IPTRFTETNV
XP_014230599.1  ---------- ---------- --MDRIIKGV M KYRK--CH R-EGM---VK QFQQVR---- D---NPQPKA VFFTCMDSRM IPTRFTETNV
KAH0947068.1    ---------- ---------- --MDKILKGI M KYRK--CH R-KGM---VK QFQQVR---- D---HPEPKA LFFTCMDSRM IPTRFTETDI
XP_043590797.1  ---------- ---------- --MDRIIKGI M KYRN--CH K-KEL---VK QFQKVR---- D---HPDPKA VFFTCMDSRM IPTRFTETNV
XP_034949379.1  ---------- ---------- --MDRIIRGI M KYRQ--CH R-EGM---VK QFQQVR---- D---NPEPKA VFFTCMDSRM IPTRFTETNV
XP_012275169.1  ---------- ---------- --MDRILKGV M KYRK--CH K-EGM---VK QFEQVK---- L---HLEPKA VFFTCMDSRM IPTRFTETNV
XP_043464528.1  ---------- ---------- --MDRILRGI M KYRQ--CH R-EGM---VK QFEQVR---- D---NPEPKA IFFTCMDSRM IPTRFTETNV
XP_014220045.1  ---------- ---------- --MDRILKGV M RYRK--CH R-DVM---VK QFQQVK---- D---HPEPQA VFFTCMDSRM IPTRFTETNV
XP_011160605.3  ---------M TVLLHSYCSN AVMDRILKGI M KYRK--CH R-EGM---VK QFQQVR---- D---HPEPKA VFFTCMDSRM IPTRFTETNV
XP_046741789.1  ---------- ---------- --MDRILKGV M KYRK--CH R-EGM---VK QFKQVK---- D---HPEPKA VFFTCMDSRM IPTRFTETNV
VVC24333.1      ---------- ---------- --MDRIFRGI M KYRQ--TN R-KKM---VE QFVHVK---- N---HPEPKA LFFTCMDSRM LPARFTETNV
KAF0770685.1    ---------- ---------- --MDRIFRGI M KYRQ--TH R-VEM---VE QFVKVK---- N---RPEPKA LFFTCMDSRM LPAKFTKSNV
XP_015365968.1  ---------- ---------- --MDRIFRGI M KYRR--TN R-VKM---VE QFIQVK---- N---RPEPKA LFFTCMDSRM LPARFTDSNV
XP_029341681.1  ---------- ---------- --MDRIFRGI M KYRR--TN R-GKM---VE QFVQVK---- N---HPEPKA LFFTCMDSRM LPARFTESNV
XP_026292519.1  ---------- ---------- ---------- M TFH----- ---RM---VC NHGKRS---- M---PCQPKA VFFTCMDSRM IPTRFTETNV
XP_034238427.1  ---------- ---------- --MDKILKGI M KYRV--TD R-KTM---VE QFKQVA---- N---NPAPKA VFFTCMDSRM IPTRFTETNV
XP_024081347.1  ---------- ---------- --MDRLFRGI M NYRG--TD R-LYM---LE QFARVR---- D---HPVPKA VFFTCMDSRM IPTRFTKTNV
XP_014283188.1  ---------- ---------- --MDRLLRGI I KYRN--TD K-HIM---LD QFVQVK---- N---NPTPKA VFFTCMDSRM IPTRFTKTNV
RZF32743.1      ---------- ---------- --MDRILRGV M RYRH--TD R-KTM---VE QFIQVK---- N---NPVPKA VFFTCMDSRM IPTRFTETNV
CAB3380083.1    ---------- ---------- --MDRIIRGI I KYHH--HT R-LGM---VE QFKRVR---- D---NPAPKA VFFTCMDSRM IPTRFTETNV
XP_046989067.1  ---------- ---------- --MDRILRGI M KYRA--TD R-KTM---VE QFQRVR---- D---NPEPKA VFFTCMDSRM IPTRFTETNI
GFG38476.1      ---------- ---------- --MDRILRGI M KYRQ--TD R-KKM---VE QFEVVR---- N---RPEPKA VFFTCMDSRM IPTRFTETNV
CAD7446618.1    ---------- ---------- --MDKILRGV M KYRH--TD R-KTM---VA QFERVR---- D---HPEPKA VFFTCMDSRM IPTRFTETNV
KAG8226740.1    LWATLFRCDN GGAKLPPHSI SDMDRIIRGI M KYRQ--TG R-KTM---VQ QFEQVR---- D---NPEPKA VFFTCMDSRM IPTRFTETNV
XP_046392794.1  ---------- ---------- --MDRILRGI M KYRV--TD R-KTM---VE QFVKVR---- D---NPEPKA VFFTCMDSRM IPTRFTETNV
XP_046459477.1  ---------- ---------- --MDKILKGI L KYRK--TY R-TEM---VE QFKQVA---- D---RPEPKA VFFTCMDSRM LPTRFTQTDV
XP_043227363.1  ---------- ---------- --MDKILKGI L KYRH--TF R-PGM---VE QFQQVK---- D---NPVPKA IFFTCMDSRM LPTRFTYTNV
XP_018006429.1  ---------- ---------- MGIDKILQGV M KYRQ--TC R-DGM---VQ QFKVVR---- N---DPHPKA VFFTCMDSRM LPTRFTQTNV
AWH57222.1      ---------- ---------- MGMSRILLGV M KYRQ--NY R-AGM---VK QFEVVR---- D---DPHPKA VFFTCMDSRM LPTRFTQTNV
XP_042865604.1  ---------- ---------- MGMSRILQGI M RYRE--SY R-AGM---VK QFEVVR---- D---DPHPKA VFFSCMDSRM LPTRFTQTNV
XP_045119740.1  ---------- ---------- MGMTRILQGI M RYRN--TY R-ERM---VK QFEVVR---- D---DPHPKA VFFTCIDSRM LPTRFTQTNV
XP_045613573.1  ---------- ---------- MGMHRILQGV M KYRR--TK R-EGM---VK QFEVVR---- D---DPHPKA VFFTCIDSRM LPTRFTQTNV
XP_021963432.1  ---------- ---------- MGMDRILKGI M QYRQ--TV R-SNL---VQ EFQRVR---- D---HPEPKA VFFTCMDSRM LPTRFTQTHV
CAG7730563.1    FWP------- --VYGVAEVT TEMFRIIHGV M KYRE--AI R-QGM---VQ EFSAVR---- D---HPDPKA VFFTCMDSRM IPTRFTQTHV
```

```
                      181
KAH8855123.1          GE LFIKRNP GNFVCCENTS L--------- ------NHSN ENYVTPGFLE LTLIRCKITD III CGHSDC RAMNLLNNLG KCMHEQQYPY
KAH9593836.1          GE LFIERNP GNFICCENSS L--------- ------EHFN KNCVTPGFLE LTLIRCRIND III CGHSDC RAMNLLNNLG KCKYERSHPY
VDP71164.1            GE LLILRNA GNFAPEHGEG ---------- ---------- GSNTVLSTLE LGCLHGKVKD VVI CGHSDC KAMHLLCSIG SKVTD-----
TPP65121.1            GE LYVIRNA GNFAPEDGAP ---------- ---------- AAGTVLSTLE LGCLRGSVKD VII CGHSDC KAMHLLRSIG PELDN-----
KAA0189413.1          GE LYVVRNA GNFVPENGAG ---------- ---------- EAGTILSTLE LGCLRGSVKD III CGHSDC KAMHLLQSFG SEAEN-----
KAF7258038.1          GQ LYIVRNA GNFVPQANSS ---------- ---------D KECNVLGTLE LACIRNKVND IVI CGHSDC KAMHLLNSMG PSIMN-----
KAG5442206.1          GR MFTIRNA GNFVPCGEPH S--------- ---------E SSCTVLGTLD LACIRGKANE LIV CGHSDC KAMHLLNSIG PSLVR-----
XP_030837801.1        GE LLIIRNP GNFVPHSCKC E--------- -PSEGSEAPA FPSGELAGLQ LAIQKMAIPD VIV CGHTDC RAGEALRHLP VSRPTGQ---
XP_041482585.1        GE LLIIRNP GNFVPHSCKC E--------- -SSAESTAPA FPSGEMAGLQ LAIQKMAIPD VIV CGHTDC RAGEALRNLP VSRPTGH---
KRZ73557.1            GD AYMVKNP GNMIPCTYTC A---KL---- ------QQNA ASLSALASIE LACLMKNVKD IVV CGHSDC SAMNLLRSME QCEAE-----
OUC42378.1            GD AYMVKNP GNMIPCTYTC A---KL---- ------QQNA AGLSALASIE LACLMKNVKD IVV CGHSDC SAMNLLHSMK QCEAE-----
CDW52968.1            GD AYTVRNP GNIIPFCFPG G---SVANLL IFLYGTKQPS AITEATASLQ LACCESKVPE VIV CGHSDC KAMKLLWSLR NEKHE-----
KHJ46144.1            GD AYTVRNP GNIIPFCDSD G--------- -----TKQAG AIAEATASLK LACCESKVPE VIV CGHSDC KAMKLLWLLR TEKHQ-----
VZI00627.1            GD LYTVRNA GNFIPHAQEF A--------- ------DRKL PISNEPGALE LACCRSGVKD III CGHSDC KAMNLLMKVG EDSAIN----
XP_014681778.1        GD MYVIRNS GNLVPHSDHC G--------- -----EDLWK HPSCETAALD LCCVRSNVGN VIV TGHSDC KAMNALYDVV STVAA-----
VDM45293.1            GD MYIVRNG GNMIPSATHF G--------- ----ACGDEM LVATEPAALD LTLKQGGLKH AIV CGHSNC KAMNALYQMH LHPKK-----
KAF8385568.1          GD IFVVRNA GNLIPDACNY G--------- -----HYSEV SCTTEPAALE LAVKRGGVKH VIV CGHSDC KAMNMLFGLH ACPSN-----
PIO76965.1            GD MFVVRNA GNMIPDAPHY G--------- -----TSSEI SITTEPAALE LAVTRGGIRH IIV CGHSDC KAMNLLHSLH QCPKN-----
VDL66086.1            GD MFVVRNA GNMIPDAPRY G--------- -----VSSEI SVTTEPAALE LAVKRGGIRH IVV CGHSDC KAMNLLHSLH QCCTN-----
CAD6194288.1          GD MFVVRNA GNMIPEAPNY G--------- -----FSSEV SVTTEPAALE LAVKRGGINH IVV CGHSDC KAINTLYGLH QCPKN-----
XP_045095542.1        GD MFVVRNA GNMIPAAPNY G--------- -----SYSEV SINTEPAALE LAVKRGKIRH VVV CGHSDC KAMNTLYQLH QCPTK-----
EGT43056.1            GD MFVVRNA GNMIPDAPNY G--------- -----AFSEV SVNTEPAALE LAVKRGGIRH IVV CGHSDC KAINTLYGLH QCPKN-----
CAB3400100.1          GD MFVVRNA GNMVPDAPNY G--------- -----RFSEV SVNTEPAALE LAVKRGGIRH VVV CGHSDC KAINTLYGLH QCPKN-----
KAF7639475.1          GD LFVVRNS ANMVPLARNF G--------- ----GTASEV SVTTEPAALE LAIKRGNVCQ VLV CGHSDC KAINTLYQLY NGR-A-----
CAD5226593.1          GQ MFVVRNS GNMIPHAQNY G--------- ----GSSYEV SITTEPAALE LAVKRGNIHH VLV MGHSDC KAINMLHNLC QCPQH-----
XP_024510735.1        GD MFVVRNS GNMIPGSRSF G--------- ----NVGHEV SVNTEPAALE LAVTRGKINH VIV CGHSDC KAINTLYNLH TGVNK-----
VDN24694.1            GD MYVVRNP GSMIPRAENY G--------- ----SCGCET SLSTEAAGLE LTVKRGGIKH VII CGHSNC KAMNTLYNLH KNPKK-----
KAH7730640.1          GD MFVVRNS GNMVPHAQNY G--------- ----ISGYEV SVTTEPAALE LAVKRGKIKH VIV CGHSDC KAINTLYNLH TCTSA-----
TMS38138.1            GD MFVVRNS GNMVPHAQNY G--------- ----MSGFEV SVTTEPAALE LAVKRGGIKH VIV CGHADC KAINTLYNLH QCPKH-----
XP_013290959.1        GD MFVVRNS GNMIPHANNY EGMYRD---- -VSGPAGFEV SVTTEPAALE LAVKRGHINH VIV CGHSDC KAINTLYNIH TCPQN-----
RCN50005.1            GD MFVVRNS GNMIPHANNY G--------- ----PAGYEV SVTTEPAALE LAVKRGHINH VIV CGHSDC KAINTLYNIH ECPHT-----
KJH42542.1            GD MFVVRNS GNMIPHANNY G--------- ----PTGYEV SVTTEPAALE LAVKRGHINH VIV CGHSDC KAINTLYNIH KCPKS-----
VDM64099.1            GD MFVVRNS GNMIPHANNY GKMLNTSTSV AYVGSSGYEV SVTTEPAALE LAVKRGHINN VIV CGHSDC KAINTLYNIH RSPKS-----
VDO63723.1            GD MFVVRNS GNMVPHAEHY GLIHGIWLAC VFLGPAGYEV SVTTEPAALE LAVKRGHIHH VIV CGHSDC KAINTLYNLH KCPKT-----
VDO44040.1            GD MFVVRNS GNMVPHAAHY G--------- ----PSGYEV SVTTEPAALE LAVKRGHINH VIV CGHADC KAINTLYNLH KNPKC-----
CAD6198090.1          GD MFIVRNS GNMIPHAKNY G--------- ----PAGFEV SVTTEPAALE LAVKRGKIKH VIV CGHSDC KAINTLYNLH TCPGA-----
KAF8373880.1          GD MFVVRNS GNMIPHANNY G--------- ----PVGYEV SVTTEPAALE LAVKRGKINH VIV CGHSDC KAINTLYNLH CCPKS-----
XP_002632400.2        GD MFVVRNS GNMIPHANNY G--------- ----PSGYEV SVTTEPAALE LAVKRGKINH VIV CGHSDC KAINTLYNLH KCPKS-----
RDD39080.1            GE SFIVRNA GNIVPHSKLI Y--------- ------ER-- WTPAEAAALE LACVRNQVSS VVV CGHSDC KAMDGLHSLG GTAPS-----
XP_001632619.1        GD MFVVRTA GNLLPHAKLY G--------- ------DV-- GSCSELAALQ MAIQEGKVEN VVV CGHSNC KGMTFLLSHD SR--------
XP_020895242.1        GQ MFVVRNV GNLFPHARLF G--------- ------SQV- ASSAEPAALE LAVINYGIKH VAV CGHSDC KAMHALYDTA SSSST-----
XP_027059688.1        GD MFIMRNA GNVVPHNQLF G--------- ------ENQQ SPLSERAALE LAIS-TGVKD ITV CGHSDC KAMDCLYHMH NTTDKKK---
XP_029213515.2        GD MFIVRNA GNTMPHSQLF G--------- ------ESCS SALSERAALE LAVA-TGVEH IAV CGHSDC KAMACLHQSS STA-------
GFO16554.1            GE VFCIRNP GNMVPHYCEA E--------- ------A--E GISAEGAGLE LGCIMNDIGN VVV CGHSDC KAVNSLYAQR NCWHRIQ---
RUS89945.1            GE IFIIRNP GNMVPFFDPA G--------- ------Q--H TLSAEGGGLE LGCIMNGIGD VVV CGHSDC KAVNSLYTQR YDHAM-----
GFR80859.1            GE VFVVRNP GNMVPLFDPS K--------- ------P--D NASAEGGGLE LGCIMNGIGD VVV CGHSNC KAVNSLYSQR HVCHEAQ---
XP_005106142.1        GE TFFVQNA GNMVPHFQSI V--------- ------S--G QMSTEGAALE LGCLSKGIRS VVV CGHSDC KAVHALYDMR SVCDHT----
CAG5118574.1          GE TFYVRNA GNMIPHHEDY L--------- ------A--G YISSEGGGLE LGCIVNEIRN VVI CGHSDC RALYALYDMR KECDHK----
XP_013069769.1        GQ IYFLRNA GNIVPHFDDF K--------- ------E--G RISSEGAGLE LGCVTNQVKN VVV LGHSDC RAAQALFEIR HSCNHE----
XP_013069770.1        GE IYFLRNA GNMVPHFDAY K--------- ------S--G KISSEGGGLE LGCITNKIRN VVV CGHSDC KAAQALFEIR HNCDHQ----
KAH9514813.1          GE IYFLRNA GNMVPHFDDY K--------- ------S--G KISSEGGGLE LGCIRNKIRN VVV CGHSDC KAAKALFEIR DTCDHH----
XP_033638319.1        GD MFILRNA GNVLPRANYS E--------- ------GEPT QLSCEVVALE ITCNRSKVEH VVI CGHSDC QALYAAYKHY TEEEETS---
XP_022095223.1        GD MFLLRNA GNVIPQANYT Q--------- ------GEYT QSSSEVVALE ITCNRSKVEH VVV CGHSDC QALYAAYSHY RERE-GK---
XP_038051194.1        GD MFILRNA GNVIPRANYS E--------- ------GEPT HVSCEVVALE ITCNRSKVEH VVV CGHSDC QALYAAYSHY TEKE-TK---
ACO15530.1            GD AFTVRNA GNIVPKSGLV H--------- ------GL-V NPAPEPAGLE LGCVLNSIKN VIV CGHSDC KAMIAVHSLK DSNGWSE---
XP_040566914.1        GD VFTVRNP GNIVPNASYV A--------- ------NS-R TPAPEPAGLE LGCVVNSIKN V-V CGHSDC KAMIALQSFG DSKGCSE---
VDP33652.1            GD LYLVKNP SNMVPAMRGN D--------- ------VN-Q AAVAAVAALQ MTTVLGGIRD IIV FGHGNC SAISAMYQLF KENKP-----
PAA53847.1            GD MYVMRNA GNMAPPDELG R--------- ---------- ---GATAAGVE LGCIHNDIKD VII CGHSDC KAMALLWKLR DTIDT-----
RNA13068.1            GE ILITRNP GNLVPNYNVL P--------- ------A--K TSRPEEAALE LACLHDHVDT IVV AGHADC KAMNLVHDNR DQLFKPA---
CAF0715750.1          GE ILITRNP GSLVPNYNVL P--------- ------P--N TSRPEEAALE LACLHDNIDT VAI AGHADC KAMNLVHDNR HQIFKSP---
CAF0950609.1          GT FFLIRSP GNFIPKYESL D--------- ------T--S VASGTPAALE LACVNNNANT IVV LGHSDS KPLNMLFDMK DNLEEH----
CAF1119542.1          GQ LFIVRNP GNLVPHHNCF R--------- ------NN-V TVGGECGALE LACSINKVQV IVV IGHSDC KAMNLLYSIR NELKS-----
CAF1301217.1          GQ LFIVRNP GNLVPNHNCF R--------- ------NN-V TVGGECAALE LACSINKAQV IVV IGHSDC KAMNLLYSIR DDLKS-----
CAF3941029.1          GQ LFIVRNP GNLVPHHNCF R--------- ------NH-V TVGGECGALE LACSINKVQV IVV IGHSDC KAMNLLYSIR NDLQS-----
CAF0867361.1          GQ LFFVRNP GNLVPPYDYF E--------- ------KN-N IVGGECAALE LACSRNNVPV ITV FGHSDC KAMNLLYRIR NEISI-----
UJR16523.1            GQ LFIVRNP GNLVPSYDYF E--------- ------KN-G IVSGECAALE LACSRNNVPV IAV FGHSDC KAMNLLYRIR NEISI-----
CAF1340180.1          GQ LFFVRNP GNLVPPYEYY Q--------- ------NN-A LVGGECAALE LACSRNNVPV IAV FGHSDC KAMNLLYRIR NEIAI-----
CAF1025683.1          GQ LFIVRNP GNLVPPYEYY K--------- ------DN-S LVGSECAALE LACSRNNVPM IAV FGHSDC KAMNLLYKIR NEIST-----
ALS04255.1            GD MFIVRNA GNMVPHSSKV S--------- ------D--T NVATEPAVLE LACVMNTVKH VVV CGHSDC KAINLLYNLY SNPDTQD---
XP_023329171.1        GD MFIVRNA GNMVPHSSKV S--------- ------P--S AVATEPAVLE LACVINTVKH VVV CGHSDC KAINLLYSLH TDPKTQN---
KAH3870454.1          GD NYIVKNA GNFVPYPDDL E--------- ------QI-A NTTTAAGALE LTCVRQKIRH VVV AGHSDC KAMRLLYDLR KQMDA-----
XP_045201158.1        GD NYMVKNA GNFVPYPDDL K--------- ------QI-A NTTSAAGALD LTCVRNNVRH VVV AGHSDC KAMSLLYDLR TEMDI-----
XP_009053308.1        GD HFMVRNV GNMIPDADTL N--------- ------T--D TVSTEAGALE FCCVQKNVKH VIV CGHSDC KTVSMLYEKC VAEEF-----
XP_033096127.1        GD IFFVRNP GNVVVPSNLC S--------- ------L--S DSVEGPAALD MACNVVKVKD VVV LGHSDC KAMNALYSLC SCTKHKD---
XP_013785798.1        GD MFVVRNA GNLVPSSNHQ S--------- ------M--D FFSNEPAAME LACVLTGVRH VVI CGHSDC KAMNRLYKMH EEETL-----
GAU97340.1            GE MFIVRNA GVLIPHYKYY G--------- ------KE-A TITTEPGALE LACVINTVQD IIV CGHSDC KAMNLLQSLK AENTPED---
OQV24824.1            GE MFIVRNA GVLIPHHKYY G--------- ------ND-A TITTEPGALE LACVINAVQD IIV CGHSDC KAMNLLQSLK EDKSCEV---
CAD5123161.1          GD MFIIRNA GNFIPKGEGA E--------- ------S--- --STEGGALE LGCVINKIRH VIV CGHSDC KAMHALYGMR DIINE-----
ELT97609.1            GD MYLVRNA GNLVPHCETL S--------- ------Y--D AVSTEPGALE LGCVMNEIPN VLV CGHSDC KAMNALYGMR DQTQI-----
XP_009013880.1        GD MFLVRNA GNLIPNNDSL S--------- ------F--D SVTTEPAALE LGCVINKIRH VLV CGHSDC KAMNALYEMM DSVQK-----
KAI0233246.1          GD MLLVRNA GNLVPHSDTL R--------- ------N--D EVSTEVAALE LGCVTTNIRH VVV CGHSDC KAMTALFEIR DKAEE-----
XP_029642194.1        GD AYIVRNA GNLFPPRDAI T--------- ------F--N SVTAEAGAIE VACVLNNVRH IFV CGHSDC KAMSLLFSLK NQCHT-----
NP_001171747.1        GD MFMVRNA GNLIPHSELF C--------- ------G--D SLNTEPAALE LACIKNDINH VIV CGHSDC KAMNCLYGIR NVTSH-----
CAH1781438.1          GD MFVVRNA GNMVPHSDFC S--------- ------Y--D HITTEPGALE LACIHNPIRH VIV CGHSDC KAINLLLNMR DKKAL-----
XP_013382323.1        GD VYLVRNA GNLVPHSKGL G--------- ------W--H NATTEPGALV LGCQMGDIRH VVV CGHSDC KAMNLLYSMR NELDH-----
XP_033759917.1        GD NFVVRSA GNLIPHACNF S--------- ------Y--E SATTEAGALE LGCIVNGVKH VVV CGHSDC KAMNLLYSMR KEVDN-----
XP_041373453.1        GE TFFVRNA GNIVPHVKIS S--------- ------KD-I GGSTEPGALE LGCVVNNIRH VVV CGHSDC KAMNMLYGIK HDMCN-H---
XP_046548515.1        GD NFFIRNA GNLIPNAKHF S--------- ------Y--E SVTTEPGALE LGCIINNIKH VVV CGHSDC KAMNALYGLR HDCHH-----
XP_047034441.1        GD MFVVRNA GNLVPHSRHF K--------- ------D--E MTSCEPAALE LGCITYDIRH IIV CGHSDC KAMNLLYKLK DDKET-----
CAB3245311.1          GD MFVVRNA GNVIPHSQYF V--------- ------D--E MTSCEPAALE LGCIVNNIRH IIV CGHSDC KAMNLLHSLR CKKES-----
XP_011549456.2        GD MFVVRNA GNVVPHSQYF S--------- ------D--E MTSCEPAAME LGCVINDVRH IIV CGHSDC KAMNLLYKLK SPQES-----
XP_045761472.1        GD MFVVRNA GNLIPHSQHF V--------- ------D--E MTSCEPAGLE LSCIINDIRH IIV CGHSDC KAMNLLYKLK SKEES-----
XP_046968321.1        GD MFVVRNA GNLVPHSQAF V--------- ------D--E MTSCEPAGLE LSCVLNDVKH IIV CGHSDC KAMNLLYKLK SDKES-----
XP_026323335.1        GD MFVIRNA GNVIPHSEYF L--------- ------D--E MTSCEPAGLE LACIINNIRH IIV CGHSDC KAMNLLYKLK SKEES-----
XP_045529909.1        GD MFVVRNA GNLIPHSEAF I--------- ------D--E MTSCEPAGLE LSCIINNVRH IIV CGHSDC KAMNLLYHLR SKEES-----
RVE42816.1            GD MFVVRNA GNLLPHSQHF M--------- ------D--E MTSCEPAGLE LACVINDIRH IIV CGHSDC KAMNLLYKLK SKEES-----
KAF9415294.1          GD MFVVRNA GNVVPHSQYF V--------- ------D--E MTSCEPAALE LGCILNDIRH IIV CGHSDC KAMNLLYKLK SKEES-----
XP_018897307.1        GD MFVVRNA GNLIPHSQHF L--------- ------D--E LTTNEPAALE LGCVVNDIRH IIV CGHSDC KAMNLLHALQ DVDFA-----
```

```
XP_019763455.1  GD MFIIRNA GNLVPHSQRF R--------- ------D--E QDTNEPAALE LGCVMNDIRH IIV CGHSDC KAINLLYKLR DPEFA-----
XP_030749737.1  GD MFIIRNA GNLVPHSHCF P--------- ------D--E QNSNEPAALE LGCVVNDIRH IIV CGHSDC KAINLLYKLR DPEFA-----
KAF7270051.1    GD MFIVRNA GNMVPHSQFF P--------- ------D--E QHSNEPAALE LGCVVNDIRH IIV CGHSDC KAINLLYKLR DPEFA-----
XP_031343879.1  GD MLIVRNP GNVIPNSQHF Q--------- ------D--E LTTNEPTALE LGCIVNNIRH VIV CGHSDC KAVNELYKLQ DREFG-----
XP_044743527.1  GD MFVVRNA GNLIPHSQHF M--------- ------D--E LTTNEPAALE LGCIINDIRH IIV CGHSDC KAMNLLYKLR DQEFA-----
XP_018319432.1  GD MFIVRNA GNVIPHSHHF L--------- ------D--E ITSNEPAALE LGCVINDIRH IIV CGHSDC KAINLLYQLQ DTNFS-----
XP_026466152.1  GD MFVVRNA GNLVPHSQHF L--------- ------D--E YISAEPAALE LGCVVNDIRH IIV CGHSDC KAMNLLYKLQ DNAFA-----
XP_022913687.1  GD MFVVRNA GNLIPHSQHF M--------- ------D--E LTTNEPAALE LGCIVNDIRH IIV CGHSDC KAMNLLHKLQ DAEFA-----
KAF5298473.1    GD MFVVRNA GNVIPHSQHF L--------- ------D--E LTTNEPAALE LGCIVNDIRH VIV CGHSDC KAINLLYKLQ DSEFS-----
VEN57246.1      GD MFIVRNA GNIIPHSQHF L--------- ------D--E ITMNEPAALE LGCVVNDIRH IVV CGHSDC KAINLLHKLQ DRDFS-----
XP_045472083.1  GD MFVVRNA GNIIPHSQHF E--------- ------D--E LTSNEPAALE LGCVVNDIRH IIV CGHSDC KAINLLYKMQ DSKFA-----
XP_017768645.1  GD MFVVRNA GNIIPHSQHF M--------- ------D--E LTTNEPAALE LGCVVNDIKH IIV CGHSDC KAMNLLFKLQ DSEFA-----
CAH1369701.1    GD MFVVRNA GNIVPHSQHF F--------- ------D--E LTTNEPAALE LGCVVNDIRH IIV CGHSDC KAINLLHKLQ DGEFA-----
XP_028136465.1  GD MFVVRNA GNVIPHSQHF V--------- ------D--E LTSNEPAALE LGCVVNDIRH IIV CGHSDC KAINLLYKLQ DSEFA-----
XP_023014099.1  GD MFIVRNA GNLIPHSQHF L--------- ------D--E LTTNEPAALE LGCVVNDIRH IIV CGHSDC KAMNLLHKLQ NVDFA-----
XP_019875064.1  GD MFIVRNA GNIIPHSQHF M--------- ------D--E LTTNEPAALE LGCVVNDIRH IIV CGHSDC KAINLLYKLQ DSEFA-----
CAH0557060.1    GD MFIIRNA GNIIPHSQHF M--------- ------D--E LTTNEPAALE LGCVVNDVRH IIV CGHSDC KAINLLYKLQ DSDFA-----
XP_037924690.1  GD MFVVRNA GNVIPHSHHF V--------- ------D--E FFSCEPAALE LGCIVNDIRH IIV CGHSDC KAMNLLYKLK DPEFA-----
XP_043863257.1  GD MFVVRNA GNLIPHAQHF Q--------- ------D--E YFSCEPAALE LGCVVNDIRH IIV CGHSDC KAMNLLYQLR DPDFA-----
XP_039965629.1  GD MFVVRNA GNVIPHAQHF Q--------- ------D--E YFSCEPAALE LGCVINDIRH IIV CGHSDC KAMNLLYKLQ DEEYS-----
XP_001989827.1  GD MFVVRNA GNLIPHAHHF H--------- ------D--E YFSCEPAALE LGCVVNDIRH IIV CGHSDC KAMNLLYQLR DPEFA-----
XP_037896985.1  GD MFVVRNA GNLIPHAHHF Q--------- ------D--E HFSCEPAALE LGCVVNDIRH IIV CGHSDC KAMNLLYMLR DPDFA-----
XP_046805044.1  GD MFVVRNA GNLIPHAQHF Q--------- ------D--E HYSCEPAALE LGCVVNDIRH IIV CGHSDC KAMNLLYQLR DPEFA-----
KAG5681791.1    GD MFVVRNA GNLVPHSHFF Q--------- ------D--E YFSCEPAALE LGCVVNNIRH VIV CGHSDC KAINLLHQLQ CTQFA-----
XP_031629647.1  GD MFVVRNA GNLVPHAQHF Q--------- ------D--E YFSCEPAALE LGCVVNNIRH III CGHSDC KAMNLLYSLR DKEKA-----
KAG4077403.1    GD MFVVRNA GNLIPHAQHF Q--------- ------D--E YFSCEPAALE LGCVVNNIRH IIV CGHSDC KAMNLLYSLR DPELA-----
XP_039430644.1  GD MFVVRNA GNLVPHAEHF Q--------- ------D--E YFSCEPAALE LGCVVNNIKH IIV CGHSDC KAMNLLYQLR DPQFA-----
XP_040221405.1  GD MFVVRNA GNLVPHAEHF Q--------- ------D--E YFSCEPAALE LGCVVNNIKH IIV CGHSDC KAMNLLYKLK DPEFA-----
CAA9998239.1    GD MFIVRNA GNIIPHSHHF M--------- ------G--E VTMNEPAALE LGCVVNDIRH III CGHSDC KAMNLLYKLK DSTFS-----
KAF6203636.1    GD MFIVRNA GNVIPHSHHF N--------- ------D--E VTMNEPAALE LGCVVNDIRH III CGHSDC KAMNLLYKLK DQSFS-----
KAG6800307.1    GD MFVVRNA GNIIPHSQHF E--------- ------D--E LAMCEPAALE LVCLMNEIKH IIV CGHSDC KAMNMLYSLR EEELA-----
XP_014230599.1  GD MFVVRNA GNIVPHSSHF M--------- ------D--E LTMCEPAALE LGCVVNDIRH III CGHSDC KAMNLLYALR DQEFA-----
KAH0947068.1    GD MFVVRNP GNLIPHPHHF V--------- ------D--E YTTCESAALE LSCVVNAIRH VIV CGHSDC KAMNLLYTLR DEELA-----
XP_043590797.1  GD MFVVRNA GNLIPHSQHF V--------- ------D--E LTMCEPAALE LGCVINNMKH IIV CGHSDC KAMNLLYALR DEEFA-----
XP_034949379.1  GD MFVVRNA GNVIPHSQHF I--------- ------D--E LTMCEPAALE LGCVINNIRH IIV CGHSDC KAMNLLYALQ DEEFA-----
XP_012275169.1  GD MFVVRNA GNIVPHSQHF A--------- ------D--E LTMCEPAALE LGCVVNNIKH IIV CGHSDC KAMNLLYALR DQEFS-----
XP_043464528.1  GD MFVVRNA GNIVPHSQHF L--------- ------D--E LTMCEPAALE LGCVVNDIRH VIV CGHSDC KAMNLLYALR DEEFS-----
XP_014220045.1  GD MFVVRNA GNIVPHSHHF D--------- ------D--E LTMCEPAALE LGCIVNDIRH VIV CGHSDC KAMNLLYALR DENFA-----
XP_011160605.3  GD MFVVRNP GNVVPHSRHF V--------- ------D--E FTMCESAALE LGCVVNDIKH VIV CGHSDC KAMNLLYALR DEEFA-----
XP_046741789.1  GD MFVVRNA GNVVPHSQHF S--------- ------D--E LTMCEPAALE LGCVVNDIRH IIV CGHSDC KAMNLLYALR DEEFA-----
VVC24333.1      GD MFIVRNA GNLIPHSQHF L--------- ------D--E YTTCEPAALE LGCVHNDIRH VIV CGHSDC KAMNLLHLLR DTEFA-----
KAF0770685.1    GD MFIVRNA GNLIPHSQHF Q--------- ------D--E YTTCEPAALE LGCIHNDVRH VIV CGHSDC KAMNLLYLLK DTEYG-----
XP_015365968.1  GD MFIVRNA GNLVPHSRHF Q--------- ------D--E YTSCEPAALE LGCVHNDVRH VIV CGHSDC KAMNLLHLLR ETEYS-----
XP_029341681.1  GD MFIVRNA GNLIPHSQHF P--------- ------D--E YTSCEPAALE LGCVHNDIRH VIV CGHSDC KAMNLLHLLR DTEYG-----
XP_026292519.1  GD MFVVRNA GNIIPHCGHF L--------- ------D--E YHTNEPAALE LGCIVNDIRH IIV CGHSDC KAMNLLYALR DAEMS-----
XP_034238427.1  GD MFVVRNA GNVIPHCGHF L--------- ------D--E YHTNEPAALE LGCIINDIRH IIV CGHSDC KAMNLLYALR DAEMS-----
XP_024081347.1  GD MFVVRNA GNIIPHAHHF R--------- ------D--E ITTNEPAALE LGCIVNDIRH IIV CGHSDC KAMNLLYKLR DEEFS-----
XP_014283188.1  GD MFVVRNA GNIIPHSQHF L--------- ------D--E YTTNEPAALE LGCVVNDIRH IIV CGHSDC KAMNLLYKLR DENFA-----
RZF32743.1      GD MFVVRNA GNLIPHSQHF L--------- ------D--E YTTNEPAALE LGCVVNDIRH IIV CGHSDC KAMNLLHKLR DEKFA-----
CAB3380083.1    GD MFVVRNA GNLIPHSQHF V--------- ------N--E AFTTEPAALE LGCIVNDIKH VIV CGHSDC KAMNLLHSLQ DEEYA-----
XP_046989067.1  GD MFVVRNA GNVIPNSQNF P--------- ------E--E VTTTEPAALE LGCVINDIRH VIV CGHSDC KAMNLLHRLK CEEFS-----
GFG38476.1      GD MFVVRNA GNVIPHSQHF L--------- ------D--E ATMNEPAALE LGCIVNDIRH VIV CGHSDC KAMNLLYSLQ CQEFS-----
CAD7446618.1    GD MFVVRNA GNLIPHSQHF Q--------- ------D--E KTTTEPAAME LGCIINDIKH IIV CGHSDC KAMNLLHALR DEEFS-----
KAG8226740.1    GD MFIVRNA GNVIPHSQHF V--------- ------N--E MTTTEPAALE LGCIVNDIKH IIV CGHSDC KAMNLLHALR DEEFS-----
XP_046392794.1  GD MFFVRNA GNLIPHSQHF I--------- ------N--E MTTTEPAALE LGCIVNDIRH IIV CGHSDC KAMNLLHDLR NEEFS-----
XP_046459477.1  GD MFIVRNA GNLVPHSKLY G--------- ------I--D SATTEPAALE LGCIVNNVKH MVV CGHSDC KAMNLLYSFK KGIET-----
XP_043227363.1  GD MFIVRNA GNLVPHAKLY D--------- ------T--E TVSTEPAALE LGCIVNGIKN VII CGHSDC KAMNLLYDLR DPALA-----
XP_018006429.1  GD MFIVRNA GNLVPHAMLC G--------- ------G--E EITTEPAALE LGCVINGIKH VIV CGHSDC KAMNMLHLLR NSDMS-----
AWH57222.1      GD MFIVRNA GNLVPHANLS G--------- ------H--E EITTEPAALE LGCVINGIRH VIV CGHSDC KAMNMLHLMR NSDRT-----
XP_042865604.1  GD MFIVRNA GNLVPHANLC G--------- ------H--E EITTEPAALE LGCVINGIRH VIV CGHSDC KAMNMLHLLR NTDRL-----
XP_045119740.1  GD MFIVRNA GNLVPHANLS G--------- ------H--E EITTEPAALE LGCVINGIKH VIV CGHSDC KAMNMLHMMR NSDRT-----
XP_045613573.1  GD MFIVRNA GNLVPHANLC G--------- ------H--E EITTEPAALE LGCVINGIKH VIV CGHSDC KAMNMLHMMR NSDRT-----
XP_021963432.1  GD MFIVRNA GNLIPHSKFF A--------- ------T--E NTSTEPAALE LGCIINEIKH VIV CGHSDC KAMNLLYDLG TDKEMS----
CAG7730563.1    GD MFIVRNA GNLVPHSKFF V--------- ------A--E NTTTEPAALE LGCVINKIQH VVV CGHSDC KAMNLLYTLK DEALT-----
```

```
               271
KAH8855123.1   LSHHYHQSNS IDKEVNKLKS SPLEKWIWEN GC-K TLQNF HLKSN----- -----ILSFT -SSLHS-NCP KLELDLNLVK HLTEIDLLSQ
KAH9593836.1   LAHHEHQPID VQTENNKITS SPLEKWIWEN GC-K TLQNF NTKSG----- -----ILSFK SSSIHS-KCP TLELDLNSAK HLTEVDLLSQ
VDP71164.1     ---------- --GQLVEKQM SPLQKWVAGQ GI-K CWKKW QSKTN----- -----AVRFP AFKEE---QL ILNMRSDLIK NLEPVDIISQ
TPP65121.1     ---------- --KKLDEQKI SPLQKWISQN GV-K CWKKW HAKTN----- -----TVQFS TLE-D---HL TLNMRSDAMR NLEPVDVLSQ
KAA0189413.1   ---------- --HKSDKNSA SPLQKWVSQN GI-K GWNKW RAKSD----- -----SIEFP AMK-D---HL TLNMHADQVK KLEPVDVLSQ
KAF7258038.1   ---------- --DKLQTTQM TPLQQWAAKN GM-E GFRKY ERKPN----- -----LLEFP VNDMLT-LKT PLRLKLTSLK DLSDVDRLSQ
KAG5442206.1   ---------- --GELPVSQM SPIEKWVALN GL-A SYRKH TLTSD----- -----VLHFP VVDPQV-RKS NFNLKLSSLK DFEECDRLSQ
XP_030837801.1 ---------- -TGSGSQHSM DLMNNWLRAY GS-P ALEKY ERHME----- ----NPAEEV TYEGGGRKGA KLSAVIEDNG KLSKTDRLAQ
XP_041482585.1 ---------- -AGSGSQHSL DLTNNWLHSY GS-P ALEKY EQHME----- ----KPGEEV EFEGGGRKGA KFSAIIDDEN KLSKTDKLAQ
KRZ73557.1     ---------- ------WKPD KPLKSWLQIH GS-P SVQKY NCLMK----- -----GQQVF QFIPEY-PLL QFSAKIDPSG KLANADKLSQ
OUC42378.1     ---------- ------WKPD EPLKSWLQIH GS-P SVQKY NYLLK----- -----GQQVL QFIPEY-PFL QFSAKIDPSG KLTNADKLSQ
CDW52968.1     ---------- ------WDSR MPLNAWLMLQ GR-A TLEKY EQMEE----- -----TDGSI SFLNKW-QSL SFEASIDR-K KWNELDQLSQ
KHJ46144.1     ---------- ------WDLH KPLSSWLMLQ GK-A TLEKY EEMEE----- -----THGSI SFLNKW-QNL SFEASIDR-K KLNELDQLSQ
VZI00627.1     ---------- -----LTRYS SPLERWVVQM GK-S SLEAF LRSDA----- -------LTI TFDLGSD-FR PMNATVDAER RFSPVDRLSQ
XP_014681778.1 ---------- ------GERE GPLHTYLRSY AV-P TIMKM QQLLRS---- ---PNMEGPL EFSVTPR-HR SFRAFIDPEH ELSEMDKLSQ
VDM45293.1     ---------- ------FDES SPLHHWVRKH GY-V SLHKL EQRLK----- ---EGASCRL VFAENDR-HQ SFKALIDPEN ELDVEDKLSQ
KAF8385568.1   ---------- ------FDHA SPMDHWLRKN GH-R TMKKL NERLY----- ----KGPQPL QFDSEVAPSQ SFEAIIDPFD RLKAEDKLSQ
PIO76965.1     ---------- ------FDMD SPMDHWVRRN GF-R SMKRL NERLH----- ----KGAKIM KFETDIAPSQ NFEAIIDPFD KLPVEDKLSQ
VDL66086.1     ---------- ------FDAS SPMDHWVRQN GY-R SVKRL TERLH----- ----KGGTPL KFESSVAPSQ NFEAIVDPFD LLPVEDKLSQ
CAD6194288.1   ---------- ------FDVS SPMDQWVRNN GY-Q SIKKL NERLH----- ----KGAAKM TFNCEMSPNQ SFEAIIDPMD RLPVEDKLSQ
XP_045095542.1 ---------- ------FDVS SPMDQWLRRN GF-E SMKKL NERLH----- ----IGPKTM KFESEVAPSQ SFEAIIDPME KWSAEDKLSQ
EGT43056.1     ---------- ------FDVT SPMDHWVRRN GF-A SVKRL NERLH----- ----LGPSNM SFESEVSPSQ SFEAIIDPMD RLPVEDKLSQ
CAB3400100.1   ---------- ------FDVE SPMDHWVRRN GF-K SVQRL NERLH----- ----KGPSLM KFESEVSPSQ SFEAIIDPFE KLTVEDKLSQ
KAF7639475.1   ---------- ------FDPE SPMDHWLRHN GH-L SIQKL EKLIKN---- ---EKSSKKL EFASENG-LM KFKAKIDPEN KYGVEDKLSQ
CAD5226593.1   ---------- ------FDQT SPVDHWLRKN GF-R SHIKL TQMLKAE--- ENCDPDARRI HFAAEEPELL NFHAQIDPDR KFGVEDWLSQ
XP_024510735.1 ---------- ------FSEK SPMDRWLRLN GY-H SLKKL EMREN----- ---DGPNVPL KFENDDQ-KF CFYAYIDKEN KLNIEDKLSQ
VDN24694.1     ---------- ------FDAN SPLDLWIREH GF-L SLKKL EERLA----- ---SKTAKPL KYSTPDG-AF SFEAIIDEEN KFDVEDKLSQ
KAH7730640.1   ---------- ------FDPE SPMDHWLRKH GY-S SIRKL EELKRTD--- ---STGAKPI EFVAENPSLL RFHAVIDEER RFNVEDRLSQ
TMS38138.1     ---------- ------FDPA SPMDHWLRRH GF-T SVQKL ENRLK----- ---SEPEKPL VFLSDFP-YF KFEAVIDPQN EFNTEDKLSQ
XP_013290959.1 ---------- ------FDPQ SPMDHWLRRH GF-T SIKKL EERLA----- ---DKNLKPL KFVSDNP-AH SFEAMIDEED KWSVEDKLSQ
RCN50005.1     ---------- ------FDPQ SPMDHWLRRH GF-A SLKKL EERLA----- ---DKTAKPL KFVSDNP-SF SFEAVIDPED KWGVEDKLSQ
KJH42542.1     ---------- ------FDPQ SPMDHWLRRH GF-T SIKKL EEHLT----- ---DESAKAL KFSSTGS-NF SFEAIIDQEN KWGVEDKLSQ
VDM64099.1     ---------- ------FDPQ SPMDHWLRRH GF-T SLKKL EGRLV----- ---DKSDKPL QFISDNP-VF NFEAIIDYED KWGVEDKLSQ
VDO63723.1     ---------- ------FDPQ SPMDHWLRRH GF-T SLKRL EERLA----- ---DKTAAPL KFVSDNP-VF NFEAIIDKED KLNVEDKLSQ
VDO44040.1     ---------- ------FDPQ SPMDHWLRRH GY-E SLQKL EERLA----- ----NTAKPL TFVSEVP-GY TFEAIIDPEN KWGVEDKLSQ
CAD6198090.1   ---------- ------FDPQ SPMDHWLRRH GF-H SLRRL EERLK----- ---AEKKTPL EFVVDNP-LF SFSAIIDEED KLNVEDKLSQ
KAF8373880.1   ---------- ------FDPE SPMDHWLRRH GY-N SLRKL EKRLA----- ---DDKAGPL EFVSSNP-LF SFSAIIDAEG KWNVEDRLSQ
XP_002632400.2 ---------- ------FDPE SPMDHWLRRH GF-N SIKKL EKRLA----- ---DKKAGPI EFVSDNP-LF SFQAIIDPED KLNVEDKLSQ
RDD39080.1     ---------- --------ES SFVLDWIYRF AS-Q TYTKW EKTTLVD--- --RSNSDQPL HLEFNE-NGL KFEAKIN--Q NLLPKDQLSQ
XP_001632619.1 ---------- --------TD NHYIPWLKKT GA-S SLTRF EKVDMSQ--- --EGGVKL-- LFEDAT-GGE PMEVTIDEGN KLDSVDKLSQ
XP_020895242.1 ---------- -------DNS SMIASWITTH GK-S SLTKL DRLVVBD--- --NK---V-- VFNDEI-ESE KFEAVID--E KLPIQDKLSQ
XP_027059688.1 ---------- -----QDSET SWVSGWLRRY AQ-S SLTKF EKLGF----- ---------P- IDGSSK-EKD NLEIMFDKEK DLSDVDKLSQ
XP_029213515.2 ---------- -------NAA SWVSLWLKRY AQ-A SLTKY EQLEVQG--- --QREGTVS- VGSGSK-DLE TLELMFDQEK MLPAVDKLSQ
GFO16554.1     ---------- -----ERTMD SPLKSWLSLH GL-Q TVQKF TKLFSEEDQQ RSARGETILG GFKFNF-GRE EFVCNVDPNN KFNLEDKLSQ
RUS89945.1     ---------- -----LQEPN SPLKSWLSAH GL-R TVHKF NELFSEEEQQ RSVKGETILG GFKLNF-GLD SFVCHVDPEN KFPIEDKLSQ
GFR80859.1     ---------- ---DQEHTVD SPLKTWLSAH GL-R TVQKF TKLFSEEEQL LSVEGKKILG GFKLNF-GKE EFLCAVDPQN DFSIEDKLSQ
XP_005106142.1 ---------- -----KYHPD SPLMSWVAFH GK-N TVKKF SDSQNTS--- ------TGPN TFTFSI-GGK EVKAIIDSEN QFDVQDKLSQ
CAG5118574.1   ---------- -----LYHQK SPLKTWVALN GR-R TANKF SQLFPDEVKR ---NNFVDPV IFDVNL-GQG HVKALIDPEN DFCIQDKLSQ
XP_013069769.1 ---------- -----QYIHT SPMKTWVALH GA-K TIEQF CNIYNDKDIP EDTYFMKQPM NFTVNL-NGK DIQTILDPCN KFNIKDKFSM
XP_013069770.1 ---------- -----HFLLD SPLRTWVALH GS-R TVEKF SKIYSEENQL NDSYHLKEPL HFTVNL-TGQ DVDAIIDPEN QFSIQDKFSQ
KAH9514813.1   ---------- -----KYLLE SPLKTWVALH GS-K TVVNF SKIYHEDDSL PNSYHLKQPL HFTVNL-NGQ DIDAIIDPEN QFCIEDKFSQ
XP_033638319.1 ---------- ---APLKRVS EIIHGWCRTH GVEA SLNKV QDTSN-R--- ---TSKGAAF KFNIGSTPQ- --EVFIDPKN EMALLDKLSQ
XP_022095223.1 ---------- ---KPITQVS QLIHQWSRTH GVEA SLNKI QDLSA-K--- ---TPEGGTM TFDVGAKPLE AYFQDFDIAE EKSLLDKLSK
XP_038051194.1 ---------- ---KPLKRVS QVIHQWCRTH GVEA SLNKL QDLSA-R--- ---TPSEGVM TFDVGATPIE AYFKDFDSTD EKSLLDKLSK
ACO15530.1     ---------- -----EELLQ SPLKAWLYKH GM-D SLNKL NDKLT----- ---S-PESPL TFMKDT--QH EFEANMD--N KLLESDQLSQ
XP_040566914.1 ---------- -----FDVMQ SPLKAWLQRN GM-V SFKRF CEMKK----- ---MGKEDSL IFMKNT--KH EFEARID--S QLDEADQLSQ
VDP33652.1     ---------- ------LDHS SPVDLWLRGN AA-S SWERF QKFMN----- -----NRQPL HYMQTS-DQL SIEVHIDVER MLDEKDTLSQ
PAA53847.1     ---------- -------QRP DALSEWVRKY GR-S SLKHY LAERD----- ------AQPM KFRSPI-EGQ SISAHIDPDK RYQPHDQLSQ
RNA13068.1     ---------- -----KSEKE SLLKSWLMAN SA-P TIKKY SEFEK----- ---NSFKKTS SFSIVD--GQ KFEAYIDPEN EYGNNDKFSM
CAF0715750.1   ---------- -----TTEKE SVLKSWLMAN SV-P TIHKY FEYEK----- ---GDFKKPA KFSITN--GR TFEAYIDPEN AYPNNDKFSM
CAF0950609.1   ---------- -----ESNNP SALKKWLILN GK-D SVVKY KEFEK----- ---SGFNKCL TFSEGE--PN QFDAYIDPDN QFSPQDKFTM
CAF1119542.1   ---------- -------PSK GPLEDWLRFH GR-G TIEQF KKLES-S--- ---SGFDRKL VFAGAQHSED DFEAYIDPRN EFKPSDKFSQ
CAF1301217.1   ---------- -------PSK GPLEDWLRVH GR-G TIEQF KKLES-T--- ---TCFERKL IFAGAQHYED DFEAYIDPYN QFKPSDKFSQ
CAF3941029.1   ---------- -------PSK GPLEDWLRVH GR-G TVEQF KKLEM-S--- ---TGFDRKL KFAGAQHYED DFEAYIDPHN QFKPSDKFSQ
CAF0867361.1   ---------- -------PSK NPLDQWLKVH AK-G TVEQF QKLES-S--- ---SDFKRKL VFASAQHHED DFEAYIDPNN EYKPSDKLSQ
UJR16523.1     ---------- -------PSK NPLEQWLKTH AK-G TVEQF QKLEL-S--- ---GDYRQKL VFASAQHHED DFEAYIDPNN EFKSSDKLSQ
CAF1340180.1   ---------- -------PSS NPLEKWLKVH AR-G TVEQF KKLES-S--- ---GDYTKKL IFSSAQHHED DFEAYIDPNN EFKHSDKLSQ
CAF1025683.1   ---------- -------PSN TPLEQWLKVH AQ-G TVEQF KKLES-S--- ---GDYKCKL IFASAQHHED DFEAYIDPNN EFKHSDKLSQ
ALS04255.1     ---------- -----SAAAR GPLSAWITQH GA-S TMHEF EKIER----- ---ALFRRPI LLHAED-PNN KFPAYVDVDE KYNINDKISM
XP_023329171.1 ---------- -----SAVAH GHLSSWIKQH GD-S TMVEF DKIER----- ---AQFRRPI LLHAED-PNE KFPAYVDVDE KYNINDKISM
KAH3870454.1   ---------- -------KTG SPLELWIKKN GW-R TIEKF QYLKE-E--- ---NSFTGPV PFMQES-ENT KFKITIDPGQ QFNIVDKLSQ
XP_045201158.1 ---------- -------PSG SPLELWIKKH GK-R TIDKF QHLKE-E--- ---NGFIGPV PYIQEK-EDK KFEIYIDPDH NFNMVDKLSQ
XP_009053308.1 ---------- -----SKSPT TFLEMWLKLH GR-N TLKAF NQLQE-H--- ---NNFMGPL KFDIEN--YK SFEAYIDPEN KFSIVDKFSQ
XP_033096127.1 ---------- ---DDEVPPD SPVSRFLNQV GM-A TIEKF EAVRK----- ----CEPGAL HFKSEV-PSL SFRAFIDPEK QLSLTDKLSQ
XP_013785798.1 ---------- ------MELK GSLHGWLCQH GI-A SLDRF IELEK----- ---NNFKNPL QFFEKN-TSK KFRAFIDPEN KFSHVDKLSQ
GAU97340.1     ---------- -----TDFAH SPLRSWLMRY GG-D SWVQF EKMLQ----- ----SKKNRL VFMEG---NH AYEAVIDAKN EFNMADKFSQ
OQV24824.1     ---------- -----KNFHD SPLRSWVWRY GG-D SYTHF ETMLR----- ----AEKNRL VFKEG---NH AHEAVIDPRN QFSIADKFSQ
CAD5123161.1   ---------- ------PKKE SPLTSWIQKC GK-P TLKKF GELEK-S--- ----DGRGPI SFSEE---LK PFTAKID--T SFSTVDRLSM
ELT97609.1     ---------- -------KEG TPLTLWLKKH GH-S SVKRY NELLN-S--- ---PDGIGPL EFKI---PGK TLRAYIDPDK KLSEVDKLSQ
XP_009013880.1 ---------- -------HEG TPLQIWLKRH GA-R TIVKY KELLQ-V--- ----GGVGPI KFQAET-PEK VFDAYIDVDN KFKPVDKLSQ
KAI0233246.1   ---------- -------VGG PPLASWIKKH GL-A SVKKY RQLEA-A--- ----GGVGPI TFEAEN-TSK RFTAFIDRDN QFAPVDKFSQ
XP_029642194.1 ---------- -------HSG TPLQMWLKKY GV-A SVRKF DQLDS----- ----KFVGPL TFQGRF-SQL NFQVNIDPEN KFPVEDKLSQ
NP_001171747.1 ---------- ------DKDK NPFAAWLAKF GK-A SLNAF KEVER----- ----GSKSPL KFTGET-PKH NFEAFIDPDD KFGIEDKLSQ
CAH1781438.1   ---------- -----ENKDG TPLENWVKKH GL-S SVMKF EQLENLG--- ---DKGSGPI IFQGAS-PKH TFEAFIDPQN EYSILDKLSQ
XP_013382323.1 ---------- -------HDG DALQLWLRRH GS-S SVQKF EQWEK-M--- ----KGREPI TFQAET-PRH NIEALIDPEN NFGIVDKFSQ
XP_033759917.1 ---------- -------KEG GPLQLWLKRH GT-A SLEKF KLLTP-E--- ---NEYKGPV PYQT----AR KFNAFIDPEN RFNLADKLSQ
XP_041373453.1 ---------- -----AACSH SPLRAWLQKH GA-P TVKKF AKLSP-E--- ---NNFSGPL IFQGET-EEN SFAAYIDPEN NFSLPDKLSQ
XP_046548515.1 ---------- -------QVG TPLQMWLKKH GQ-P TVSKF EKLEA-G--- ---NNYSGPL TFQGET-EKS NFDAYIDPEN KFSMADKFSQ
XP_047034441.1 ---------- ---TTEKRRI SPLTSFMCSY AK-S SLDKF LKMN------ ---GDFTKPV KFTAET-PHR KFVAYIDPEN KFCIEDKLSQ
CAB3245311.1   ---------- ---SIEQRRL SPLKSWLATH AT-T SLEKF LSMK------ ---GDFSKPM LFTAET-PQR KFVAYIDPDN KFCIEDKLSQ
XP_011549456.2 ---------- ---NIEQRRI SPLKSWLCNH AK-T SLDKF LAMN------ ---GDFKKPM IFTAET-EQR KFVAYIDPED KFCIEDKLSQ
XP_045761472.1 ---------- ---SLEQRRI SPLKSWLCSH GK-S SLDKF LDIG------ ---GDFEKPI LFTAET-PQR KFVAYIDPDN KFCIEDKLSQ
XP_046968321.1 ---------- ---SIEQRRI SPLKSWLCTH GK-S SLDKF LDVK------ ---GNFDKPI VFSAET-PQR KFVAYIDPEN NFCIEDKLSQ
XP_026323335.1 ---------- ---SKEQRRI SPLKSWLCNH GE-S SLDKF LAMN------ ---GDFSKPM LFSAET-PHR KFVAYIDPDN TFCIEDKLSQ
XP_045529909.1 ---------- ---NVEQRRI SPLKSWLCTH AQ-S SLKKF LEMK------ ---GDFNKPM LFSAET-PQR KFVAYIDPEN KFCIEDKLSQ
RVE42816.1     ---------- ---NKEQRRI SPLKSWLCTH GQ-S SLEKF IEMN------ ---GDFTKPM LFTAET-PQR KFVAYIDPEN QFCIEDKLSQ
KAF9415294.1   ---------- ---SIEQRRL SPLKSWLCSH AH-T SLKKF IEMN------ ---NDFTKPM LFTGET-PQR KFVAYIDPDN KFCIEDKLSQ
XP_018897307.1 ---------- ---SKENRRI SPLRAWLCTH AM-S SLEKY QQLQV----- ---AGFHTPL IFQSET-PLR KFVAYIDPEN KFSVEDKLSQ
```

```
XP_019763455.1  ---------- ---SKENRRL SPLRSWLCTH AQ-S SIDTF NELEK----- ---HNYDKPL LFQGET-PLR KFAAYIDPEK KFSIEDRLSQ
XP_030749737.1  ---------- ---SKENRRL SPLRSWMCTH AQ-S SIDKF EESIP----- ---NDFNKPL LFQGET-PLR KFVAYIDPDK KFSIEDRLSQ
KAF7270051.1    ---------- ---SKDNRRL SPLRSWMCAH AQ-T SIEKF EDLLA----- ---DDFNKPL LFQGET-PLR KFAAYIDPEK KFNIEDRLSQ
XP_031343879.1  ---------- ---SPENRKL FPVRSYLCTH AL-P SLEKF QQFQL----- ---TDYQKPL LFQAET-PMK HFVAHIDPDN KFAFEDKLSQ
XP_044743527.1  ---------- ---SLTNRRI SPLKAWLCTH AQ-S SLDKF MQLKL----- ---SDYKMPL IFQAET-PMR KFVAYIDPDD KFSIEDKLSQ
XP_018319432.1  ---------- ---SQVNRRK SPIRAWLCAH AW-S SLEKF QQLEL----- ---TDYSMPL IFQAET-PQR KFVAYIDPEN KLALEDKLSQ
XP_026466152.1  ---------- ---SQDNRRI SPLRAWLCSH AQ-S SLDKF QQLAL----- ---SDYKTPL IFTAET-PLR KFVAYIDPED KFSVEDKLSQ
XP_022913687.1  ---------- ---SLRNRRI SPLRAWLCTH AL-S SLEKF QQLQL----- ---TDFAKPL LFQAET-PMR KFVAYIDHDN KFAIEDKLSQ
KAF5298473.1    ---------- ---SLKNRRL SPLRSWLCTH AL-P SLEKF QQLEV----- ---TDYQKPL IFQAET-PMR KFVAYIDPDN EFALEDKLSQ
VEN57246.1      ---------- ---SQDNRRK SPLRAWLCAH AL-S SLEKF QQLSM----- ---TDYSMPL IFQAET-PMR KFVAYIDPDN KFNIEDKLSQ
XP_045472083.1  ---------- ---SQENRRL SPLRAWLSSH AC-T SLEKF QQLEI----- ---GDFTNPL IFQAET-PMR KFVAYIDPDN QFTLEDKLSQ
XP_017768645.1  ---------- ---SQENRRI SPLRSWLCTH AL-S SLEKF QQLEI----- ---TDYSKPL LFQGET-PLR KFVAYIDPEN KFALEDKLSQ
CAH1369701.1    ---------- ---SQDNRRI SPLRAWLCTH AL-T SLEKF QQLEV----- ---TDFGKPL IFQAET-PLR KFVAYIDPEN RFALEDKLSQ
XP_028136465.1  ---------- ---SQENRRI SPLRAWLCTH AL-S SLEKF QQLEV----- ---TDYSKPL IFQAET-PMR KFVAYIDPEN KFAIEDKLSQ
XP_023014099.1  ---------- ---SKDNRRI SPLRAWLCTH AL-S SLEKF QQLEV----- ---TDYSKPL IFQAET-PLR KFVAYIDPEN KFNIEDKLSQ
XP_019875064.1  ---------- ---SQDNRRI SPLRAWLCSH AL-S SLEKF QQLQV----- ---TDYSKPL IFQAET-PLR KFVAYIDPDN QFSIEDKLSQ
CAH0557060.1    ---------- ---SQVNRRI SPLRAWLCTH AL-S SLEKF QQLEL----- ---TDYNKPL IFQAET-PLR KFVAYIDPEN KFNIEDKLSQ
XP_037924690.1  ---------- ---SLDNRRI SPLRSWLCTH AQ-S SLDRF QKWVA----- ---GGMKDPL IFSSET-PLR KFVAYIDPEN KFAIEDKLSQ
XP_043863257.1  ---------- ---SKLNRRL SPLRSWMCTH AN-S SLEKF QEWRD----- ---AGMKDPL IFSSET-PLR RFVAYIDEEQ KFAVEDKLSQ
XP_039965629.1  ---------- ---SQLNRRL SPLRSWMWTH AS-S SLDHF KAWKK----- ---EGMQGPL IFSSET-PLR RFVAYIDPEN KFALEDKLSQ
XP_001989827.1  ---------- ---SKLNRRL SPLRSWLCTH AN-T SLDRF QEWRD----- ---AGMKDAL VFSSET-PLR RFVAYIDQDD KFAIEDKLSQ
XP_037896985.1  ---------- ---SKNNRRL SSLRSWLCTH AT-T SLEKF LEWGE----- ---KGMRDPL IFYSES-GLR RFVAYIDPDN QFAIEDKLSQ
XP_046805044.1  ---------- ---SKRNRRL SPLRAWLCTH AS-T SLEKF EEWVE----- ---KGMSDPL LFSSET-PLR RFVAYIDPEN KFAIEDKLSQ
KAG5681791.1    ---------- ---SRKNRRL SPLRSWLATH AS-T SLEKF QYLSE----- ---HGFDKPL MFTAET-PLR KFVAYIDPDN KFAIEDKLSQ
XP_031629647.1  ---------- ---SADQRRI SPLKAWLCAH ATDS SLDRF LKWQE----- ---AGMKDPL IFSSET-PLR KFIAYIDTEN KFGIEDKLSQ
KAG4077403.1    ---------- ---SLDNRRI SPLKAWLCEH AN-S SLDRF LEWKN----- ---NGMKDPL LFSSET-PLR KFVAYIDPDN KFAVEDKLSQ
XP_039430644.1  ---------- ---SRKNRRI SPLRAWLCEH AD-T SLEKF QNLQE----- ---TGLDKPI IFSSET-PLR KFVAYIDPEN QFAIEDKLSQ
XP_040221405.1  ---------- ---SLDNRRI SPLRAWLCEH AN-T SLAKF QNLKE----- ---IGLDKPL IFSSET-PLR KFVAYIDPEN NFAIEDKLSQ
CAA9998239.1    ---------- ---SKTNRQL SPLRAWLCNH AH-T SLEKY HQLEL----- ---CGTNRPL MFSSEL-PLK KFAAYIDPEK KWSVEDRLSQ
KAF6203636.1    ---------- ---SKTNRQL SPLRAWLCNH AH-S SLDKF NQLEL----- ---CGTTRPL MFQSEM-PLK KFVAYIDPEK RWSVEDRLSQ
KAG6800307.1    ---------- ---SKVNRRI SPLKAWLCAH AS-N SLTRF QQLEI----- ---SDFRDPI LFQGET-SLR KFVAYIDPED KFGVEDKLSQ
XP_014230599.1  ---------- ---SQKNRRI SPLRAWLCAH AS-N SLEKF EQLRV----- ---SGYEEPL IFRAET-PMR KIVAYIDPEN RFAVEDKLSQ
KAH0947068.1    ---------- ---SQTNRRM SPLRAWLYAH GS-S SLAKF QHLEI----- ---CGFHQPI VFQAET-SVR KFVAYIDPEN KLAVEDKLSQ
XP_043590797.1  ---------- ---SKVNRRI SPLRAWLCAH AR-N SLAKF QQLEI----- ---AGFHEPI LFQAET-PLR KFVAYIDSDN KFAIEDKLSQ
XP_034949379.1  ---------- ---SKINRRI SPLRAWLCAH AD-S SLRKF LQLKK----- ---TSFREPI IFQAET-PMR KFVAYIDPDD KFAIEDKLSQ
XP_012275169.1  ---------- ---SQANRRI SPLRAWLCAH AS-S SLAKF QQLQE----- ---KGFQEPT LFQAET-PLR KFVAYIDPED KYPVEDKLSQ
XP_043464528.1  ---------- ---SQNNRRI SPLRAWLCAH GS-S SLAKY QQLEV----- ---TGFREPI VFQAET-PLR KFIAYIDPEN KFVIQDKLSQ
XP_014220045.1  ---------- ---SQANRRL SPLRAWLCAH GT-S SLAKF QQLEI----- ---TNFKEPL MFQAET-PMR RFVAYIDPEN KFAVEDKLSQ
XP_011160605.3  ---------- ---SQTNRRM SPLRAWLCAH AS-N SLAKF QHLEI----- ---TGFREPI LFQAET-PMR KFVAYIDPED KFAIEDKLSQ
XP_046741789.1  ---------- ---SQANRRI SPLRAWLCAH AS-S SLAKF QQLEI----- ---TGFHEPI IFQAET-PLR RFVAYIDPED KFAIEDKLSQ
VVC24333.1      ---------- ---SIDNRRI SPLRSWLCTH GI-S SLDKY MQLEA----- ---AGFDTPL VFQAET-PLR KIIAYIDPEN KFSVTDKLSQ
KAF0770685.1    ---------- ---SIINRRM SPLRSWLCTH AM-S SLEKY EQLEE----- ---SGFNTPL IFQAET-PLR RISAYIDLGN KFSVTDKLSQ
XP_015365968.1  ---------- ---NIANRRM SPLRSWLCSH AM-S SLEKY QQLEV----- ---AGFHAPL IFQAET-PLR RISAYIDPDD KLSVTDKLSQ
XP_029341681.1  ---------- ---STVNRRK SPLRAWLCSH AM-S SLEKY QQLEA----- ---AGFGTPL VFQAET-PLR RISAYIDPED KLSVTDKLSQ
XP_026292519.1  ---------- ---SQTNRRL SPLRSWLCTH AH-S SLEKF QQLAL----- ---TGYHSPL IFQAET-PMR KFVAYIDPDD KFAIPDKLSQ
XP_034238427.1  ---------- ---SQKNRRL SPLRSWLCTH AH-S SLEKF QQLAL----- ---TGYQHPL IFQAET-PMR KFVAYIDPDD KFAIPDKLSQ
XP_024081347.1  ---------- ---SKDNRRI SPLRSWLCNH AY-S SLEKF QELEI----- ---SGYKDPL IFQSET-PLR KFVAYIDHEN RFSTEDKLSQ
XP_014283188.1  ---------- ---SKDNRRI SPLRSWLCTH AS-S SLEKF HQLEA----- ---GGYHNPL IFQSET-VLR KIVAYIDPED DFCIEDKLSQ
RZF32743.1      ---------- ---SKDNRRI SPLRAWLCTH AQ-S SLDKF QQLEL----- ---AGYHTPL IFQSET-PMR KFIAYIDPDD RFSIEDKLSQ
CAB3380083.1    ---------- ---SVDNRRK SPLRSWLCRH AD-T SLEKF QQLKS----- ---TNFTEPL TFQAET-PMR KFVAYIDPDN KFSIEDKLSQ
XP_046989067.1  ---------- ---SRDNRLI SPLRAWLIAH AN-A SLKKF EKLET----- ---GGFHQPL LFQAET-PMR KFVAYIDPDN KFSIEDKLSQ
GFG38476.1      ---------- ---SQTNRRI SPLRAWLSAH AS-S SLAKF QQLEV----- ---SGYQKPL IFQAET-PMR KFVAYIDPED KFSVADKLSQ
CAD7446618.1    ---------- ---SQENRRK SPLRAWLCAH AS-S SLEKF TQLEA----- ---SGYHTPL IFQAET-PMR KFAAYIDPDD KFAITDKLSQ
KAG8226740.1    ---------- ---SVENRRI SPLRAWLCAH AH-S SLTKY QQLET----- ---SGFGTPM LFQAET-PMR KFVAYIDPEN RFSVTDKLSQ
XP_046392794.1  ---------- ---SVENRRR SPLRAWLCTH AQ-S SLTKF QQLQK----- ---TGYSSPM LFQAET-PMR KFVAYIDPEN RFSVTDKLSQ
XP_046459477.1  ---------- ---NMRTLER SPLKAWLHRH GS-I SLTKF ERLEV----- ---HGFQQPL TFPMEG-PFR QFVAYIDPDN KFSLTDKLSQ
XP_043227363.1  ---------- ---TPEKLHM SPLSAWVCRH GS-G SLTKL MQLKV----- ---SDFKAPL VFEGEL-PMR RFVAYIDPEN QFSITDKLSQ
XP_018006429.1  ---------- ---TREIVKQ SPLKAWLVRH GH-D SLIKY AQLEL----- ---NDFKQPL VFQGET-PLR RFVAYIDPDD QFSPEDKLSQ
AWH57222.1      ---------- ---SQEIIKH SPLKAWLVRH GH-S SIAKY AQLEV----- ---ADFQAPL IFQGET-PMR RFVAYIDPED KFNEEDKLSQ
XP_042865604.1  ---------- ---HHEIIRM SPLKAWLVRH GS-S SLAKF AQLEV----- ---ANFQAPL IFQGET-PMR RFVAYIDPDN HFNDEDKLSQ
XP_045119740.1  ---------- ---LQEMLKL SPLKAWLVRH GH-S SIVKF AQLEV----- ---SDFQAPL VFQAET-PMR RFVAYIDPDN RFSVEDKLSQ
XP_045613573.1  ---------- ---LQEVLKV SPLKAWLMRH GH-S SAGKF AQLEL----- ---SNFQAPL VFQAET-PMR RFIAYIDPEN KFSEEDKLSQ
XP_021963432.1  ---------- ---TIENQVK SPLRGWLCNH GS-D SYDKF KQLET----- ---RGFKMPL LFTAET-PMK QFVAYIDPKN KFSVQDKLSQ
CAG7730563.1    ---------- ---CPVSQKR SALTGWLCNH GL-S SLEKL VTLQE----- ---NGFDQPL LFEGET-PLR KFVAYIDPEN KFTIEDKLSQ
```

```
               361
KAH8855123.1   ANVLQ QMIN VYSYEMLTKQ LQQNFIRVHG MWFELHSGDT LVYNRQNK-- SFIPINEATI SSLIDE V-- ---------- ----------
KAH9593836.1   ANVLQ QMIH VYSYEMLTNQ LAQNLLRIHG IWFELYSGRV LIYSRLNK-- AFLPITEETI TSLLHE L-- ---------- ----------
VDP71164.1     INVLQ QMSN VYAYPLLAEK LAAKALRVHG FWFSVHAGQM YVYNPQRE-- GFVPITEETL AELVRE SAK ---------- ----------
TPP65121.1     INVIQ QMSN VYSYPLLAQK LMNKELRVHG FWFNVHTGQM YLYSPHKQ-- GFLPIIEENL SEIMRE TAA
KAA0189413.1   INVLQ QMSN IYSYPLLSQK LMENEVRVHG LWFDIHTGQM YQFSRNKQ-- AFLPINEETL SELVRE SSS
KAF7258038.1   INVVQ QMSH VYSHPLLTER FKSGTLNVHG IWFHVHEAQL HMYSRPRQ-- SFVLITEVTI PELVAE LNC DSRHS----- ----------
KAG5442206.1   VNVVQ QMTN FYCQPLLAER LRNGSLNVHG LWFHIHSGQL HMFSREAQ-- SFVPVTGDTL PELVKE LDS P--------- ----------
XP_030837801.1 INVLQ QLEH LQSYDFIGKR METDQIRLHA TFYDTFSGNV YVFNQKQG-- RFNFLPTADI DSLSHY IFQ LRSS------ ----------
XP_041482585.1 INVLQ QLEH LQSYDFISKR TETDQIRLHA TFYDTFTGNM FVFNKVQS-- RFNILPTNDI TSLSHY IFQ LRSS------
KRZ73557.1     IHCLQ QLEN IASHHFLQQR LLEGLAKLHA VWFDVTEGKM YYFSRKHE-- EFVDINKDTI SYLIED ---
OUC42378.1     IHCLQ QLEN IASHHFLQKR LLEGLAKLHA VWFDVTEGKI LICEKNKV-- QYGKLLITNE NSSQCP LNT ANLLYQGDSI TLHVYGLTES
CDW52968.1     INCLQ QLEN IASHNFMASF LKSDKAFLHA LWLNFEDCVV HYFSKKKK-- KFVPITSESA AELQSE ---
KHJ46144.1     INCLQ QLEN IASHDFMASI LKSDKARLHA LWLNFEDCMV HYFSKKKK-- QFVAITSDSV TEFQNE ---
VZI00627.1     VNVLQ QMAN VYSHPILKDA IALGNVRVLG MWYNISTADV YLYSWLRR-- KFVLVDESSS HRLLEE YGS
XP_014681778.1 VNVLQ QMEN ISSLSFLQTH LGDELVNLHA WWFNLTTGEY HMFSPRDE-- RFIKLTEESA AAMLGI VGK E---------
VDM45293.1     INTLQ QMAN ITTHGFLAEI LKTKQADLHA FWFQVENAEM HIFSKKQH-- RFVIINEKTV DELLDE VEH HKA-------
KAF8385568.1   INVLQ QLVN IASHDALKEA FDQGKLHIHG MWFDVYKGED YLFSKEKR-- QFVIIKEDTF DSLIDE LHR RCNVPLGECP DIRQGHFT-P
PIO76965.1     INVLQ QLEN IASHEFLKQY FESGNLFLHG MWFDIYSGDD YLFSKDKK-- RFILID---- ------ --- ---------- ----------
VDL66086.1     INVLQ QLVN VSSHEFLQSY FESGLVFVHG MWFDIYNGDD YLFSKDKK-- QFVLIDESTV PDLLAE LES RCGPGLSSSE TAKA------
CAD6194288.1   VNVLQ QLVN IASHSFLSEH FEKKKLFVHG MWFDIYKGEV YIFSREKK-- RFIVIDDNSI DDLLKE LSQ YAPAEPNPPK DE--------
XP_045095542.1 INVLQ QIMN ISTHEFLKDY LEAGNLHLHG AWFNIYDGEV FLFSKDRK-- RFVVIDEKTV PSLSAE LER RCPLPEDKAG DVVIQNLH--
EGT43056.1     INVLQ QLIN ICSHEFLKEY LESGRLHIHG MWFDIYKGED YLFSKDKK-- RFVVIDEKTV TDLLAE LNT RYPLPEDQDG PVAFAQSN--
CAB3400100.1   INVLQ QMIN ISSHEFLKEY LENGKLHVHG MWFDVYKGED YLFSKDKK-- RFIVVNEETV SKLLDE LNA RCPVPTGENE KVAITHSN--
KAF7639475.1   INTLQ QLTN IASHEFLREH IEKRLVGLQA FWLDIFTGEL YFFSRRQE-- SFVPIDEENS ASLLKE ALC SVDD------ ----------
CAD5226593.1   INTLQ QLVH VASHGFLRDY LLEEKVHLHA LWFDVYKGDM HMFSRRKE-- RFVEIDEDSA AELENE VQT NYQEPIKAAA QV--------
XP_024510735.1 INTLQ QVLN IASHEFMFDY IANNEVFIHA MWFDIYTGNM YMFSKPEQ-- SFKMIDEMNT NHLLRY VED EMKGMKNNHQ KSNNCC----
VDN24694.1     INTLQ QMEN CASHGFLADI LANKAADLHA MWFDIFAGET YLFSRPRR-- KFILIDEKTV DSLQEE VKQ HVA-------
KAH7730640.1   INTLQ QLVH VASHGFLREF LEQGDVHLHA MWFDIYTGEM YLFSRERQ-- RFVVVNEQTS NELTAE IHR RMDDAAKPKP VQAANGAS--
TMS38138.1     INTLQ QLEN IASHGFLREF LESGSVNLHA MWFDIYTGNM FMFSKPRK-- MFVLVDEESV DGLIQE VDP EAGKSANLA- ----------
XP_013290959.1 INTLQ QLEN CASHGFLTEF LEKKTVDLHA MWFDIYTGEM YLFSKPRR-- KFILVNEDTV NELTEE VNQ HKA
RCN50005.1     INTLQ QLEN CASHGFLTEF LEKKTVDLHA MWFDIFAGEM YLFSKPRR-- KFILIDEGTV DKLEEE VNQ HKA
KJH42542.1     INTLQ QLEN VASHGFIVER LEQKTVNLHA MWFDIFTGEM YLFSKPRR-- MFILIDENSL NQLKTE VEQ C--------- ----------
VDM64099.1     VNTLQ QLEN IASHGFIAEH LEKRTVDLHA MWFDVYTGEM YLFSKPRR-- MFVVIDEDSV DLLKKE IEE HKA------- ----------
VDO63723.1     INTLQ QLEN IASHGFITEF LEKKTVDLHA MWFDIFAGEM YLFSKPQK-- EFVLIDEGSV DELEKQ IMK HVA------- ----------
VDO44040.1     INTLQ QLQN MASHGFITEM LEKKTVDLHA MWFDIFAGEM YMFSKPRK-- QFILIDEKSV DQLERE IEQ HKA------- ----------
CAD6198090.1   INTLQ QLEN VASHGFLKEF LETHAVNLHA MWFDIYTGEM HMFSKGKK-- RFILIDESTV EELLGE IEK TTR------- ----------
KAF8373880.1   INTLQ QLEN IASHGFLNEF LESRQVDLHA MWFDVYAGEM YMFSKPRK-- QFVLIDEESV AGLEDE VIQ HRS------- ----------
XP_002632400.2 INTLQ QLEN VASHGFLKEF LESQTVDLHA MWFDIYTGEM HMFSKPNN-- RFVLIEESNV EELIDE VEK HQT------- ----------
RDD39080.1     INTLQ QLLH VNSYSFMKEK IAAGTVKLYS LWFDIKDATC YVFNKRDK-- LFQPIVSEEM LKSVSE FQI NQK------- ----------
XP_001632619.1 VNVLQ QLHN LKSFPFISNP LSKGALNLYG LWFDIKEGEM YMFSRKQK-- KFVLINKDTV NNLCSE VD- ---------- ----------
XP_020895242.1 VNVLQ QLHN IGSYSFVREQ LRNRQLNLHA LWFDIANGEM YMFSKEKT-- SFQLVNEDTI KTIFKS V-- ---------- ----------
XP_027059688.1 VNVLQ QMQN IRSYGFVRNK LDEGSVQLYG LWFNIGEGEM LLYSKTRK-- RFVVINKETG LSSQ-- --- ---------- ----------
XP_029213515.2 VHVLE QMKN VKSYGFVREK LDTKALQVHG LWFDIGGGEM FMYSKMHN-- RFIVVNEQTL KTTFEH LTN EVIDKSLFS- ----------
GFO16554.1     FHCLQ QASN VASYPLLQPY LKKGEVKIHA MWFDVSKGQM HLFSPTNG-- YFIPIEETSA DELAKE ITS
RUS89945.1     FHTLQ QASH IASYPILRPY IATGDIRVHA MWFDIPRGEM HLFNASRG-- HFAPVTESEL EEWKDK EQS
GFR80859.1     FHCLQ QTSN VASYPILQPY IASGDVRVHA MWFDIFRAQM FMFSRTRG-- HFVAIEESSV DELREE AKS ---------- ----------
XP_005106142.1 LHCLQ QASH VSSYPFLHSL IQSGELTVNA LWFDVQSGYV YMFSPKDE-- RFVIVTEENL DTLLSI ITK
CAG5118574.1   INCLE QASH VTTYPLLQPF IKTGELQIHA MWYDVYTGNV YLFSPSER-- KFVIVSDSNV TSLMTE CGC NSCTGVLKEK QP--------
XP_013069769.1 LHCLQ QASH VQTYPMLKPF LDAGDFQIHA LWYDVYTGDV YMFSTSQN-- KFIEVNEKNI DIMNTD IAP LRI
XP_013069770.1 LHCLE QASH VKTYPMLHSY LSSGELHIHA LWFDVYTGNV YMFSVSQK-- KFILVDDNSF DILMQD VGE SPPSSF----
KAH9514813.1   FHCLV QASH VKTYPMLHPY VSSGELQIHV LWYDVFTGNV YMFSKSQR-- KYILVNDKNL PILLKD VED NVGLTC---- ----------
XP_033638319.1 VNVLQ QLEH LSSYRSISKR VDSGQLSVHG MWYDVSEDKI YWFSRQRK-- GFIEICADTI EEIIAS CKR TVRPEEEQGD L-GFKKPESV
XP_022095223.1 VSVLQ QLEH LSSYHSIRRR VIDGSLSLHG MWYDIAIDKI YWFSRQRK-- VFLEINEENI DEIVRS CQP SVADKASQND I-GHKIPS--
XP_038051194.1 VNVLQ QLEH LSSYRSIRRR VIAGTLSLHG MWYDVATDTV YWFSRARR-- MFLEINEDNI EEIVQD CQP TVTDTTTPND I-GFKVPS--
ACO15530.1     INTLV QIEN IYSYGFMKER MDQHQSVAHG LWLSLSSGEA HFFSKKDK-- AFVNVTEDNV EELVCR ---
XP_040566914.1 INTLV QMEN IYSYDFMKNR IDDKTAFVHG LWFSLTTGDV HYFLKNEK-- VFINVSEDNI DNLVQR SEE
VDP33652.1     IHCLQ QLEN IASYPIIRDP LESGKVKIHA MWFNIYTGEV SLFSRKNK-- KFVVVTEDTI NSVD-- --- ---------- ----------
PAA53847.1     VHCLQ QMIN LTTYPFLSER LLQRRVRLSA MWFDVDSADV YMFSRATE-- RFLVICEDNI EKLLPE PL- ---------- ----------
RNA13068.1     INTLV QLEN LKHYSFLKEP LETNKVRAYA LWLDIYNGDI LIFSFKEK-- RFVKLNDNTY ERLVSG INA A--------- ----------
CAF0715750.1   INTLI QLEN LKHYSFLKEP LEKKEIHPYA LWLDIYNGDI LVFCFKEK-- RFVKLNDESY QKLVSD LNM D--------- ----------
CAF0950609.1   INTLK QLKN VNNYNFLRDR ITKKLLRGYA LWTDITTNDV YMFSYKAK-- RFVKIDESTY NDLYKE CDT INQ------- ----------
CAF1119542.1   INTLE QLKN FLTYPFLKER LNRNELEVHA LWTDIYKGEV YMFSFQEK-- TFVKIDESTY KTLALQ CV- ---------- ----------
CAF1301217.1   VNALE QLKN FLSYPFLKDR LNRNELEVHA LWTDISKGEV YMFSFQEK-- TFVKIDETSY KTLALE CI-
CAF3941029.1   INTLE QLKN FLSYPFLKER LNRNELEVHA LWTDISEGEV YMFSFKEK-- TFVKIDEISY KTLSLE CI- ---------- ----------
CAF0867361.1   VNTLE QMRH FLSYPFLRER LNRKELQVHA LWTDIYKGEV YMFSFKEK-- TFIKIDENTY NKLSLE CNP YK-------- ----------
UJR16523.1     VNTLE QLRH FLSYPFLKDR LNRKELQVHA LWTDIYKGEV YMFSFKEK-- CFIKIDENTY KKLSFE CTP NK-------- ----------
CAF1340180.1   VNTLE QLQH FLSYPFLRER LNRKQLQVHA LWTDIYKGEV YMFSFNDK-- CFVKIDDETY KSLAHE CN- ---------- ----------
CAF1025683.1   VNTLE QLRH FLSYPFLRER LNRKELQVHA LWTDIYRGEV YMFSFKEK-- CFVKIDENTY EKLSHE CI- ---------- ----------
ALS04255.1     VNTLI QMQN VTTYPFMHSC LRDGRTHIHA LWFDVYTGEI HCFSRKEK-- TFIPVNENTV EELIN- --- -----ELTES AARFKVNSNF
XP_023329171.1 VNTIV QMQN VTSYPFMHNC LRDGRTHIHA LWFDVYTGEV HCFSRKEKVK EKKEFDKNMI KQSKKT HSK PIK--RISIE TKNWYIRKET
KAH3870454.1   VNCIQ QLEN IAAYPFLKER LSQNDLLLHA FWYDIHSGNV FLLSRERQ-- RFVEVNERSY FGLMQE VKN RASAKILSES ----------
XP_045201158.1 INCVQ QLEN IAAYPFLKDR LSKKDLFLHA LWYDVHSGNV FLLSKKRQ-- KFVEVNERSF FSLIEE VQS VEGG------ ----------
XP_009053308.1 VNCLQ QLHH ISTYPFLKLK LMTGQLFLHA FWFDVHSADV YMFSRSRK-- QFIPVSDDTY DHLLED CHK QK-------- ----------
XP_033096127.1 VNTLQ QLVN LSTHTMIKPQ LHDGRVRLHA MWFDIFTGNM FIFSSELQ-- RFIEIREDTI DHLLSR VKH NESKDHIETL TNSVKMSK--
XP_013785798.1 INCLQ QLEN ATSHAFLKPL LEQDKLRFHA LWFDINTGDV HCFSNKKE-- QFVEVNDHSV EELLVE SDQ NIFSGPNK-- ----------
GAU97340.1     VSTLH QLEN ITSYPFMQRL VDSGLVRLHA MWFDIYTGSI FYFNKPHK-- TFIEITEKNW DEFLNE KDR EALKKQQIFG LRNLVI----
OQV24824.1     VSTLH QLEN ITSYPFMQRL VDGGLVRLHA MWFDIYTGNI FYFSKPHK-- SFIEITEENW DDFLDE KER EALNKQVKFG MRQNLMT---
CAD5123161.1   VNTLQ QISH ISAFPIIKKS LEEGHIKIHA LWFNTFTGDV LMFSRQKG-- EFINITEDTY LDLAQD GES Y--------- ----------
ELT97609.1     VNVLQ QVEN ACSYDMLKDK LQSGQVNVTA LWFNIQTADF FMFSKEKE-- RFLEVNESTI DHLVSD AGG H--------- ----------
XP_009013880.1 VNTLQ QLQN IASYPFLRDK LESGLVRMHA LWIDIYTGDF YMFCRDYN-- RFVLVDEESY QNLLDD GES VTDY------ ----------
KAI0233246.1   VNTLQ QLEN IVSHGFMKEH LGSDKVRLNA MWFDIYTGDV YMFSRERQ-- SFIQVTKATY AHLVSD GES W--------- ----------
XP_029642194.1 VNCLQ QLQN IATFPILHEK LSKNQIRLHA IWIDVRTGNV FIFSRDEK-- QFVEITERNY LQLVRE CFP VD-------- ----------
NP_001171747.1 VNCLQ QLQN ISSYGFLKDR LESGRVRLHA MWFDIYAGDV YLFSRQAK-- RFVEINEETC NDLLKD CEH QKCHPIED-- ----------
CAH1781438.1   VHCLQ QLQN IASQDFLKEK LSKNEVHLHA MWYDMKLADM LIFSRQRK-- QFVTVNEDNY LHLLED SMT MPDHKEMEKP SPVWRG----
XP_013382323.1 INTLN QLQN IASYPFLKEK LQTGKVRLHA LWFDIHSGNV YIFSKMEK-- KFVEINEENY GMLMED SVK DPKIKHY--- ----------
XP_033759917.1 VNCLQ QLQN VASYPFLKDL ISREEVKLHA MWFDIYTGEV FMFSRMQQ-- RFIEINDQNI NHLLHD AYR KVEKIPITEQ RRK-------
XP_041373453.1 VNCLQ QLQN IASYPFLREK LMRDEVRLHA MFFDIYTGNV YMFSRKEK-- QFIEICKENY NALLKD ADS LEVNEE---- ----------
XP_046548515.1 VNCLQ QLQN IASYDFLREK LHRNDVRLHA MWFDIYTGNC FMFSRERK-- MFVEITEDNY AHLIKD AHS DLH------- ----------
XP_047034441.1 VNTLQ QLSN IASYGMLKKR LEKYDLHIHA LWFDIYTGDI FYFSRRYN-- RFVIIDESSY EELAAE VRR YYS------- ----------
CAB3245311.1   VNTLQ QLQN IASYGMLKKR LEKHDLHIHA LWFDIYTVCR YKIVTMSNRP TFDYNDESQS ERLARK SKD SPFMIIGIIG LIGVCGFGAY
XP_011549456.2 VNTLQ QLQN VASYGMLKKR LQKHDLHIHA LWFDIYTGDI YYFSRRAK-- TFVIIDESTY ENILSE VRR YYS------- ----------
XP_045761472.1 VNTLQ QLQN IASYGMLKKR LQRHDLHIHA LWFDIYTGDI YYFSRRAK-- KFLIIDESSY EIILAE IRR YYS------- ----------
XP_046968321.1 VNTLQ QLQN IASYGMLKKR LERHDLHIHA LWFDIYTGDI YYFSRRAK-- RFLIIDESSY EIILAE IRR YYS------- ----------
XP_026323335.1 VNTLQ QLQN VASYGILKKR LEKHDLHIHA LWFDIYTGDI YYFSRRAK-- RFIIIDESTY EEILAE IRR YYS------- ----------
XP_045529909.1 VNTLQ QVQN VASYGMLKKR LEKHDLHIHA LWFDIYTGDI YYFSRRAK-- SFVIIDESTY ESILAE VRR YYS------- ----------
RVE42816.1     VNTLQ QLQN VASYGMLKKR LQKHDLHIHA LWFDIYTGDI YFFSRRAK-- RFIIIDESSY ESILSE VKR YYS------- ----------
KAF9415294.1   VNTLQ QLQN VASYGMLKKR LEKHDLHIHA LWFDIYTGDI YYFSRRAK-- RFIIIDESSY ESILAE VRR FYS------- ----------
XP_018897307.1 INCLQ QLQN IASYGFLKKR LESHDLHIHA LWFDIYTGDI YYFSRQNK-- KFVEINEMTV DMLEQE VLR FYS------- ----------
```

```
XP_019763455.1   INTLQ QLQN IASYGFLKKR LERNQLHIHA LWFDIYTGEI YYFSRGAK-- RFVIIDESNF EKLTEE VVL YYS------- ----------
XP_030749737.1   INTLQ QLQN IASYGFLRKK LEKHQLHIHA LWFDIYTGEI YYFSRRHK-- RFIVIDEENF AKLFEE VIV YYS------- ----------
KAF7270051.1     INTLQ QLQN IASYGFLKKK LENHQLHIHA LWFDIYTGEI YYFSRRHK-- RFVIIDEEHF DKLFKE VIV YYS------- ----------
XP_031343879.1   IHTLQ QVQN IASYGFLKKR LETDQIHIHA MWFDIYTGEI YYFSRQAK-- RFVVIDENNF DRLVGE VER YHM------- ----------
XP_044743527.1   INTLQ QLQN ISSYGFLKKR LEQYDLHIHA LWFDIYTGEI YYFSRRAK-- QFVQINEHNY NVLLQE IQT YYS------- ----------
XP_018319432.1   INALQ QLQN IASYGFLKSR LEKHQLHIHA LWFDIYTGEI YYFSRQAK-- QFVIINEDNI ETLMDE VEN YYM------- ----------
XP_026466152.1   VNTLQ QLQN IASYGFLRKR LEKHDLHIHA LWFDIYTGEI YYFSRQAK-- TFVLIDETSF NYLMKE VCK YYS------- ----------
XP_022913687.1   VNTLQ QLQN IASYGFLKKR LETNELHIHA LWFDIYTGEI YYFSRAAK-- TFVCINEESI DKILEE VEE YYT------- ----------
KAF5298473.1     INTLQ QLQN IASYGFLKKR LETHQLHLHA LWFDIYTGEI YYFSRQAK-- RFIIIDEKNF DKLLME VEK YYM------- ----------
VEN57246.1       INTLQ QLQN IASYGFLKRR LEKHQLHIHA VWFDIYTGDI YYFSRAAK-- RFVPVDESNI ERLMDE VRR YYS------- ----------
XP_045472083.1   INTLQ QLQN IASYGFLKKR LETHQLHIHA LWFDIYTGEI YYFSRGAK-- KFVVIDEGNI EKLLDE VDN YYS------- ----------
XP_017768645.1   INTLQ QLQN IASYGFLKKR LEKHELHIHA LWFDIYTGEI YYFSRGSK-- TFVLIDEHNI DALVGE VKK YYS------- ----------
CAH1369701.1     INTLQ QLQN IASYGFLKKR LEKHQLHIHA LWFDIYTGEI YYFSRGAK-- RFVVVDEDNI DKLMGE VGK YYS------- ----------
XP_028136465.1   INTLQ QLQN IASYGFLKKR LEKHELHIHA LWFDIYTGDI YYFSRGAK-- RFVLIDEDNV DKLLQE VKK YYS------- ----------
XP_023014099.1   INTLQ QLQN IASYGFLKKR LEKHELHIHA LWFDIYTGEI YYFSRAAK-- KFVIIDENNF DKLLSE VER YYS------- ----------
XP_019875064.1   INTLQ QLQN IASYGFLKRR LEKHQLHIHA LWFDIYTGDI YYFSRAAK-- KFVIIDENNF EKLFLE VNK YYS------- ----------
CAH0557060.1     INTLQ QLQN IASYGFLKRR LEKHQLHIHA LWFDIYTGEI YYFSRAAK-- KFVVIDEENV EKLLNE VNT YYS------- ----------
XP_037924690.1   INTLQ QLSN VASYGFLKHR LEQHDLHIHA LWFDIYTGDI YFFSRGAK-- QFVHVDESTV GKLLQE VNR YYS------- ----------
XP_043863257.1   INTLQ QMSN IASYGFLKTR LESHNLHIHA LWFDIYTGDI YYFSRGAK-- RFVAVDESSV DQLSAE VRR FYS------- ----------
XP_039965629.1   INTLQ QLSN VASYGFLKPR LESHDLHIHA LWFDIYTGDV YYFSRGAK-- RFIPVDESTV GKLTEE VRR YYS------- ----------
XP_001989827.1   INTLQ QMSN VASYGFLKSR LESHNLHIHA LWFDIYTGDI YYFSRGAK-- RFIAVDESSV EQLSAE VRR FYS------- ----------
XP_037896985.1   INTLQ QVSN IASYGFLKPR LESHDLHIHA LWFDIYTGDI YYFSRGSK-- RFVPVDEQSV EKLTEE VKR YYS------- ----------
XP_046805044.1   INTLE QMSN IASYGFLKAR LESHDLHIHA LWFDIYTGDI YYFSRGAK-- RFIPVDEDTV DKLTSE VRR FYS------- ----------
KAG5681791.1     INTLQ QLQN IASYGFLKKR LEQFDLHIHA LWFDIYTGDI YYFSRQMK-- RFVIISEETI DKISED VKK YYS------- ----------
XP_031629647.1   INTLE QLSN VASYGFLKRR LETHDLHIHA LWFDIYTGDI YYFSRGQK-- RFVEVNEENS QQIIEE VNR FYS------- ----------
KAG4077403.1     INTLE QLSN VASYGFLKKR LETHDLHIHA LWFDIYTGDI YYFSRGSK-- CFVAVDESSV DRLLDE VAQ LDETK----- ----------
XP_039430644.1   VNTLQ QIEN IASYGFLKKR LESHDLHIHA LWFDIYTGDI YYFSRNSK-- RFIPVDETTI ERLLKE VNQ FYS------- ----------
XP_040221405.1   VNTLQ QIEN VASYGFLKRR LESHDLHIHA LWFDIYTGDI YFFSRNSK-- RFIAIDESSI DRLLDE VRT ---------- ----------
CAA9998239.1     VNTLE QLAN VASYNFLKRR LESHQLHVHA LWFDIYTGDI YYFSRQNK-- RFVEINEFTV DSLSDE VSR YYS------- ----------
KAF6203636.1     VNTLE QLAN VASYNFLRRR LETHQLHVHA LWFDIYTGDI YYFSRQNK-- RFVEINEATV DKLSEE VRR DGNGEGSNSE AFPLV-----
KAG6800307.1     INTLQ QLQN IASYGFLKKR LERHDLHIHA LWFDIYTGDI YYFSRANK-- KFVEINESTE RYLLTE IKK YYS------- ----------
XP_014230599.1   VNTLQ QLQN VASYGFLKKR LETHDLHIHA IWFDIYTGDI YYFSRAHK-- RFVEINELNE TPILKE IKT YYS------- ----------
KAH0947068.1     INTLQ QLQN IASYGFLKKR LEKHDLHVHA LWFDIYTGDI YYFSRASK-- RFVEINETTE SHLLKE IMT YYS------- ----------
XP_043590797.1   INTLQ QLQN VASYGFLKKR LERHDLHIHA LWFDIYTGDI YYFSRANK-- RFVEINESTE ASLLAE IKK YYS------- ----------
XP_034949379.1   INTLQ QLQN IASYGFLKKR LEQHDLHIHA LWFDIYTGDI YYFSRGNK-- RFIEINELTE SQLLEE ILK YYS------- ----------
XP_012275169.1   INTLQ QLQN VASYGFLKKR LERHELHIHA LWFDILTGEI HYFSRANK-- RFIEINERTE ASLLEE IHK YYS------- ----------
XP_043464528.1   INTLQ QLQN VASYGFLKKR LERHDLHIHA LWFDIYTGDI YYFSRANK-- RFIEVNENTE KLLLKE IKK YYT------- ----------
XP_014220045.1   INTLQ QLQN VASYGFLKQR LEKHDLHVHA LWFDIYTGDI YYFSRAGK-- RFVEINEMTE TPLLKE IKK YYS------- ----------
XP_011160605.3   INTLQ QLQN IASYGFLKKR LERHDLHIHA LWFDIYTGDI YYFSRANK-- RFVEINETTE PLLLKE IKQ YYS------- ----------
XP_046741789.1   INTLQ QLQN VASYGFLKRR LERHDLHIHA LWFDIYTGDI YYFSRANK-- RFIEINELTE TPLLRE IKK YYS------- ----------
VVC24333.1       VNTLQ QLQN IASYGFLRKR LESHDLHIHA LWFDIYTGDI YYFSRQKK-- KFVEINEMNV EKLVEE VSK YYC------- ----------
KAF0770685.1     INTLQ QIQN IASYDFLKKR LETYDLHIHA LWFDIYTGDI HYFSRQSK-- QFVEINEKNV DRLVEE VSK YYC------- ----------
XP_015365968.1   VNTLQ QIQN IASYDFLKKR LEAYDLHIHA LWFDIYTGEI HYFSRQNK-- QFVEINEKNV NRLVEE VSK YYC------- ----------
XP_029341681.1   VNTLQ QIQN IASYDFLKKR LETYDLHIHA LWFDIYTGDV HYFSRQSK-- QFVEINEKNV DGLVEE VSK YYC------- ----------
XP_026292519.1   VHCLQ QMQN IASYSFLKPR LEKHELHIHA LWFDIYSGDI YYFSRGEK-- RFVEINDSTI DPLLKE VKR YYS------- ----------
XP_034238427.1   VHCLQ QMQN IASYSFLKSR LEKHELHIHA LWFDIYSGDI YYFSRGEK-- RFIEINENTI DPLLKE VKR YYS------- ----------
XP_024081347.1   INCLQ QLAN IASYGFLKRR LEKYELHIHA LWFDIYTGDI FYFSRQNK-- RFVEINEDTV DKLSLE Q-- ---------- ----------
XP_014283188.1   VNCLQ QMAN IASYGFLKRR LERHQLHIHA LWFDIYTGNI YYFSRQNK-- RFIDINEETV DMLSHE VKK YYS------- ----------
RZF32743.1       VNCLQ QVQN IASYGFLKKR LETHQLHIHA LWFDIYSGDI FFFSRQNK-- RFIEINENTV EKLTDE VNR YYS------- ----------
CAB3380083.1     INCLQ QIQN VASYGFLKRR LETHDLHLHA LWFDIYTGDV YYFSRQRK-- RFVEINDLTA RPILKE IRR YYS------- ----------
XP_046989067.1   VNCLQ QLQN IASYGFLKKR LETHQLHIHA LWFDIYTGDI YYFSRQNK-- RFVEINEHTV MGLIKE VNR YYS------- ----------
GFG38476.1       VNCLQ QLQN IASYGFLKRR LETHELHIHA LWFDIYTGDI YYFSRQNK-- RFIDINEGTF SMLEKE VLK YYS------- ----------
CAD7446618.1     INCLQ QLQN IASYGFLKKR LERHDLHIHA LWFDIYTGDI YFFSRQSK-- RFVEINETTI KQLLLE VKT YYS------- ----------
KAG8226740.1     VNCLQ QLQN IASYGFLKRR LETHSLHIHA LWFDIYTGDI FYFSRQQK-- QFVEINEETS KTLLKE IKR YYS------- ----------
XP_046392794.1   VNCLQ QLQN IASYGFLKQR LETHQLHIHA IWFDIYTGDI FYFSRQQK-- RFVEINEETY NLLLKE ILR YYS------- ----------
XP_046459477.1   LNTLQ QLQH IASYSFIQSA INSGRVHLHA LWFDIYTGDI YVFSRKQK-- RFVEISEETS DYLLDE IRE YFV------- ----------
XP_043227363.1   VNTLQ QLQN VSSYGMLKRG LTRGEIHIHA LWFDIYTGNI HYFSRQQK-- RFVDLNEDTL PQISEE VRT YYSSGR---- ----------
XP_018006429.1   VNCLQ QLQN IASYNFMRDG LAKGRVYLHA FWFDIYSGDI HYFSRQQK-- TFVPMNEDTI DTLMDE VHK YYS------- ----------
AWH57222.1       VNTLQ QLQN IASYNFMRDG LVRGRVYIHA LWFDIYTGDI YYFSRQQK-- KFVDVSENNI ERLLDE AKQ YYT------- ----------
XP_042865604.1   VNTLQ QLQN IASYNFMREG LSRGKVYIHA LWFDIYTGDI YYFSRQQK-- MFVDVNEGNI EKLLEE VVK YYT------- ----------
XP_045119740.1   VNTLQ QLQN IASYNFMREG LLRNRVYIHA LWFDIYTGDI YYFSRQQK-- MFVDINETNI EKLLDE VLK YYT------- ----------
XP_045613573.1   VNTLQ QLQN IASYNFMREG LSRGRVYIHA LWFDIYTGDI YYFSRQQK-- MFVDVNESNM EKLEEE VFK YYT------- ----------
XP_021963432.1   VNCLQ QLQN IASYGFLREG LASGKIFIHA LWFDIYTGDI YYFSRQKK-- EFVDINETTF PKLLEE VSE FYYH------ ----------
CAG7730563.1     VNCLQ QLQN IASYGFMRDI LMQGKTFIHA MWFDIYTGDI YYFSRQKK-- QFVEINEENY YGLLEE VMT YYSQYD---- ----------
```

```
                 451
KAH8855123.1     ---------- ---------- ---------- ------- -- ---------- --
KAH9593836.1     ---------- ---------- ---------- ------- -- ---------- --
VDP71164.1       ---------- ---------- ---------- ------- -- ---------- --
TPP65121.1       ---------- ---------- ---------- ------- -- ---------- --
KAA0189413.1     ---------- ---------- ---------- ------- -- ---------- --
KAF7258038.1     ---------- ---------- ---------- ------- -- ---------- --
KAG5442206.1     ---------- ---------- ---------- ------- -- ---------- --
XP_030837801.1   ---------- ---------- ---------- ------- -- ---------- --
XP_041482585.1   ---------- ---------- ---------- ------- -- ---------- --
KRZ73557.1       ---------- ---------- ---------- ------- -- ---------- --
OUC42378.1       ---------- VPYWR----- ---------- ------- -- ---------- --
CDW52968.1       ---------- ---------- ---------- ------- -- ---------- --
KHJ46144.1       ---------- ---------- ---------- ------- -- ---------- --
VZI00627.1       ---------- ---------- ---------- ------- -- ---------- --
XP_014681778.1   ---------- ---------- ---------- ------- -- ---------- --
VDM45293.1       ---------- ---------- ---------- ------- -- ---------- --
KAF8385568.1     -----G---- ---------- ---------- ------- -- ---------- --
PIO76965.1       ---------- ---------- ---------- ------- -- ---------- --
VDL66086.1       ---------- ---------- ---------- ------- -- ---------- --
CAD6194288.1     ---------- ---------- ---------- ------- -- ---------- --
XP_045095542.1   ---------- ---------- ---------- ------- -- ---------- --
EGT43056.1       ---------- ---------- ---------- ------- -- ---------- --
CAB3400100.1     ---------- ---------- ---------- ------- -- ---------- --
KAF7639475.1     ---------- ---------- ---------- ------- -- ---------- --
CAD5226593.1     ---------- ---------- ---------- ------- -- ---------- --
XP_024510735.1   ---------- ---------- ---------- ------- -- ---------- --
VDN24694.1       ---------- ---------- ---------- ------- -- ---------- --
KAH7730640.1     ---------- ---------- ---------- ------- -- ---------- --
TMS38138.1       ---------- ---------- ---------- ------- -- ---------- --
XP_013290959.1   ---------- ---------- ---------- ------- -- ---------- --
RCN50005.1       ---------- ---------- ---------- ------- -- ---------- --
KJH42542.1       ---------- ---------- ---------- ------- -- ---------- --
VDM64099.1       ---------- ---------- ---------- ------- -- ---------- --
VDO63723.1       ---------- ---------- ---------- ------- -- ---------- --
VDO44040.1       ---------- ---------- ---------- ------- -- ---------- --
CAD6198090.1     ---------- ---------- ---------- ------- -- ---------- --
KAF8373880.1     ---------- ---------- ---------- ------- -- ---------- --
XP_002632400.2   ---------- ---------- ---------- ------- -- ---------- --
RDD39080.1       ---------- ---------- ---------- ------- -- ---------- --
XP_001632619.1   ---------- ---------- ---------- ------- -- ---------- --
XP_020895242.1   ---------- ---------- ---------- ------- -- ---------- --
XP_027059688.1   ---------- ---------- ---------- ------- -- ---------- --
XP_029213515.2   ---------- ---------- ---------- ------- -- ---------- --
GFO16554.1       ---------- ---------- ---------- ------- -- ---------- --
RUS89945.1       ---------- ---------- ---------- ------- -- ---------- --
GFR80859.1       ---------- ---------- ---------- ------- -- ---------- --
XP_005106142.1   ---------- ---------- ---------- ------- -- ---------- --
CAG5118574.1     ---------- ---------- ---------- ------- -- ---------- --
XP_013069769.1   ---------- ---------- ---------- ------- -- ---------- --
XP_013069770.1   ---------- ---------- ---------- ------- -- ---------- --
KAH9514813.1     ---------- ---------- ---------- ------- -- ---------- --
XP_033638319.1   -----GTAIE ---------- ---------- ------- -- ---------- --
XP_022095223.1   ---------- ---------- ---------- ------- -- ---------- --
XP_038051194.1   ---------- ---------- ---------- ------- -- ---------- --
ACO15530.1       ---------- ---------- ---------- ------- -- ---------- --
XP_040566914.1   ---------- ---------- ---------- ------- -- ---------- --
VDP33652.1       ---------- ---------- ---------- ------- -- ---------- --
PAA53847.1       ---------- ---------- ---------- ------- -- ---------- --
RNA13068.1       ---------- ---------- ---------- ------- -- ---------- --
CAF0715750.1     ---------- ---------- ---------- ------- -- ---------- --
CAF0950609.1     ---------- ---------- ---------- ------- -- ---------- --
CAF1119542.1     ---------- ---------- ---------- ------- -- ---------- --
CAF1301217.1     ---------- ---------- ---------- ------- -- ---------- --
CAF3941029.1     ---------- ---------- ---------- ------- -- ---------- --
CAF0867361.1     ---------- ---------- ---------- ------- -- ---------- --
UJR16523.1       ---------- ---------- ---------- ------- -- ---------- --
CAF1340180.1     ---------- ---------- ---------- ------- -- ---------- --
CAF1025683.1     ---------- ---------- ---------- ------- -- ---------- --
ALS04255.1       -----GDAMP TGYRKD--VS DEETLQKTA- QRMMHTA RQ TPHVCDQNCK HD
XP_023329171.1   -----NKATE KVLRKG--NY TRKRLIKK-- ------L RK PI-------- --
KAH3870454.1     ---------- ---------- ---------- ------- -- ---------- --
XP_045201158.1   ---------- ---------- ---------- ------- -- ---------- --
XP_009053308.1   ---------- ---------- ---------- ------- -- ---------- --
XP_033096127.1   ---------- ---------- ---------- ------- -- ---------- --
XP_013785798.1   ---------- ---------- ---------- ------- -- ---------- --
GAU97340.1       ---------- ---------- ---------- ------- -- ---------- --
OQV24824.1       ---------- ---------- ---------- ------- -- ---------- --
CAD5123161.1     ---------- ---------- ---------- ------- -- ---------- --
ELT97609.1       ---------- ---------- ---------- ------- -- ---------- --
XP_009013880.1   ---------- ---------- ---------- ------- -- ---------- --
KAI0233246.1     ---------- ---------- ---------- ------- -- ---------- --
XP_029642194.1   ---------- ---------- ---------- ------- -- ---------- --
NP_001171747.1   ---------- ---------- ---------- ------- -- ---------- --
CAH1781438.1     ---------- ---------- ---------- ------- -- ---------- --
XP_013382323.1   ---------- ---------- ---------- ------- -- ---------- --
XP_033759917.1   ---------- ---------- ---------- ------- -- ---------- --
XP_041373453.1   ---------- ---------- ---------- ------- -- ---------- --
XP_046548515.1   ---------- ---------- ---------- ------- -- ---------- --
XP_047034441.1   ---------- ---------- ---------- ------- -- ---------- --
CAB3245311.1     KYKNRGKMST SVFLMQLRVA AQGTVVSALT IGLAYTL AK -EHLFKDDKK --
XP_011549456.2   ---------- ---------- ---------- ------- -- ---------- --
XP_045761472.1   ---------- ---------- ---------- ------- -- ---------- --
XP_046968321.1   ---------- ---------- ---------- ------- -- ---------- --
XP_026323335.1   ---------- ---------- ---------- ------- -- ---------- --
XP_045529909.1   ---------- ---------- ---------- ------- -- ---------- --
RVE42816.1       ---------- ---------- ---------- ------- -- ---------- --
KAF9415294.1     ---------- ---------- ---------- ------- -- ---------- --
XP_018897307.1   ---------- ---------- ---------- ------- -- ---------- --
```

```
XP_019763455.1  ---------- ---------- ---------- ------- -- ---------- --
XP_030749737.1  ---------- ---------- ---------- ------- -- ---------- --
KAF7270051.1    ---------- ---------- ---------- ------- -- ---------- --
XP_031343879.1  ---------- ---------- ---------- ------- -- ---------- --
XP_044743527.1  ---------- ---------- ---------- ------- -- ---------- --
XP_018319432.1  ---------- ---------- ---------- ------- -- ---------- --
XP_026466152.1  ---------- ---------- ---------- ------- -- ---------- --
XP_022913687.1  ---------- ---------- ---------- ------- -- ---------- --
KAF5298473.1    ---------- ---------- ---------- ------- -- ---------- --
VEN57246.1      ---------- ---------- ---------- ------- -- ---------- --
XP_045472083.1  ---------- ---------- ---------- ------- -- ---------- --
XP_017768645.1  ---------- ---------- ---------- ------- -- ---------- --
CAH1369701.1    ---------- ---------- ---------- ------- -- ---------- --
XP_028136465.1  ---------- ---------- ---------- ------- -- ---------- --
XP_023014099.1  ---------- ---------- ---------- ------- -- ---------- --
XP_019875064.1  ---------- ---------- ---------- ------- -- ---------- --
CAH0557060.1    ---------- ---------- ---------- ------- -- ---------- --
XP_037924690.1  ---------- ---------- ---------- ------- -- ---------- --
XP_043863257.1  ---------- ---------- ---------- ------- -- ---------- --
XP_039965629.1  ---------- ---------- ---------- ------- -- ---------- --
XP_001989827.1  ---------- ---------- ---------- ------- -- ---------- --
XP_037896985.1  ---------- ---------- ---------- ------- -- ---------- --
XP_046805044.1  ---------- ---------- ---------- ------- -- ---------- --
KAG5681791.1    ---------- ---------- ---------- ------- -- ---------- --
XP_031629647.1  ---------- ---------- ---------- ------- -- ---------- --
KAG4077403.1    ---------- ---------- ---------- ------- -- ---------- --
XP_039430644.1  ---------- ---------- ---------- ------- -- ---------- --
XP_040221405.1  ---------- ---------- ---------- ------- -- ---------- --
CAA9998239.1    ---------- ---------- ---------- ------- -- ---------- --
KAF6203636.1    ---------- -----RSVLS S--------- ------- -- ---------- --
KAG6800307.1    ---------- ---------- ---------- ------- -- ---------- --
XP_014230599.1  ---------- ---------- ---------- ------- -- ---------- --
KAH0947068.1    ---------- ---------- ---------- ------- -- ---------- --
XP_043590797.1  ---------- ---------- ---------- ------- -- ---------- --
XP_034949379.1  ---------- ---------- ---------- ------- -- ---------- --
XP_012275169.1  ---------- ---------- ---------- ------- -- ---------- --
XP_043464528.1  ---------- ---------- ---------- ------- -- ---------- --
XP_014220045.1  ---------- ---------- ---------- ------- -- ---------- --
XP_011160605.3  ---------- ---------- ---------- ------- -- ---------- --
XP_046741789.1  ---------- ---------- ---------- ------- -- ---------- --
VVC24333.1      ---------- ---------- ---------- ------- -- ---------- --
KAF0770685.1    ---------- ---------- ---------- ------- -- ---------- --
XP_015365968.1  ---------- ---------- ---------- ------- -- ---------- --
XP_029341681.1  ---------- ---------- ---------- ------- -- ---------- --
XP_026292519.1  ---------- ---------- ---------- ------- -- ---------- --
XP_034238427.1  ---------- ---------- ---------- ------- -- ---------- --
XP_024081347.1  ---------- ---------- ---------- ------- -- ---------- --
XP_014283188.1  ---------- ---------- ---------- ------- -- ---------- --
RZF32743.1      ---------- ---------- ---------- ------- -- ---------- --
CAB3380083.1    ---------- ---------- ---------- ------- -- ---------- --
XP_046989067.1  ---------- ---------- ---------- ------- -- ---------- --
GFG38476.1      ---------- ---------- ---------- ------- -- ---------- --
CAD7446618.1    ---------- ---------- ---------- ------- -- ---------- --
KAG8226740.1    ---------- ---------- ---------- ------- -- ---------- --
XP_046392794.1  ---------- ---------- ---------- ------- -- ---------- --
XP_046459477.1  ---------- ---------- ---------- ------- -- ---------- --
XP_043227363.1  ---------- ---------- ---------- ------- -- ---------- --
XP_018006429.1  ---------- ---------- ---------- ------- -- ---------- --
AWH57222.1      ---------- ---------- ---------- ------- -- ---------- --
XP_042865604.1  ---------- ---------- ---------- ------- -- ---------- --
XP_045119740.1  ---------- ---------- ---------- ------- -- ---------- --
XP_045613573.1  ---------- ---------- ---------- ------- -- ---------- --
XP_021963432.1  ---------- ---------- ---------- ------- -- ---------- --
CAG7730563.1    ---------- ---------- ---------- ------- -- ---------- --
```
